# Nocaviogua A and B: two lipolanthines from root-nodule-associated *Nocardia* sp.

**DOI:** 10.3389/fchem.2023.1233938

**Published:** 2023-08-03

**Authors:** Shanshan Chang, Yajun Luo, Ning He, Xinyue Huang, Mingxu Chen, Lijie Yuan, Yunying Xie

**Affiliations:** ^1^ CAMS Key Laboratory of Synthetic Biology for Drug Innovation, Institute of Medicinal Biotechnology, Chinese Academy of Medical Sciences and Peking Union Medical College, Beijing, China; ^2^ Hebei Key Laboratory for Chronic Diseases, Tangshan Key Laboratory for Preclinical and Basic Research on Chronic Diseases, School of Basic Medical Sciences, North China University of Science and Technology, Tangshan, Hebei, China

**Keywords:** lipolanthine, *Nocardia*, advanced Marfey’s analysis, NMR calculations, cytotoxicity

## Abstract

Nocaviogua A (**1**) and B (**2**), two lipolanthines featuring a non-canonical avionin (Avi)-containing macrocycle and a long acyl chain, were identified from the mutualistic actinomycete *Nocardia* sp. XZ19_369, which was isolated from the nodules of sea buckthorn collected in Tibet. Their planar structures were elucidated via extensive analyses of 1D and 2D NMR, as well as HRMS data. The absolute configurations were fully elucidated by advanced Marfey’s analysis and GIAO NMR calculations, representing the first time that the configurations of this family of lipolanthines have been determined. Nocaviogua A (**1**) exhibited weak cytotoxicity against human chronic uveal melanoma cells (UM92-1), non-small cell lung cancer (NCI-H2170), and breast cancer (MDA-MB-231). Our work provides valuable information on this burgeoning class of lipolanthines for further investigations.

## 1 Introduction

Ribosomally synthesized and post-translationally modified peptides (RiPPs) are a large group of natural products that give rise to a potent biological activity and desirable drug-like properties, such as resistance to chemical and enzymatic degradation (Watrous et al., 2012; Montalban-Lopez et al., 2021; Mazo et al., 2023), conformational rigidity (Bobeica et al., 2020), and high target specificity (Ongey et al., 2017; Grant-Mackie et al., 2021). The structural diversity of RiPPs is multiplied by fusions with polyketides or fatty acids (Kozakai et al., 2020). Among them, lipolanthines are a class of polyketide/fatty acid–ribosomally synthesized hybrid lipopeptides (PK/FA–RiPPs) that feature a unique avionin (Avi)-containing macrocycle and a long acyl chain (Wiebach et al., 2018; Kozakai et al., 2020). Due to the utilization of class-III-type lanthipeptide synthetases in biosynthesis, these unusual natural lipopeptides were also classified as class-Ⅲ lanthipeptides (Grant-Mackie et al., 2021).

Since the first lipolanthine was reported in 2018 (Wiebach et al., 2018), only 12 lipolanthines, belonging to three groups (microvionin, nocavionin, and goadvionins), have been discovered, and none of their absolute configurations have been fully determined. As an emerging class of natural products, some of these lipolanthines have shown promising antibacterial effects against antibiotic-resistant bacteria, e.g., microvionin has exhibited strong antibacterial effects with MIC values of less than 0.46 *μ*g/mL against MRSA and less than 0.15 *μ*g/mL against *Streptococcus pneumoniae* (Wiebach et al., 2018); goadvionins have also been shown to inhibit the growth of Gram-positive bacteria, with goadvionin B2 displaying the MIC values of 6.4 *μ*g/mL against *Staphylococcus aureus*, 3.2 *μ*g/mL against *B. subtilis*, and 3.2 *μ*g/mL against *Micrococcus luteus* (Kozakai et al., 2020). Despite their potential, the low yield and difficulties in synthesis have hindered their pharmaceutical commercialization (Mazo et al., 2023).

In our continuing efforts to explore novel bioactive metabolites from unique habitat-derived microbes (Chang et al., 2022a; Luo et al., 2022a; Chang et al., 2022b; Luo et al., 2022b), two lipolanthines (**1** and **2**) were produced by *Nocardia* sp. XZ19_369, which was isolated from the nodules of sea buckthorn collected in Tibet, China. The whole structures of **1** and **2** were fully elucidated by the extensive analysis of 1D and 2D NMR data, advanced Marfey’s method, and NMR calculations. These compounds represent the first lipolanthines with determined absolute configurations. Herein, the isolation, structural elucidation, and bioactivities of compounds **1** and **2** are described.

## 2 Experimental section

### 2.1 General experimental procedures

The optical rotations were obtained using a JASCO J-815 spectrometer (JASCO International Co. Ltd., Tokyo, Japan). 1D and 2D NMR spectra were measured using a Bruker AVⅢHD spectrometer (Bruker Co. Ltd., Bremen, Germany) at 600 MHz for ^1^H and 150 MHz for ^13^C in DMSO-*d*_6_ (*δ*_H_ 2.49 and *δ*_C_ 34.9). High-resolution mass data and advanced Marfey’s analysis were measured using a Waters Xevo G2-XS QTof mass spectrometer (Waters Co., Manchester, United Kingdom). Column chromatography was performed on macroporous adsorption resins (Diaion 4006; Nankai Chemical Co. Ltd., China) and the CombiFlash^®^ Rf system fitted with an ODS flash column (RediSep Rf C_18_ flash column, 130 g). Semi-preparative HPLC was performed using an Agilent 1200 series liquid chromatography system (quaternary pump, autosampler, and diode array detector) using a Reprosil-Pur Basic-C_18_ column (5 *μ*m, 250 × 10 mm).

### 2.2 Bacterial materials

*Nocardia* sp. XZ19_369 was isolated from the nodules of sea buckthorn collected from Tibet, China. Their draft genomes were sequenced on an Illumina HiSeq platform (Illumina, San Diego, CA, United States), assembled using SPAdes V3.13.1, and submitted to the National Center for Biotechnology Information (NCBI) database (accession no. JACVED000000000 for XZ19_369). The phylogenetic tree (Supplementary Figure S1) was constructed using autoMLST (Alanjary et al., 2019).

### 2.3 Fermentation

*Nocardia* sp. XZ19_369 grown on the ISP2 medium (0.4% glucose, 0.4% yeast extract, 1% malt extract, and 2% agar) was inoculated into 100 mL of the ISP2 liquid medium contained in a 500-mL Erlenmeyer flask and cultured at 28°C for 2 days as the seed culture. For large-scale fermentation, 50 mL of the seed culture was inoculated into 10 Erlenmeyer flasks (5 L), each containing 1 L of the sterile YMS medium (0.4% yeast extract, 1% malt extract and 0.4% starch soluble), and then cultivated at 28°C for 10 days.

### 2.4 Isolation and purification

The culture broth (20 L) was centrifuged at 4,000 rpm for 30 min to obtain the mycelium and supernatant; the supernatant was analyzed by LC–MS using an ACQUITY UPLC^®^ CSH™ C_18_ column (Waters, 1.7 *μ*m, 2.1 × 100 mm, at 30°C) eluted with the gradient method (from 10% to 100% MeCN/H_2_O, containing 0.1% formic acid at a flow rate of 0.3 mL/min for 10 min) (Supplementary Figure S2) and chromatographed over a macroporous adsorption resin column using the EtOH/H_2_O gradient elution (0%, 30%, 50%, 80%, and 100%; each 5 L) to afford five corresponding fractions (Fr.1: 2350.1 mg, Fr.2: 1576.5 mg, Fr.3: 923.5 mg, Fr.4: 323.6 mg, and Fr.5: 375.2 mg).

Fr.3 (923.5 mg) was then fractionated using an ODS flash column eluted with a gradient MeCN−H_2_O solution (1–10 min, 5%→25% MeCN; 10–50 min, 25%→50% MeCN; 50–70 min, 50%→100% MeCN; 70–80 min, and 100% MeCN; 15 mL/min) to yield four fractions (Fr.3–1−Fr.3–4). Fr.3–3 was further purified by reversed-phase semipreparative HPLC (Reprosil-Pur Basic-C_18_ column; 5 *μ*m, 250 × 10 mm, 2.5 mL/min, and 28% MeCN−H_2_O in 0.1% trifluoroacetic acid) to yield **1** (5.2 mg) and **2** (3.0 mg).

### 2.5 Spectroscopic data on compounds

#### 2.5.1 Nocaviogua A (**1**)

White powder; ${\left[\alpha \right]}_{D}^{20}$ -2.00 (*c* 0.2, CH_3_OH); UV (DAD) 219 nm and 261 nm; ^1^H (600 MHz, DMSO-*d*_6_) and ^13^C NMR (150 MHz, DMSO-*d*_6_), as shown in Table 1; HRESIMS *m/z* [M + H]^+^ 918.4514 (calcd for C_41_H_64_N_11_O_11_S, 918.4507).

**TABLE 1:** ^1^H NMR (600 MHz) and ^13^C NMR (150 MHz) data for compounds **1**–**2** **in** **DMSO-** ***d***_***6***_.

		Nocaviogua A (1)		Nocaviogua B (2)	
No.		*δ*_C_, type	*δ*_H_, multi. (*J* in Hz)	*δ*_C_, type	*δ*_H_, multi. (*J* in Hz)
MGFA	1	165.1, C	—	165.5, C	—
	2	122.9, CH	5.99, d (15.6)	122.7, CH	5.98, d (15.0)
	3	139.6, CH	7.07, dd (11.4, 15.0)	139.9, CH	6.96, dd (11.4, 15.0)
	4	128.7, CH	6.19, dd (11.4, 15.0)	128.7, CH	6.20, dd (11.4, 15.0)
	5	141.4, CH	6.05, dt (7.2, 15.0)	141.6, CH	6.04, dt (6.6, 15.0)
	6	32.3, CH_2_	2.17, m	32.3, CH_2_	2.18, m
	7	31.4, CH_2_	2.07, m	31.4, CH_2_	2.07, dt (7.2, 13.2)
	8	129.3, CH	5.40, dd (5.4, 15.0)	129.4, CH	5.38, dd (5.4, 15.6)
	9	130.4, CH	5.40, dd (5.4, 15.0)	130.4, CH	5.42, dd (5.4, 15.6)
	10	31.5, CH_2_	1.96, m	31.6, CH_2_	1.95, dt (7.2, 12.6)
	11	26.0, CH_2_	1.31, m	26.0, CH_2_	1.30, m
	12	28.0, CH_2_	1.46, m	28.0, CH_2_	1.46, m
	13	40.8, CH_2_	3.08, m	40.8, CH_2_	3.08, m
	14	155.2, C	—	155.2, C	—
	15	28.0, CH_3_	2.72, d (5.4)	28.0, CH_3_	2.72, d (4.8)
	16	28.0, CH_3_	2.72, d (5.4)	28.0, CH_3_	2.72, d (4.8)
	13-NH	—	7.32, t (6.0)	—	7.33, m
	15-NH	—	7.41, q (5.4)	—	7.43, q (4.8)
	16-NH	—	7.41, q (5.4)	—	7.43, q (4.8)
Ala_1_	1	171.5, C	—	172.3, C	—
	2	48.6, CH	4.20, m	48.7, CH	4.24, m
	3	17.6, CH_3_	1.16, d (7.2)	17.3, CH_3_	1.20, d (7.2)
	2-NH	—	8.06, d (6.6)	—	8.29, d (6.6)
Avi_2_	1	171.2, C	—	171.6, C	—
	2	52.2, CH	4.17, m	49.7, CH	4.27, m
	3	34.7, CH_2_	3.01, t (13.8)	34.3, CH_2_	2.27, m
			2.11, dd (5.4, 13.8)		2.50, m
	2-NH	—	7.91, d (7.8)	—	8.15, d (8.4)
Val_3_	1	171.3, C	—	172.7, C	—
	2	53.4, CH	4.16, m	57.6, CH	4.06, dd (6.6, 8.4)
	3	28.8, CH	1.97, m	29.8, CH	2.06, m
	4	20.2, CH_3_	0.74, d (6.0)	18.3, CH_3_	0.78, d (7.2)
	5	17.8, CH_3_	0.60, d (6.6)	17.3, CH_3_	0.84, d (7.2)
	2-NH	—	7.49, m	—	7.66, d (8.4)
Ser_4_	1	170.3, C	—	166.4, C	—
	2	58.4, CH	3.84, m	55.0, CH	3.86, m
	3	61.4, CH_2_	3.50, m	59.8, CH_2_	3.69, m
					3.82, m
	3-OH	—	—	—	5.65, s
	2-NH	—	7.40, m	—	—
	2-NH_2_	—	—	—	8.11, d (5.4)
Avi_5_	1	169.1, C	—	169.8, C	—
	2	60.9, C	—	62.0, C	—
	3	41.0, CH_2_	3.86, d (10.2)	41.6, CH_2_	2.07, m
			2.74, d (10.2)		1.96, m
	2-NH	—	6.83, s	—	8.60, s
Asn_6_ or Asp_6_	1	170.3, C	—	171.4, C	—
	2	49.6, CH	4.84, dt (3.0, 7.2)	49.7, CH	4.71, m
	3	36.0, CH_2_	2.75, m	35.8, CH_2_	2.25, m
			2.88, dd (3.0, 16.2)		2.76, m
	4	172.4, C	—	171.54, C	—
	4-NH_2_	—	—	—	7.33, s
					6.89, s
	2-NH	—	8.50, d (7.2)	—	8.20, d (8.4)
Gly_7_	1	167.6, C	—	167.0, C	—
	2	43.6, CH_2_	3.49, m	43.0, CH_2_	3.53, m
			4.07, dd (6.0, 16.8)		3.94, m
	2-NH	—	8.82, t (6.0)	—	8.81, t (6.0)
Avi_8_	1	98.7, CH	5.28, d (7.2)	99.3, CH	5.40, d (7.2)
	2	132.8, CH	7.16, dd (7.2, 11.4)	132.8, CH	7.19, dd (7.2, 11.4)
	2-NH	—	8.95, d (11.4)	—	8.73, d (11.4)

#### 2.5.2 Nocaviogua B (**2**)

White powder; ${\left[\alpha \right]}_{D}^{20}$ -2.96 (*c* 0.3, CH_3_OH); UV (DAD) 219 nm and 261 nm; ^1^H (600 MHz, DMSO-*d*_6_) and ^13^C NMR (150 MHz, DMSO-*d*_6_), as shown in Table 1; HRESIMS *m/z* [M + H]^+^ 935.4766 (calcd for C_41_H_67_N_12_O_11_S, 935.4773).

### 2.6 Advanced Marfey’s method

Each compound **1**–**2** (50 *μ*g) was dissolved in a 100 *μ*L of 6 N HCl and heated at 110°C for 24 h. After heating, the hydrolysates were divided into two parts and dried under the N_2_ flow. The dried hydrolysates and each standard amino acid (*L*-Ala, *L*-Val, *L*-Ser, and *L*-Asp) were dissolved in 30 *μ*L of the 0.1 M NaHCO_3_ solution. To each reaction vial, 30 *μ*L of *L*-FDAA (*N*_α_-(2,4-dinitro-5-fluorophenyl)-*L*-alaninamide, 1% solution in acetone) was added and heated at 40°C for 1 h (Kiyonaga Fujii, 1997a; Kiyonaga Fujii, 1997b). After cooling at room temperature, 30 *μ*L of 0.1 M HCl was added to each vial. The mixtures were diluted with 500 *μ*L of MeOH. The other part of hydrolysates and standard amino acids were derived with *D*-FDAA in the same manner. The aforementioned derivatives were analyzed with LC–MS using an ACQUITY UPLC^®^ CSH™ C_18_ column (Waters, 1.7 *μ*m, 2.1 × 100 mm, at 30°C) eluted with the gradient method (from 15% to 60% MeCN/H_2_O with a 5% isocratic MeOH containing 1% formic acid at a flow rate of 0.3 mL/min for 30 min) and isocratic elution method (from 12% MeCN/H_2_O with a 5% isocratic MeOH containing 1% formic acid at a flow rate of 0.3 mL/min for 30 min). The configurations of amino acids were confirmed by comparison with the authentic standards. The *L*-FDAA/*D*-FDAA derivatized authentic amino acids provided retention times (*t*_R_, min): *L*-Ala-*L*-FDAA and *L*-Ala-*D*-FDAA (7.80 and 9.22 min, *m/z* 342), *L*-Val-*L*-FDAA and *L*-Val-*D*-FDAA (11.52 and 14.18 min, *m/z* 370), *L*-Asp-*L*-FDAA and *L*-Asp-*D*-FDAA (6.22 and 6.76 min, *m/z* 386), and *L*-Ser-*L*-FDAA and *L*-Ser-*D*-FDAA (9.99 and 11.32 min, *m/z* 358). The presence of Ala, Val, Asp (Asn), and Ser residues in **1** and **2** was also assigned to the *L*-configuration.

### 2.7 NMR calculations

In order to simplify the calculation, the bismethylated guanidino fatty acid (MGFA) was replaced with acetic acid, which was not impacted with the accuracy of the result (Daranas and Sarotti, 2021). Conformational analysis was performed using OpenBabel (O'Boyle et al., 2011) with a genetic algorithm at the MMFF94 force field, with energies within the 3.0 kcal/mol energy threshold (Spartan14. Wavefunction, 2019). The conformers were optimized using Gaussian 16 (Gaussian Inc.) (M. J. Frisch, 2010) at the M062X/6-311+G (d, p) level in vacuum. At the same level, the frequencies were calculated to provide the relative thermal free energies (ΔG), which are used to calculate the equilibrium populations. NMR chemical shift calculations for those optimized conformers within their Boltzmann distribution (>1%) were performed using the GIAO method at the mPW1PW91/6-311+G (d, p) level in DMSO with the PCM model. The calculated chemical shifts of conformers for **1**–**2** were averaged in terms of their relative Gibbs free energy and the Boltzmann distribution theory. Finally, the calculated NMR chemical shift values were averaged according to Boltzmann distribution for each conformer and fit to the experimental values by linear regression. DP4+ probability analysis was performed according to the reported methods (Grimblat et al., 2015).

### 2.8 Antibacterial assays

Antibacterial assays were conducted in flat bottom, sterile 96-well plates (Corning, America) in triplicate, using a broth microdilution protocol (Cockerill, 2012). *Candida albicans* (ATCC 10231), *Staphylococcus aureus* (ATCC 29213), *Enterococcus faecium* (ATCC 35667), *Candida tropicalis* (ATCC 1369), *Escherichia coli* (ATCC 25922), *Klebsiella pneumoniae* (ATCC 700603), and *Pseudomonas aeruginosa* (ATCC 27853) were used as test strains. Each bacterial culture (100 *μ*L) containing ca. 5 × 10^4^ CFU was added to each well of 96-well plates. Nocaviogua A and B (**1** and **2**) were dissolved in DMSO. A measure of 1 *μ*L of each work solution of compounds **1**–**2** and the corresponding positive drugs (1.28–0.00125 mg/mL) were added to each well and incubated at 30°C for 24 h for determining MIC values.

### 2.9 Cytotoxic activity assays

The cytotoxic effects of compounds **1**–**2** were evaluated against uveal melanoma cells 92-1 (UM92-1), non-small cell lung cancer (NCI-H2170), and three breast cancer cell lines (SK-BR-3, MDA-MB-231, and MDA-MB-453) by the 3-(4, 5-dimethyl-2-thiazolyl)-2, 5-diphenyl-2H tetrazolium bromide (MTT) method. After cells (ca. 3×10^3^ cells/200 *μ*L/well) were seeded in a 96-well plate and cultured in a 5% CO_2_ incubator at 37°C for 24 h, **1** and **2** were added to each well for 48 h incubation. Then, the medium was removed, and the MTT solution was subsequently added to each well and maintained for 4 h. After removing the supernatant, 150 *μ*L DMSO was added to dissolve purple crystals. Ultimately, the absorbance value was read at 570 nm using a microplate reader (Elx800, BioTek Instruments, Inc., United States). The assays were performed four times, and bleomycin was used as the positive control.

## 3 Results and discussions

### 3.1 Structure elucidation

**FIGURE 1:**
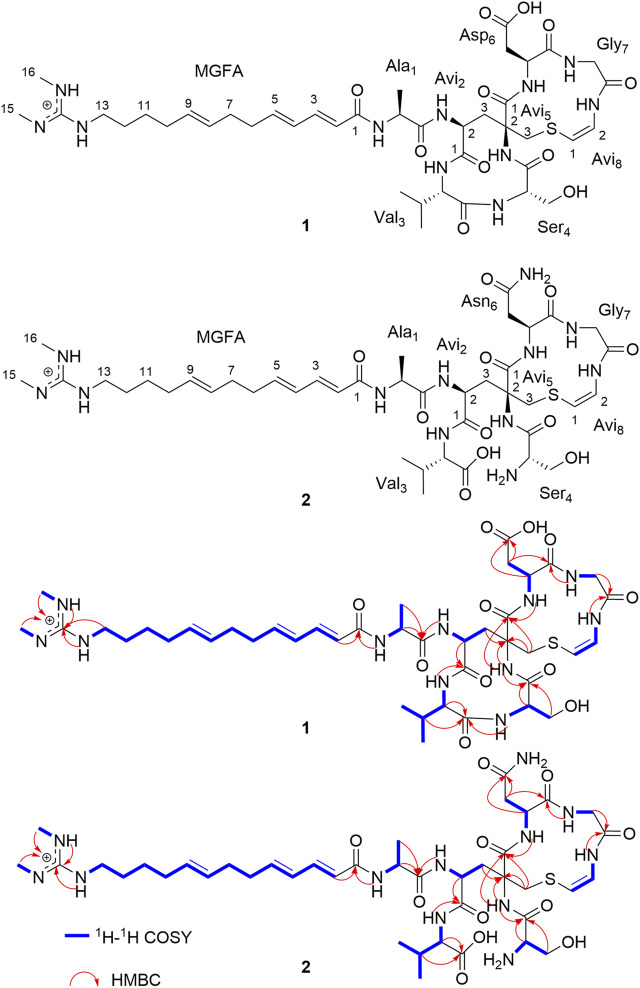
Key 2D NMR correlations of nocaviogua A (**1**) and nocaviogua B (**2**).

Nocaviogua A (**1**) was purified as white amorphous powder. HRESIMS of **1** disclosed a molecular formula of C_41_H_64_N_11_O_11_S using a quasi-molecular ion at *m/z* 918.4514 {[M + H]^+^, calcd 918.4507, with 16 double-bond equivalents (DBEs)}. The ^1^H NMR and ^13^C NMR data (Table 1) recorded in DMSO-*d*_6_ revealed nine amide and/or ester carbonyl carbons (*δ*_C_ 165.1–172.4), nine alkenyl carbons (*δ*_C_ 98.7–155.2 and *δ*_H_ 7.16–5.28), and eleven NH (*δ*_H_ 6.83–8.95), accounting for 14 DBEs and requiring that **1** incorporates two rings. The existence of one alanine (Ala) moiety in **1** was confirmed due to the presence of the spin system NH-CH-CH_3_ revealed by the ^1^H-^1^H COSY sequential correlations, together with the HMBC correlations from Ala_1_-2 (*δ*_H_ 4.20) and Ala_1_-3 (*δ*_H_ 1.16) to Ala_1_-CO (*δ*_C_ 171.5). One valine (Val) unit in **1** was inferred by the presence of a spin system of -NH-CH-CH(CH_3_)_2_, which is indicated by the ^1^H-^1^H COSY correlations, and then confirmed by the HMBC correlations from Val_3_-2 (*δ*_H_ 4.16) and Val_3_-3 (*δ*_H_ 1.97) to Val_3_-CO (*δ*_C_ 171.3). The existence of one serine (Ser) residue was identified based on the spin system of -NH-CH-CH_2_ deduced from ^1^H-^1^H COSY correlations, as well as the key HMBC correlations from Ser_4_-2 (*δ*_H_ 3.84) and Ser_4_-3 (*δ*_H_ 3.50) to Ser_4_-CO (*δ*_C_ 170.3) (Figure 1). One asparagine (Asn) or aspartic acid (Asp) moiety was inferred by the presence of the spin system of -NH-CH-CH_2_, which is indicated by the ^1^H-^1^H COSY correlations, and then confirmed by the HMBC correlations from Asp_6_/Asn_6_-3 (*δ*_H_ 2.75, 2.88) to Asp_6_/Asn_6_-CO_1_ (*δ*_C_ 170.3) and Asp_6_/Asn_6_-CO_4_ (*δ*_C_ 172.4), and Asp_6_/Asn_6_-2 (*δ*_H_ 4.84) to Asp_6_/Asn_6_-CO_4_ (*δ*_C_ 172.4). One glycine (Gly) residue was discovered due to the presence of the spin system of -NH-CH_2_ revealed by the ^1^H-^1^H COSY correlation, together with the HMBC correlations from Gly_7_-2 (*δ*_H_ 3.49, 4.07) to Gly_7_-CO (*δ*_C_ 167.6). The existence of one avionin, which includes three amino acid residues, namely, Avi_1_, Avi_2_, and Avi_3_ moieties, can be revealed by the spin system of NH-CH-CH_2_ in Avi_2_ and NH-CH = CH in Avi_8_, together with the key HMBC correlations from Avi_2_-2 (*δ*_H_ 4.17) to Avi_2_-CO (*δ*_C_ 171.2), from Avi_5_-3 (*δ*_H_ 2.74, 3.86) to Avi_5_-CO (*δ*_C_ 169.1) and Avi_5_-C_2_ (*δ*_C_ 60.9), and from Avi_5_-NH (*δ*_H_ 6.83) to Avi_5_-CO (*δ*_C_ 169.1) and Avi_5_-C_2_ (*δ*_C_ 60.9). The double-bond geometry of the Avi_8_ moiety was established as *Z* based on the small coupling constant (7.2 Hz) and the ROESY correlation between Avi_8_-(CH)_1_ and Avi_8_-(CH)_2_. Additionally, an N-terminal MGFA was confirmed by the spin system of -CH-CH-CH-CH-CH_2_-CH_2_-CH-CH-CH_2_-CH_2_-CH_2_-CH_2_-NH and two -NH-CH_3_ units, and key HMBC correlations from MGFA-2 (*δ*_H_ 5.99) to MGFA-CO (*δ*_C_ 165.1), and from MGFA-15 (*δ*_H_ 2.72), MGFA-16 (*δ*_H_ 2.72), and MGFA-13-NH (*δ*_H_ 7.32) to MGFA-C_14_ (*δ*_C_ 155.2). The alkenyls in MGFA were all *E* arrangements based on the large coupling constant (equal or greater than 15.0 Hz).

The connection sequence of these nine groups was established by HMBC correlations from Ala_1_-NH (*δ*_H_ 8.06) to MGFA-CO (*δ*_C_ 165.1), from Avi_2_-NH (*δ*_H_ 7.91) to Ala_1_-CO (*δ*_C_ 171.5), from Val_3_-NH (*δ*_H_ 7.49) to Avi_2_-CO (*δ*_C_ 171.2), from Ser_4_-NH (*δ*_H_ 7.40) to Val_3_-CO (*δ*_C_ 171.3), from Avi_5_-NH (*δ*_H_ 6.83) to Ser_4_-CO (*δ*_C_ 170.3), from Asp_6_/Asn_6_-NH (*δ*_H_ 8.50) to Avi_5_-CO (*δ*_C_ 169.1), from Gly_7_-NH (*δ*_H_ 8.82) to Asp_6_/Asn_6_-CO_1_ (*δ*_C_ 170.3), and from Avi_8_-NH (*δ*_H_ 8.95) to Gly_7_-CO (*δ*_C_ 167.6), suggesting that nine units were connected to yield a sequence of MGFA-Ala_1_-Avi_2_-Val_3_-Ser_4_-Avi_5_-Asp_6_/Asn_6_-Gly_7_-Avi_8_. The two DBEs were accounted for realizing the dicyclic structure of **1**. The key HMBC correlations from Avi_2_-3 (*δ*_H_ 3.01, 2.11) to Avi_5_-2 (*δ*_C_ 60.9) established the connectivity of Avi_2_ and Avi_5_. Additionally, considering the chemical shift of Avi_8_-1 (*δ*_C_ 98.7) and Avi_5_-3 (*δ*_C_ 41.0), and the requirement of unsaturation, the connection of (Avi_5_-3)-S-(Avi_8_-1) was deduced as found in nocavionin and microvionin (Wiebach et al., 2018). Based on the combination of previous information and the molecular formula, one Asp unit was assigned in **1**. Thus, the planar structure of **1** was determined, as shown in Figure 1. Notably, **1** contains an odd number of nitrogen atoms and an even number of hydrogen atoms, which suggests that the MGFA unit with the positively charged functional group is found in goadvionins (Kozakai et al., 2020).

The configurations of the amino acid residues in **1** were further determined by the advanced Marfey’s method. The acid hydrolysates of **1** were derivatized with *L*-FDAA and *D*-FDAA. By comparing the retention times with standards (Supplementary Figures S5–S8; Supplementary Table S1), the absolute configurations of Ala_1_, Val_3_, Ser_4_, and Asp_6_ were all established as *S*-configurations, leaving two chiralities in Avi_2_ and Avi_5_ residues to be clarified.

**FIGURE 2:**
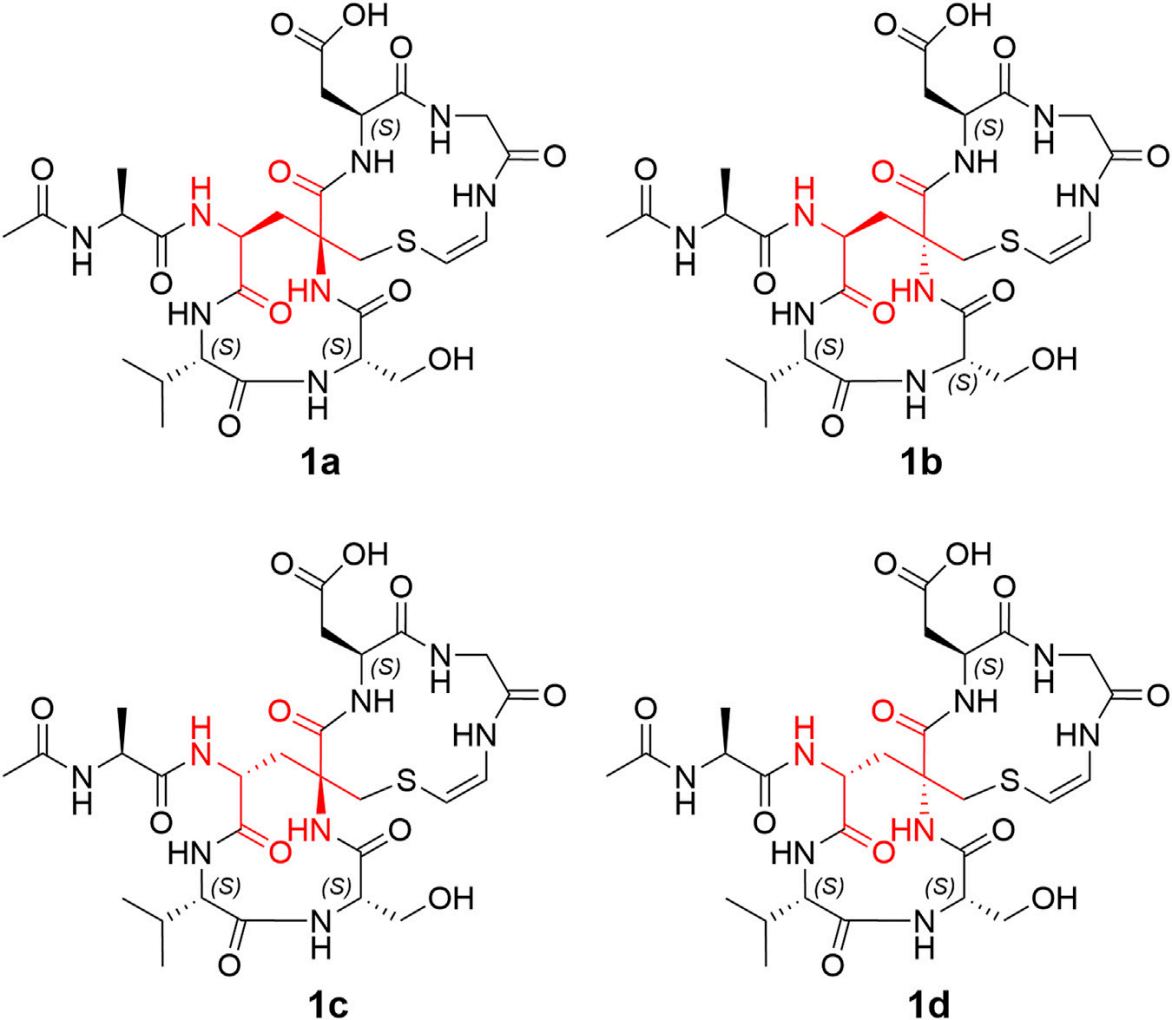
Four possible diastereomers (**1a**–**1d**).

**FIGURE 3:**
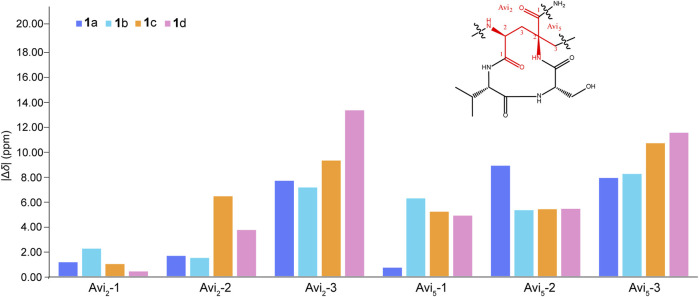
Corrected absolute errors of ^13^C chemical shifts in the Avi_2_ and Avi_5_ units calculated for **1a**–**1d**.

The remaining two configurations in Avi_2_ and Avi_5_ are proving to be a challenge to resolve. However, unlike the NMR spectra of nocavionin and microvionin in D_2_O and H_2_O, respectively, which showed the presence of conformer isomers (Wiebach et al., 2018), the NMR spectra of nocaviogua A (**1**) in DMSO-*d*_6_ did not exhibit any obvious signals indicating different conformers. This allows us to use the GIAO NMR calculation to determine their stereochemistry. In order to solve the structure of complex, large, and highly flexible molecules using readily available computational resources, the MGFA was replaced with acetic acid and the four possible diastereomers **1a**–**1d** (Figure 2) were evaluated. Conformational searches were performed at OpenBabel with the MMFF94 force field and an energy cutoff value of 3.00 kcal/mol. Subsequently, the obtained conformers were optimized by the DFT method at the M062X/6-311+G (d, p) level in vacuum, and the NMR chemical shifts were calculated at the PCM/mPW1PW91/6-311+G (d, p) level. Accordingly, the calculated carbon chemical shifts for **1a** showed the highest similarity toward the experimental values with the smallest corrected mean absolute error (CMAE, **1a**: 1.5113 vs. **1b**–**1d**: 2.0405, 1.9820, and 1.8977 in ^13^C NMR data and **1a**: 0.1397 vs. **1b**–**1d**: 0.2068, 0.1813, and 0.2073 in ^1^H NMR data, respectively) values (Supplementary Tables S2–S3), particularly for Avi_2_ and Avi_5_ moieties (Figure 3). In addition, DP4+ analysis based on NMR data provided 100% probability for the isomer **1a** (Supplementary Table S4), indicative of the *S*, *S* configuration for Avi_2_ and Avi_5_ moieties in **1**. Thus, the whole structure of compound **1** was fully determined and designated as nocaviogua A (**1**).

Nocaviogua B (**2**), white powder, was determined as C_41_H_66_N_12_O_11_S, based on the HRESIMS peak at *m/z* 935.4766 [M + H]^+^ (calcd for C_41_H_67_N_12_O_11_S, 935.4773), implying 15 DBEs. The ^1^H NMR and ^13^C NMR data (Table 1) for **2** that were recorded in DMSO-*d*_*6*_ revealed nine amide and/or ester carbonyl carbons (*δ*_C_ 165.5–172.7) and nine alkenyl carbons (*δ*_C_ 99.3–158.3 and *δ*_H_ 7.19–5.38), accounting for 14 DBEs and requiring only one ring incorporation into **2**. The ^1^H and ^13^C NMR (DMSO-*d*_6_) data for **2** were closely similar to those with **1** except for three signals of NH (Asn_6_-4-NH_2_: *δ*_H_ 7.33 and 6.89 and Ser_4_-NH_2_: *δ*_H_ 8.11) in **2,** which is more than **1**, indicating that the amide bond between Val_3_ and Ser_4_ was opened, and the Asp unit in **1** was replaced with the Asn residue in **2**. This hypothesis was further confirmed by HMBC and ^1^H-^1^H COSY correlations (Figure 1). Based on the coupling constant and the ROESY correlation, the geometry of the double bond in the Avi_8_ moiety was established as *Z* and the alkenyls in MGFA were all in the *E* arrangement, which were the same as in **1**. Considering the number of nitrogen atoms and hydrogen atoms, the MGFA unit in **2** was also with the positively charged functional group as in **1**.

The absolute configurations of Ala, Val, Ser, and Asn moieties were determined as *L* by the advanced Marfey’s method (Supplementary Figures S11–S14; Supplementary Table S1). The configurations in Avi_2_ and Avi_5_ units of compound **2** were also determined using GIAO NMR calculations by evaluating the four possible diastereomers **2a**–**2d** (Supplementary Figure S27). Consequently, the calculated carbon chemical shifts of **2a** showed the highest probability to the experimental values with the smallest CMAE (**2a**: 1.4284 vs. **2b**–**2d**: 1.6495, 1.8486, and 1.6907 in ^13^C NMR data and **2a**: 0.2186 vs. **2b**–**2d**: 0.2303, 0.2732, and 0.2248 in ^1^H NMR data, respectively) values (Supplementary Tables S13–S14). In addition, DP4+ analysis based on NMR data with 100% probability for the isomer **2a** (Supplementary Table S15), assigned Avi_2_ and Avi_5_ units as *S*, *S* configurations, respectively, are identical to those determined in compound **1**. Thus, the whole structure of compound **2** was fully determined and designated as nocaviogua B (**2**).

### 3.2 Antibacterial assays

The two compounds were evaluated for their antibacterial activity on *Candida albicans* (ATCC 10231), *Staphylococcus aureus* (ATCC 29213), *Enterococcus faecium* (ATCC 35667), *Candida tropicalis* (ATCC 1369), *Escherichia coli* (ATCC 25922), *Klebsiella pneumoniae* (ATCC 700603), and *Pseudomonas aeruginosa* (ATCC 27853) by MIC values. Regrettably, both compounds **1** and **2** showed no antibacterial effect at concentrations up to 128 *μ*g/mL.

### 3.3 Cytotoxic activity assays

**FIGURE 4:**
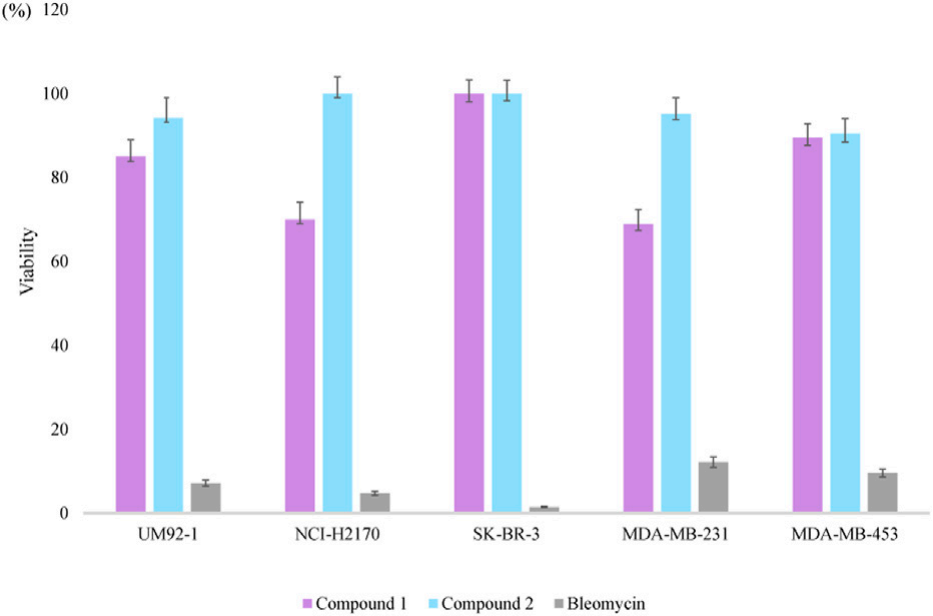
Cytotoxicity of **1** and **2,** and bleomycin against five types of cancer cell lines at 50 *μ*M.

The cytotoxicity of compounds **1**-**2** was assayed against uveal melanoma cells (UM92-1), non-small cell lung cancer (NCI-H2170), and three breast cancer cell lines (SK-BR-3, MDA-MB-231, and MDA-MB-453). Compound **1** exhibited weak cytotoxicity against UM92-1, NCI-H2170, and MDA-MB-231, exhibiting an inhibition rate of approximately 15%, 30.0%, and 31.1%, respectively, at a concentration of 50 *μ*M (Figure 4). Compound **2** shows no obvious cytotoxic activity at the same concentration.

## 4 Conclusion

Lipolanthines, featuring unique structures and physicochemical properties, are increasingly attracting wide interests as pharmaceutical leads (Grant-Mackie et al., 2021). In our research, two new lipolanthines, nocaviogua A (**1**) and B (**2**), were identified from mutualistic actinomycete *Nocardia* sp. XZ19_369 that was isolated from the nodules of sea buckthorn collected in Tibet. Their planar structures were elucidated via extensive 1D and 2D NMR, and HRMS data. The absolute configurations were fully elucidated by advanced Marfey’s analysis and GIAO NMR calculations.

Structurally, compounds **1** and **2** are analogs of microvionin, nocavionin, and goadvionins. Unlike their analogs, compounds **1** and **2** did not exhibit any antimicrobial activity against the seven tested pathogens at concentrations up to 128 *μ*g/mL. However, compound **1** did show weak cytotoxicity against cancer cell lines UM92-1, NCI-H2170, and MDA-MB-231, with inhibition rates of approximately 15%, 30.0%, and 31.1%, respectively, at a concentration of 50 *μ*M. On the other hand, compound **2**, the one-ring-opened derivative of compound **1**, did not show any obvious cytotoxic activity, suggesting that the bicycle scaffold in lipolanthines plays a crucial role in their cytotoxicity. To date, 12 lipolanthines have been discovered, but nocaviogua A (**1**) and B (**2**) were the first compounds in this family with fully determined configurations, which is of great importance for further stereoselective synthesis and bioactive studies.

## Data Availability

The datasets presented in this study can be found in online repositories. The names of the repository/repositories and accession number(s) can be found in the article/Supplementary Material.
