# Supplementary material for: Nocaviogua A and B: two lipolanthines from root-nodule-associated Nocardia sp
Source: Front Chem. 2023 Aug 3;11:1233938. doi: 10.3389/fchem.2023.1233938 (PMC10435860; doi:10.3389/fchem.2023.1233938)
Supplement: Supplementary file 1 [file DataSheet1.docx]

Nocaviogua A and B: two lipolanthines from root-nodule-associated Nocardia sp.

*Shanshan Chang^1^, Yajun Luo^2^, Ning He^1^, Xinyue Huang^1^, Mingxu Chen^1^, Lijie Yuan^2*^ and Yunying Xie^1*^*

^1^CAMS Key Laboratory of Synthetic Biology for Drug Innovation, Institute of Medicinal Biotechnology, Chinese Academy of Medical Sciences & Peking Union Medical College, Tiantan xili No.1, Beijing 100050, China

^2^Hebei Key Laboratory for Chronic Diseases, Tangshan Key Laboratory for Preclinical and Basic Research on Chronic Diseases, School of Basic Medical Sciences, North China University of Science and Technology, Tangshan, Hebei 063210, P.R. China

***Correspondence:**Yunying Xie
xieyy@imb.pumc.edu.cn (Yunying Xie)

Lijie Yuan

yuanlijie1970@163.com

**Supporting Information**

Content

[Fig. S1. Phylogenetic Tree of *Nocardia* sp. XZ19_369 and Its Homologs built though autoMLST using whole genomes and default paparmeters 5](#_Toc140152085)

[Fig. S2. LC-MS analysis of the fermentation broth of *Nocardia* sp. XZ19_369 6](#_Toc140152086)

[Fig. S3. The (+)-HRESIMS spectrum of compound **1**. 6](#_Toc140152087)

[Fig. S4. The UV spectrum of compound **1**. 7](#_Toc140152088)

[Fig. S5. The Marfey’s analysis of the acid hydrolysates of compound **1** and *L*-Ala. a) Extracted ion chromatogram at *m/z* 342 for *L*-FDAA derivatized hydrolyzates of **1**. b) Extracted ion chromatogram at *m/z* 342 for *D*-FDAA derivatized hydrolyzates of **1**. c) Extracted ion chromatogram at *m/z* 342 for *L*-FDAA derivatized of *L*-Ala. d) Extracted ion chromatogram at *m/z* 342 for *D*-FDAA derivatized of *L*-Ala. 8](#_Toc140152089)

[Fig. S6. The Marfey’s analysis of the acid hydrolysates of compound **1** and *L*-Val. a) Extracted ion chromatogram at *m/z* 370 for *L*-FDAA derivatized hydrolyzates of **1**. b) Extracted ion chromatogram at *m/z* 370 for *D*-FDAA derivatized hydrolyzates of **1**. c) Extracted ion chromatogram at *m/z* 370 for *L*-FDAA derivatized of *L*-Val. d) Extracted ion chromatogram at *m/z* 370 for *D*-FDAA derivatized of *L*-Val. 9](#_Toc140152090)

[. 9](#_Toc140152091)

[Fig. S7. The Marfey’s analysis of the acid hydrolysates of compound **1** and *L*-Asp. a) Extracted ion chromatogram at *m/z* 386 for *L*-FDAA derivatized hydrolyzates of **1**. b) Extracted ion chromatogram at *m/z* 386 for *D*-FDAA derivatized hydrolyzates of **1**. c) Extracted ion chromatogram at *m/z* 386 for *L*-FDAA derivatized of *L*-Asp. d) Extracted ion chromatogram at *m/z* 386 for *D*-FDAA derivatized of *L*-Asp. 10](#_Toc140152092)

[Fig. S8. The Marfey’s analysis of the acid hydrolysates of compound **1** and *L*-Ser. a) Extracted ion chromatogram at *m/z* 358 for *L*-FDAA derivatized hydrolyzates of **1**. b) Extracted ion chromatogram at *m/z* 358 for *D*-FDAA derivatized hydrolyzates of **1**. c) Extracted ion chromatogram at *m/z* 358 for *L*-FDAA derivatized of *L*-Ser. d) Extracted ion chromatogram at *m/z* 358 for *D*-FDAA derivatized of *L*-Ser. 11](#_Toc140152093)

[Fig. S9. The (+)-HRESIMS spectrum of compound **2**. 12](#_Toc140152094)

[Fig. S10. The UV spectrum of compound **2**. 12](#_Toc140152095)

[Fig. S11. The Marfey’s analysis of the acid hydrolysates of compound **2** and *L*-Ala. a) Extracted ion chromatogram at *m/z* 342 for *L*-FDAA derivatized hydrolyzates of **2**. b) Extracted ion chromatogram at *m/z* 342 for *D*-FDAA derivatized hydrolyzates of **2**. c) Extracted ion chromatogram at *m/z* 342 for *L*-FDAA derivatized of *L*-Ala. d) Extracted ion chromatogram at *m/z* 342 for *D*-FDAA derivatized of *L*-Ala. 13](#_Toc140152096)

[Fig. S12. The Marfey’s analysis of the acid hydrolysates of compound **2** and *L*-Val. a) Extracted ion chromatogram at *m/z* 370 for *L*-FDAA derivatized hydrolyzates of **2**. b) Extracted ion chromatogram at *m/z* 370 for *D*-FDAA derivatized hydrolyzates of **2**. c) Extracted ion chromatogram at *m/z* 370 for *L*-FDAA derivatized of *L*-Val. d) Extracted ion chromatogram at *m/z* 370 for *D*-FDAA derivatized of *L*-Val. 14](#_Toc140152097)

[Fig. S13. The Marfey’s analysis of the acid hydrolysates of compound **2** and *L*-Asn. a) Extracted ion chromatogram at *m/z* 386 for *L*-FDAA derivatized hydrolyzates of **2**. b) Extracted ion chromatogram at *m/z* 386 for *D*-FDAA derivatized hydrolyzates of **2**. c) Extracted ion chromatogram at *m/z* 386 for *L*-FDAA derivatized of *L*-Asn. d) Extracted ion chromatogram at *m/z* 386 for *D*-FDAA derivatized of *L*-Asn. 15](#_Toc140152098)

[Fig. S14. The Marfey’s analysis of the acid hydrolysates of compound **2** and *L*-Ser. a) Extracted ion chromatogram at *m/z* 358 for *L*-FDAA derivatized hydrolyzates of **2**. b) Extracted ion chromatogram at *m/z* 358 for *D*-FDAA derivatized hydrolyzates of **2**. c) Extracted ion chromatogram at *m/z* 358 for *L*-FDAA derivatized of *L*-Ser. d) Extracted ion chromatogram at *m/z* 358 for *D*-FDAA derivatized of *L*-Ser. 16](#_Toc140152099)

[Table S1. LC-MS retention times (*t*_R_, min) of the FDAA-derivatized amino acids from **1** and **2** in advanced Marfey’s analysis 16](#_Toc140152100)

[Fig. S15. The ^1^H NMR spectrum of compound **1** in DMSO-*d*_6_ (600 MHz). 17](#_Toc140152101)

[Fig. S16. The ^13^C NMR spectrum of compound **1** in DMSO-*d*_6_ (150 MHz). 17](#_Toc140152102)

[Fig. S17. The ^1^H-^1^H COSY spectrum of compound **1** in DMSO-*d*_6_ (600 MHz). 18](#_Toc140152103)

[Fig. S18. The HSQC spectrum of compound **1** in DMSO-*d*_6_ (600 MHz). 18](#_Toc140152104)

[Fig. S19. The HMBC spectrum of compound **1** in DMSO-*d*_6_ (600 MHz). 19](#_Toc140152105)

[Fig. S20. The ROESY spectrum of compound **1** in DMSO-*d*_6_ (600 MHz). 19](#_Toc140152106)

[Fig. S21. The ^1^H NMR spectrum of compound **2** in DMSO-*d*_6_ (600 MHz). 20](#_Toc140152107)

[Fig. S22. The ^13^C NMR spectrum of compound **2** in DMSO-*d*_6_ (150 MHz). 20](#_Toc140152108)

[Fig. S23. The ^1^H-^1^H COSY spectrum of compound **2** in DMSO-*d*_6_ (600 MHz). 21](#_Toc140152109)

[Fig. S24. The HSQC spectrum of compound **2** in DMSO-*d*_6_ (600 MHz). 21](#_Toc140152110)

[Fig. S25. The HMBC spectrum of compound **2** in DMSO-*d*_6_ (600 MHz). 22](#_Toc140152111)

[Fig. S26. The ROESY spectrum of compound **2** in DMSO-*d*_6_ (600 MHz). 22](#_Toc140152112)

[Table S2. Experimental and Calculated ^13^C NMR Chemical Shifts of **1**a–**1**d 23](#_Toc140152113)

[Table S3. Experimental and Calculated ^1^H NMR Chemical Shifts of **1**a–**1**d 24](#_Toc140152114)

[Table S4. DP4+ Probabilities Computed for **1**a-**1**d 25](#_Toc140152115)

[Table S5. Lowest-energy Conformers Optimized at the M062X/6-311+G (d, p) Level of **1a** with Relative Energies < 3.0 kcal/mol 26](#_Toc140152116)

[Table S6. Atomic Coordinates for the Lowest-energy Conformers of **1a** (**1a_C1**–**1a_C8**) 27](#_Toc140152117)

[Table S7. Lowest-energy Conformers Optimized at the M062X/6-311+G (d, p) Level of **1b** with Relative Energies < 3.0 kcal/mol 29](#_Toc140152118)

[Table S8. Atomic Coordinates for the Lowest-energy Conformers of **1b** (**1b_C1**–**1b_C3**) 29](#_Toc140152119)

[Table S9. Lowest-energy Conformers Optimized at the M062X/6-311+G (d, p) Level of **1c** with Relative Energies < 3.0 kcal/mol 31](#_Toc140152120)

[Table S10. Atomic Coordinates for the Lowest-energy Conformers of **1c** (**1c_C1**–**1c_C5**) 31](#_Toc140152121)

[Table S11. Lowest-energy Conformers Optimized at the M062X/6-311+G (d, p) Level of **1d** with Relative Energies < 3.0 kcal/mol 32](#_Toc140152122)

[Table S12. Atomic Coordinates for the Lowest-energy Conformers of **1d** (**1d_C1**–**1d_C8**) 33](#_Toc140152123)

[Fig. S27. The four possible diastereomers (**2a**, **2b**, **2c** and **2d**). 36](#_Toc140152124)

[Table S13. Experimental and Calculated ^13^C NMR Chemical Shifts of **2**a–**2**d 36](#_Toc140152125)

[Table S14. Experimental and Calculated ^1^H NMR Chemical Shifts of **2**a–**2**d 37](#_Toc140152126)

[Table S15. DP4+ Probabilities Computed for **2**a-**2**d 38](#_Toc140152127)

[Table S16. Lowest-energy Conformers Optimized at the M062X/6-311+G (d, p) Level of **2**a with Relative Energies < 3.0 kcal/mol 39](#_Toc140152128)

[Table S17. Atomic Coordinates for the Lowest-energy Conformers of **2a** (**2a_C1**–**2a_C6**) 39](#_Toc140152129)

[Table S18. Lowest-energy Conformers Optimized at the M062X/6-311+G (d, p) Level of **2**b with Relative Energies < 3.0 kcal/mol 42](#_Toc140152130)

[Table S19. Atomic Coordinates for the Lowest-energy Conformers of **2b** (**2b_C1**–**2b_C3**) 42](#_Toc140152131)

[Table S20. Lowest-energy Conformers Optimized at the M062X/6-311+G (d, p) Level of **2**c with Relative Energies < 3.0 kcal/mol 43](#_Toc140152132)

[Table S21. Atomic Coordinates for the Lowest-energy Conformers of **2c** (**2c_C1**–**2c_C3**) 43](#_Toc140152133)

[Table S22. Lowest-energy Conformers Optimized at the M062X/6-311+G (d, p) Level of **2d** with Relative Energies < 3.0 kcal/mol 45](#_Toc140152134)

[Table S23. Atomic Coordinates for the Lowest-energy Conformers of **2d** (**2d_C1**–**2d_C5**) 45](#_Toc140152135)


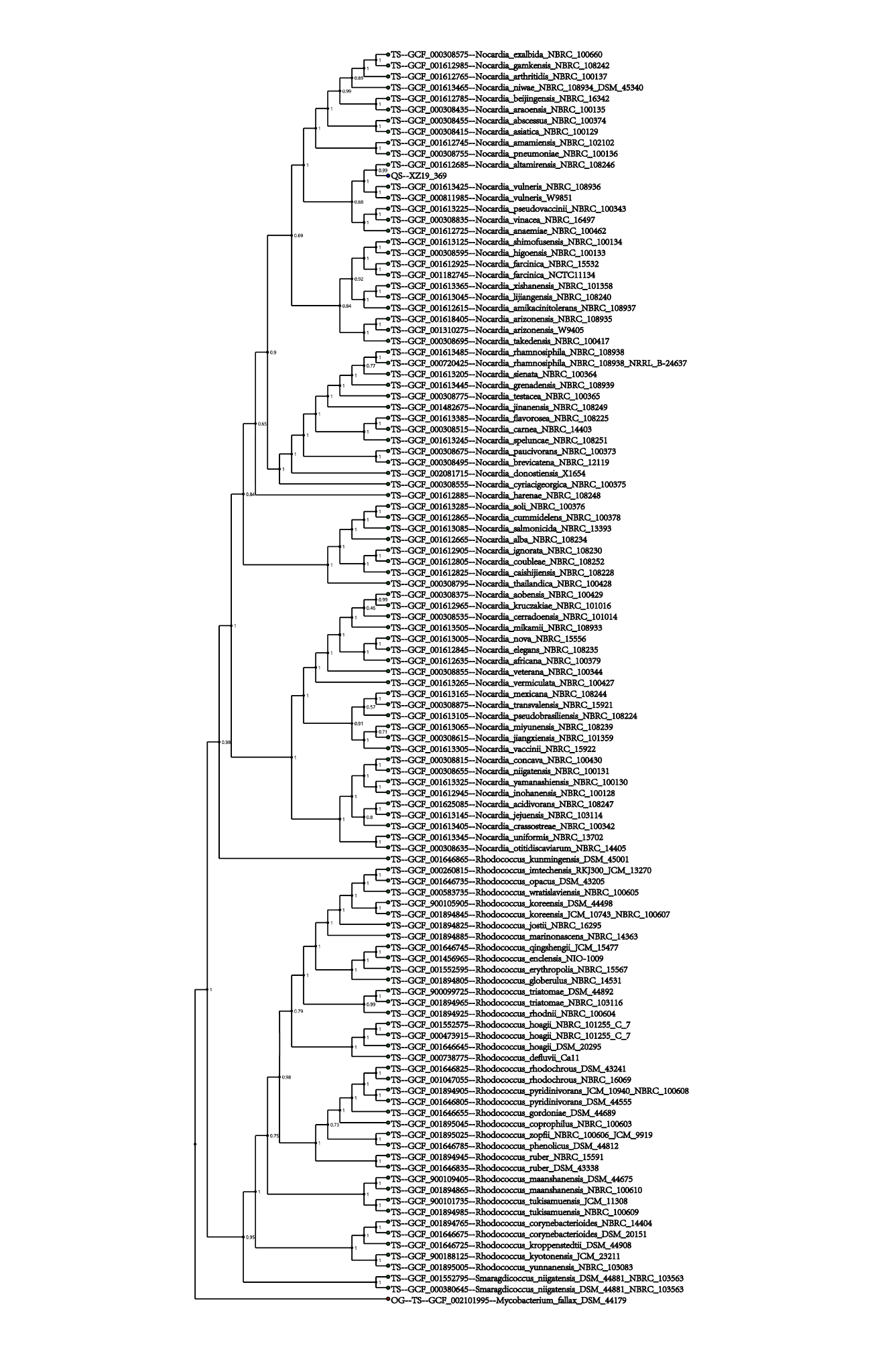


# Fig. S1. Phylogenetic Tree of *Nocardia* sp. XZ19_369 and Its Homologs built though autoMLST using whole genomes and default paparmeters


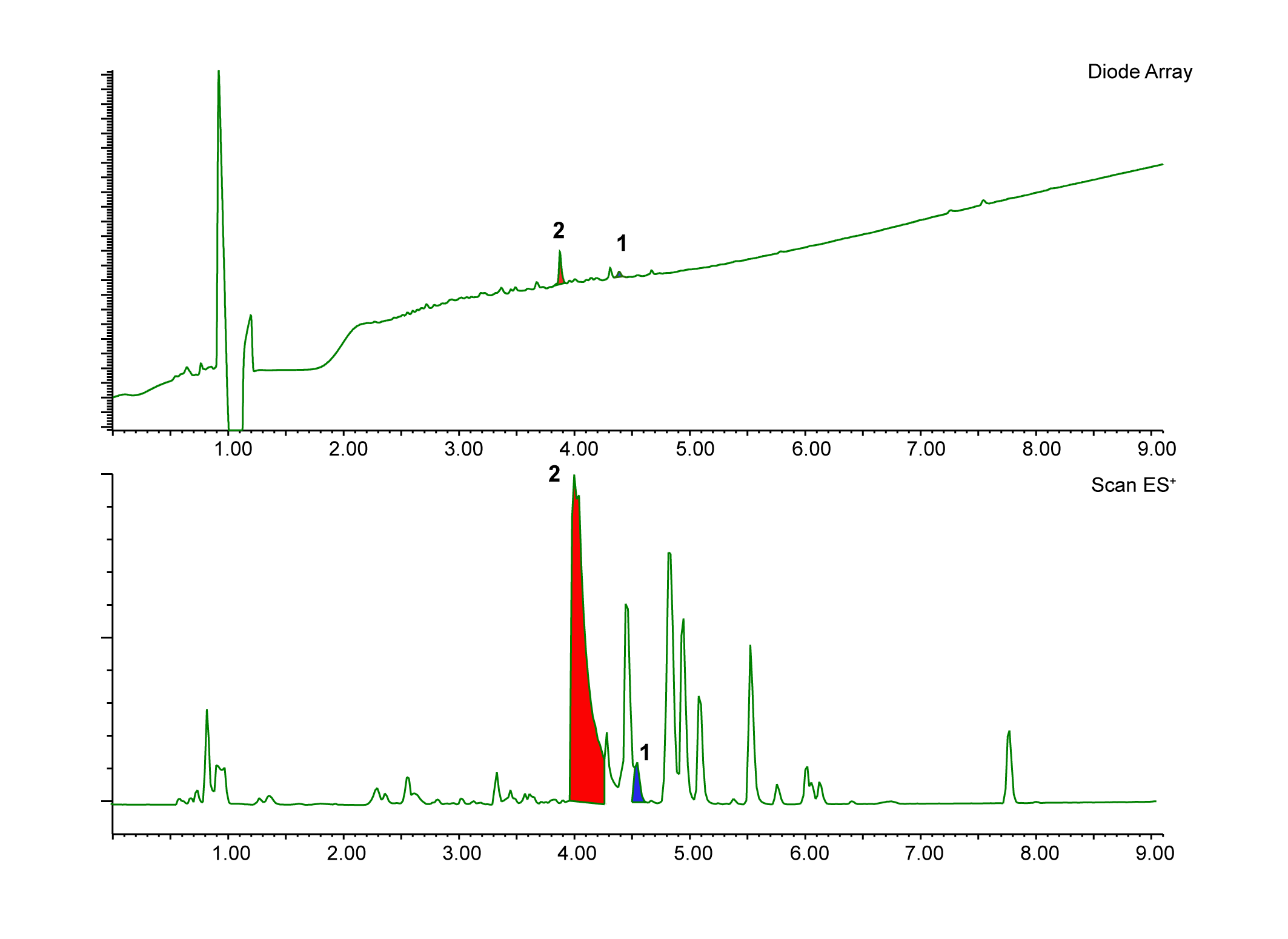


# Fig. S2. LC-MS analysis of the fermentation broth of *Nocardia* sp. XZ19_369


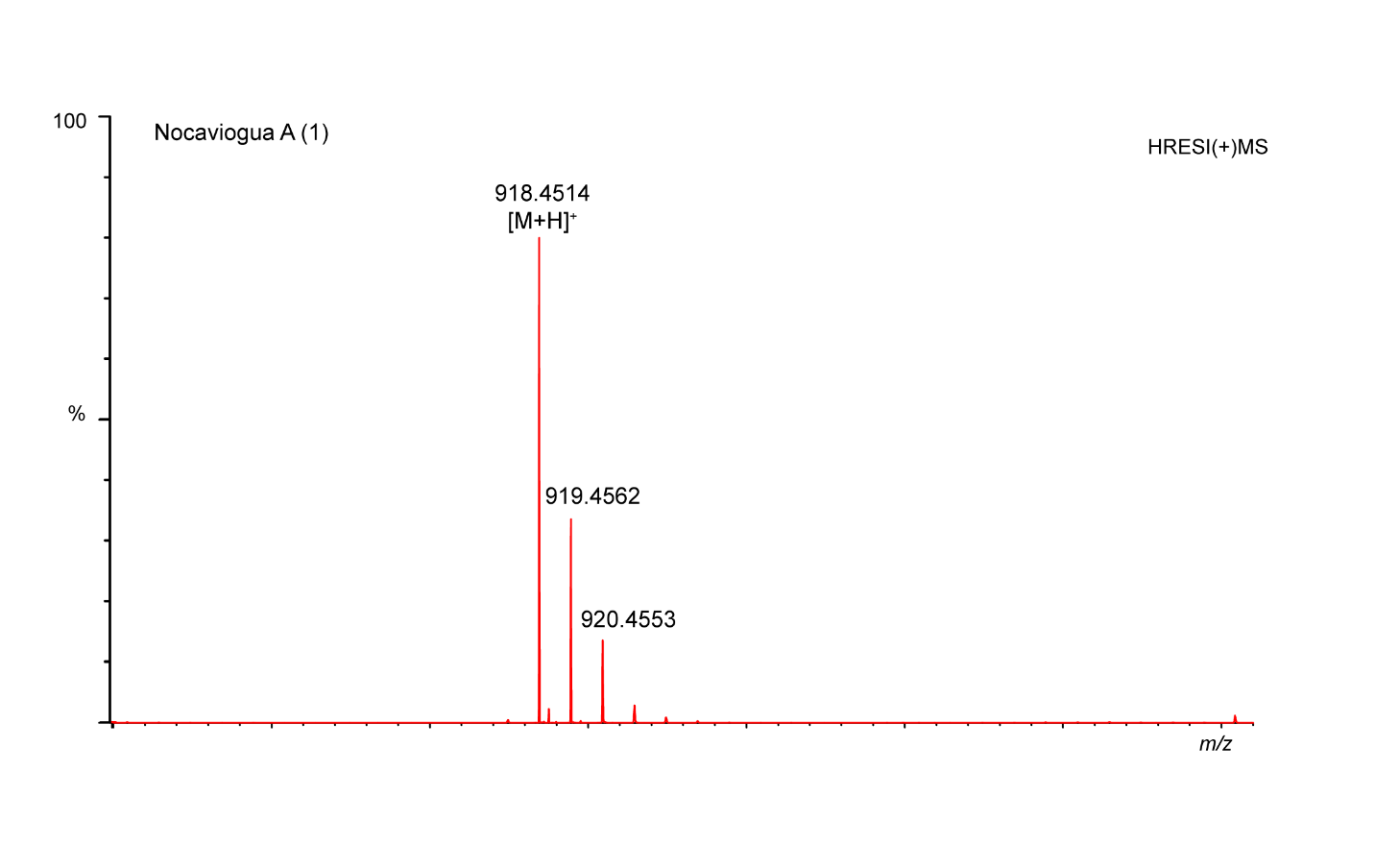


# Fig. S3. The (+)-HRESIMS spectrum of compound **1**.


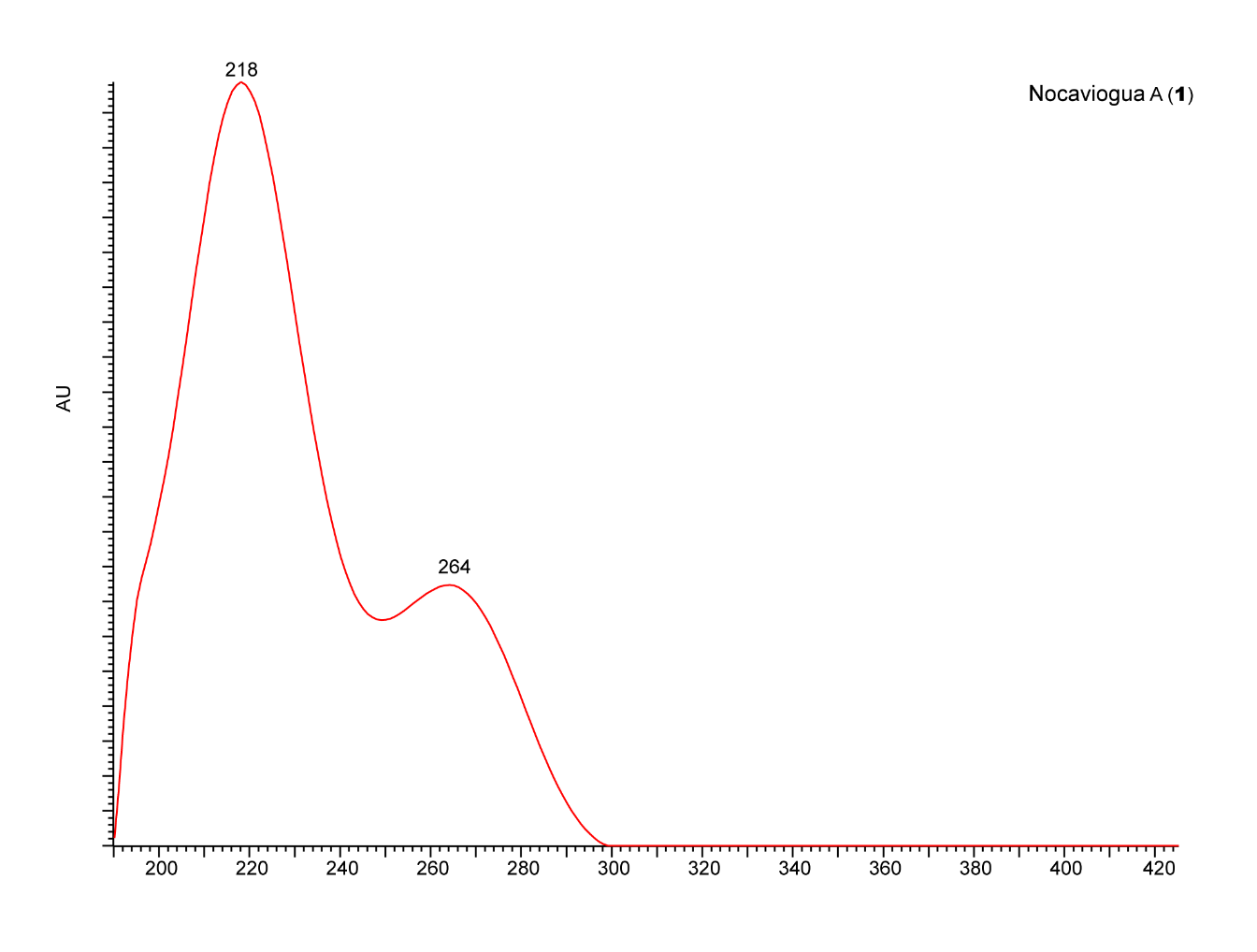


# Fig. S4. The UV spectrum of compound **1**.


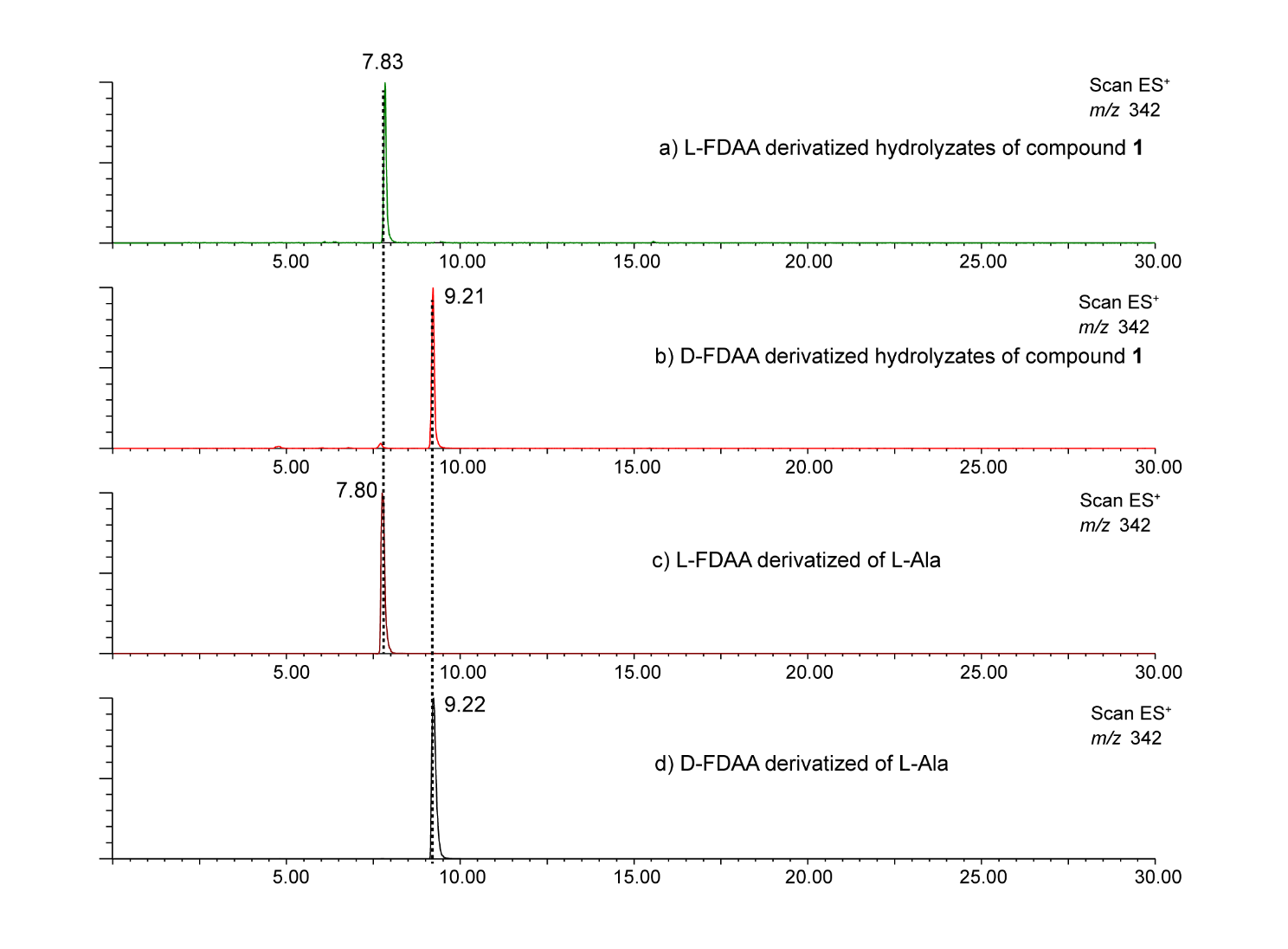


# Fig. S5. The Marfey’s analysis of the acid hydrolysates of compound **1** and *L*-Ala. a) Extracted ion chromatogram at *m/z* 342 for *L*-FDAA derivatized hydrolyzates of **1**. b) Extracted ion chromatogram at *m/z* 342 for *D*-FDAA derivatized hydrolyzates of **1**. c) Extracted ion chromatogram at *m/z* 342 for *L*-FDAA derivatized of *L*-Ala. d) Extracted ion chromatogram at *m/z* 342 for *D*-FDAA derivatized of *L*-Ala.


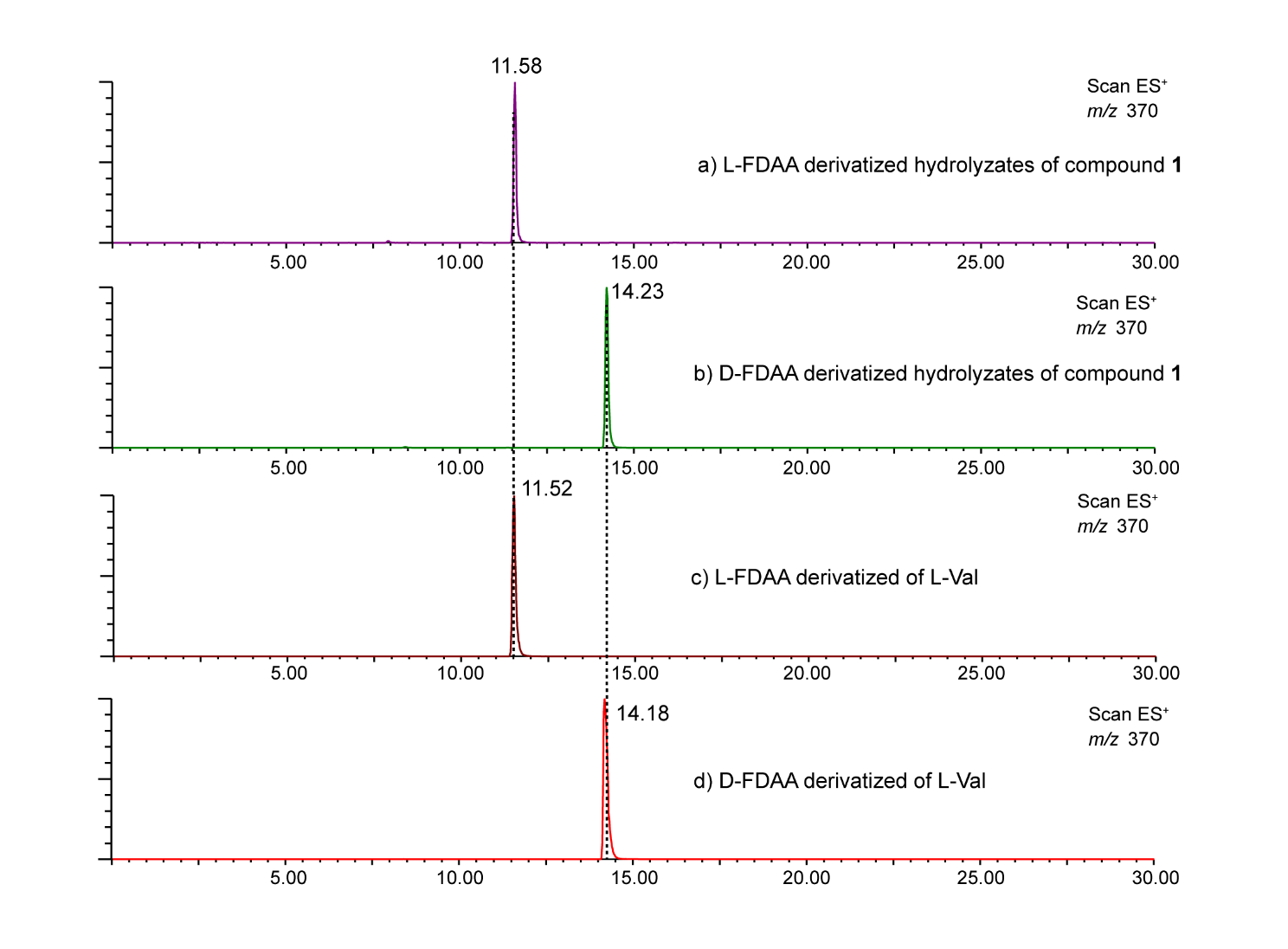


# Fig. S6. The Marfey’s analysis of the acid hydrolysates of compound **1** and *L*-Val. a) Extracted ion chromatogram at *m/z* 370 for *L*-FDAA derivatized hydrolyzates of **1**. b) Extracted ion chromatogram at *m/z* 370 for *D*-FDAA derivatized hydrolyzates of **1**. c) Extracted ion chromatogram at *m/z* 370 for *L*-FDAA derivatized of *L*-Val. d) Extracted ion chromatogram at *m/z* 370 for *D*-FDAA derivatized of *L*-Val.

.


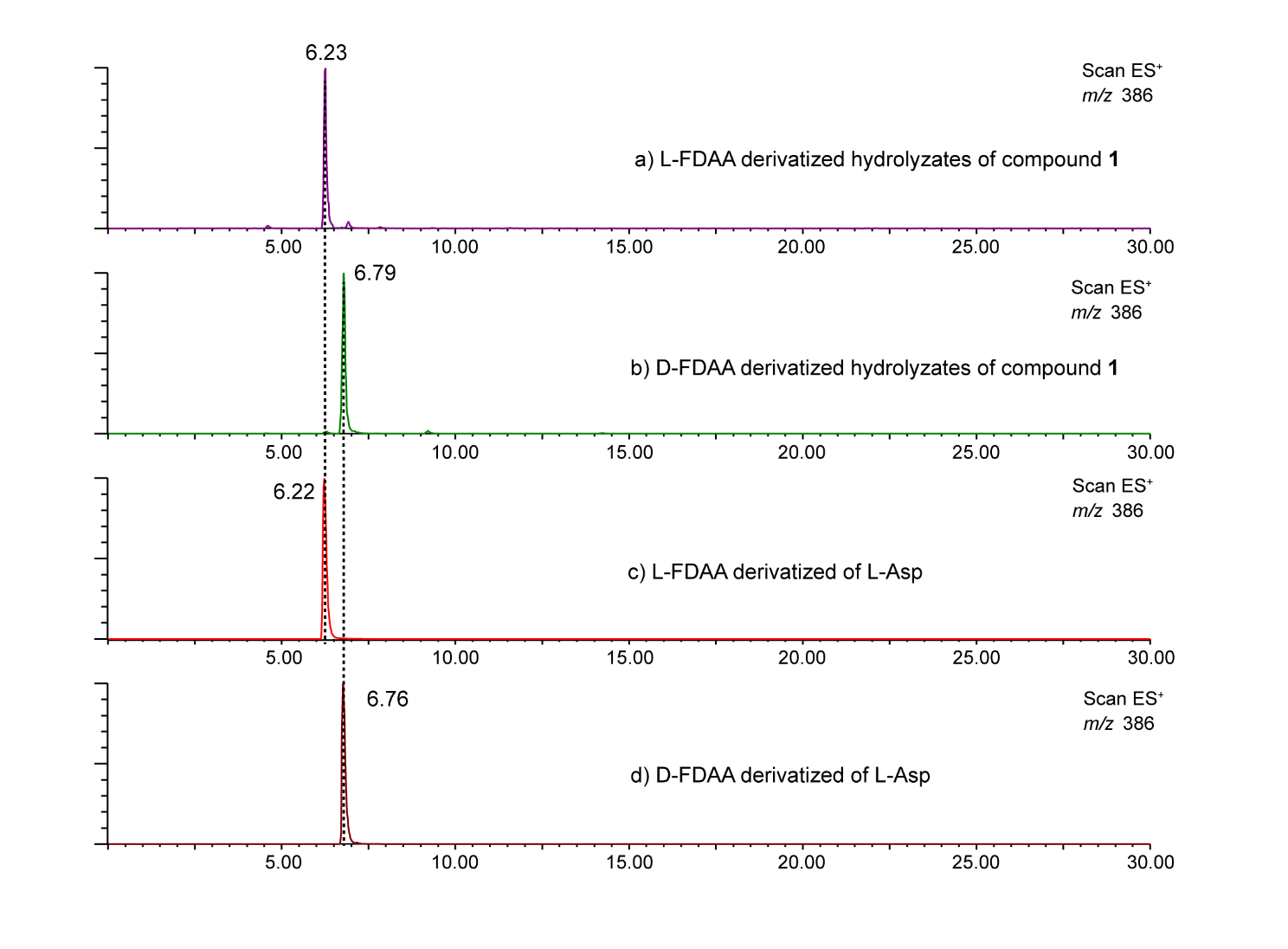


# Fig. S7. The Marfey’s analysis of the acid hydrolysates of compound **1** and *L*-Asp. a) Extracted ion chromatogram at *m/z* 386 for *L*-FDAA derivatized hydrolyzates of **1**. b) Extracted ion chromatogram at *m/z* 386 for *D*-FDAA derivatized hydrolyzates of **1**. c) Extracted ion chromatogram at *m/z* 386 for *L*-FDAA derivatized of *L*-Asp. d) Extracted ion chromatogram at *m/z* 386 for *D*-FDAA derivatized of *L*-Asp.


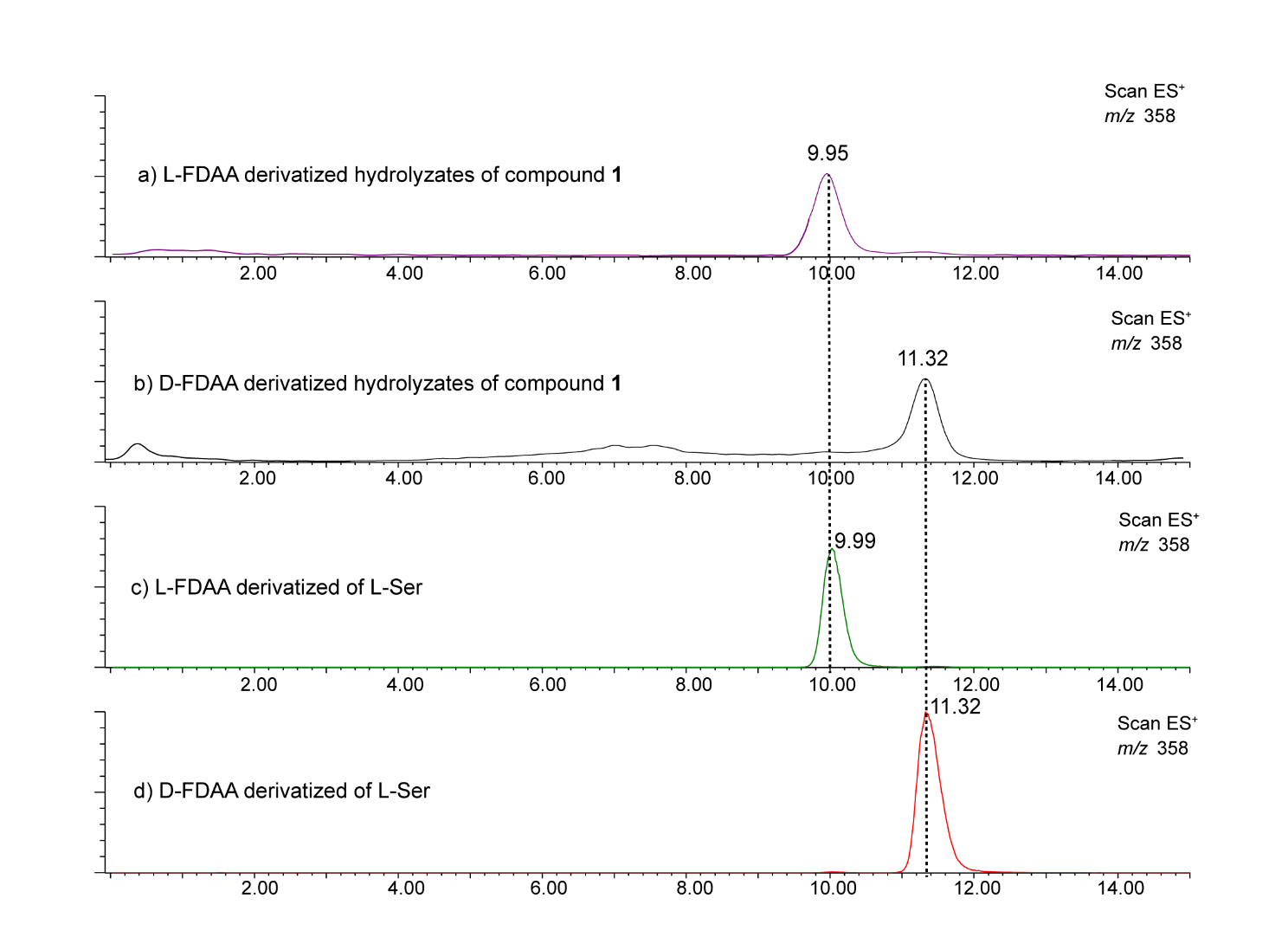


# Fig. S8. The Marfey’s analysis of the acid hydrolysates of compound **1** and *L*-Ser. a) Extracted ion chromatogram at *m/z* 358 for *L*-FDAA derivatized hydrolyzates of **1**. b) Extracted ion chromatogram at *m/z* 358 for *D*-FDAA derivatized hydrolyzates of **1**. c) Extracted ion chromatogram at *m/z* 358 for *L*-FDAA derivatized of *L*-Ser. d) Extracted ion chromatogram at *m/z* 358 for *D*-FDAA derivatized of *L*-Ser.


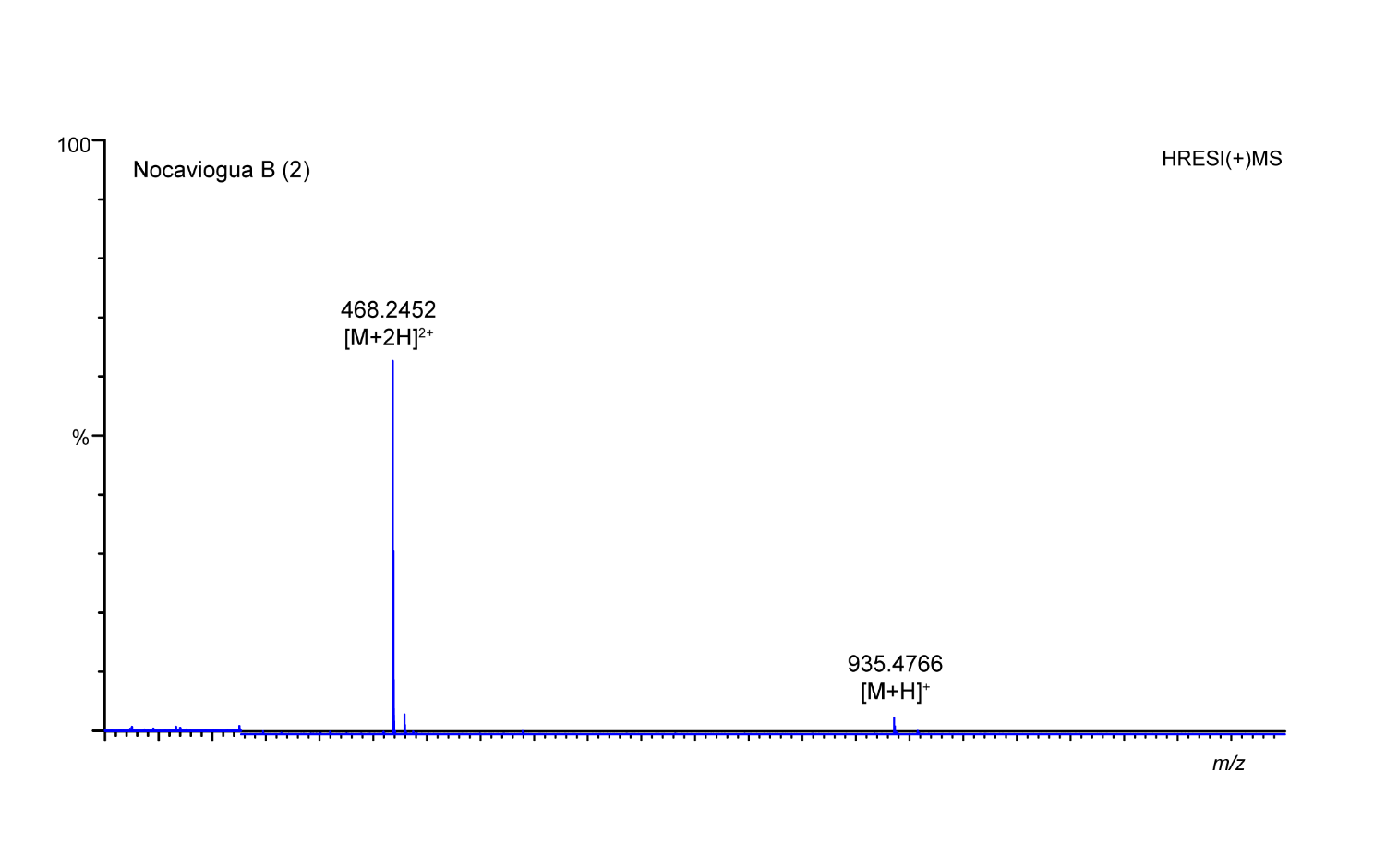


# Fig. S9. The (+)-HRESIMS spectrum of compound **2**.


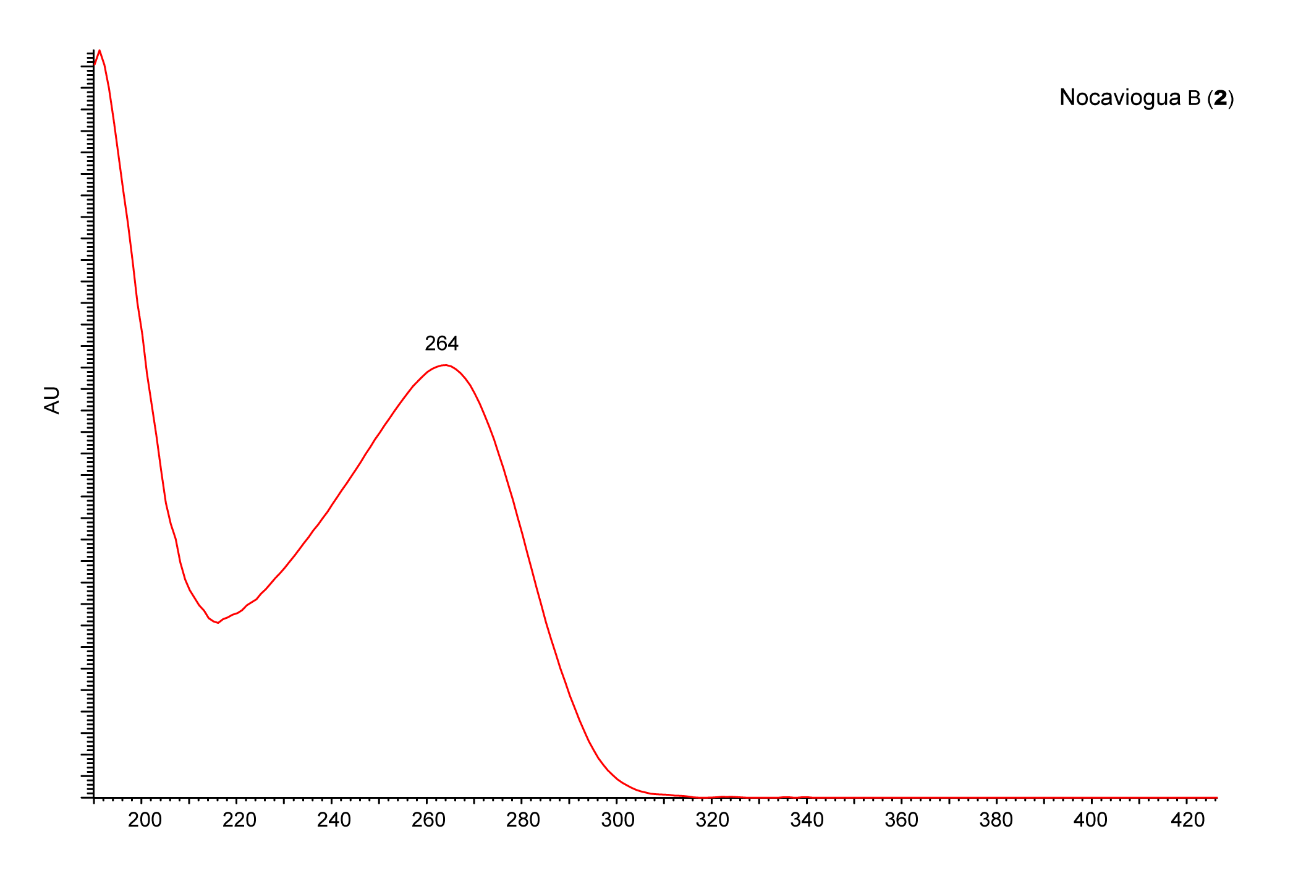


# Fig. S10. The UV spectrum of compound **2**.


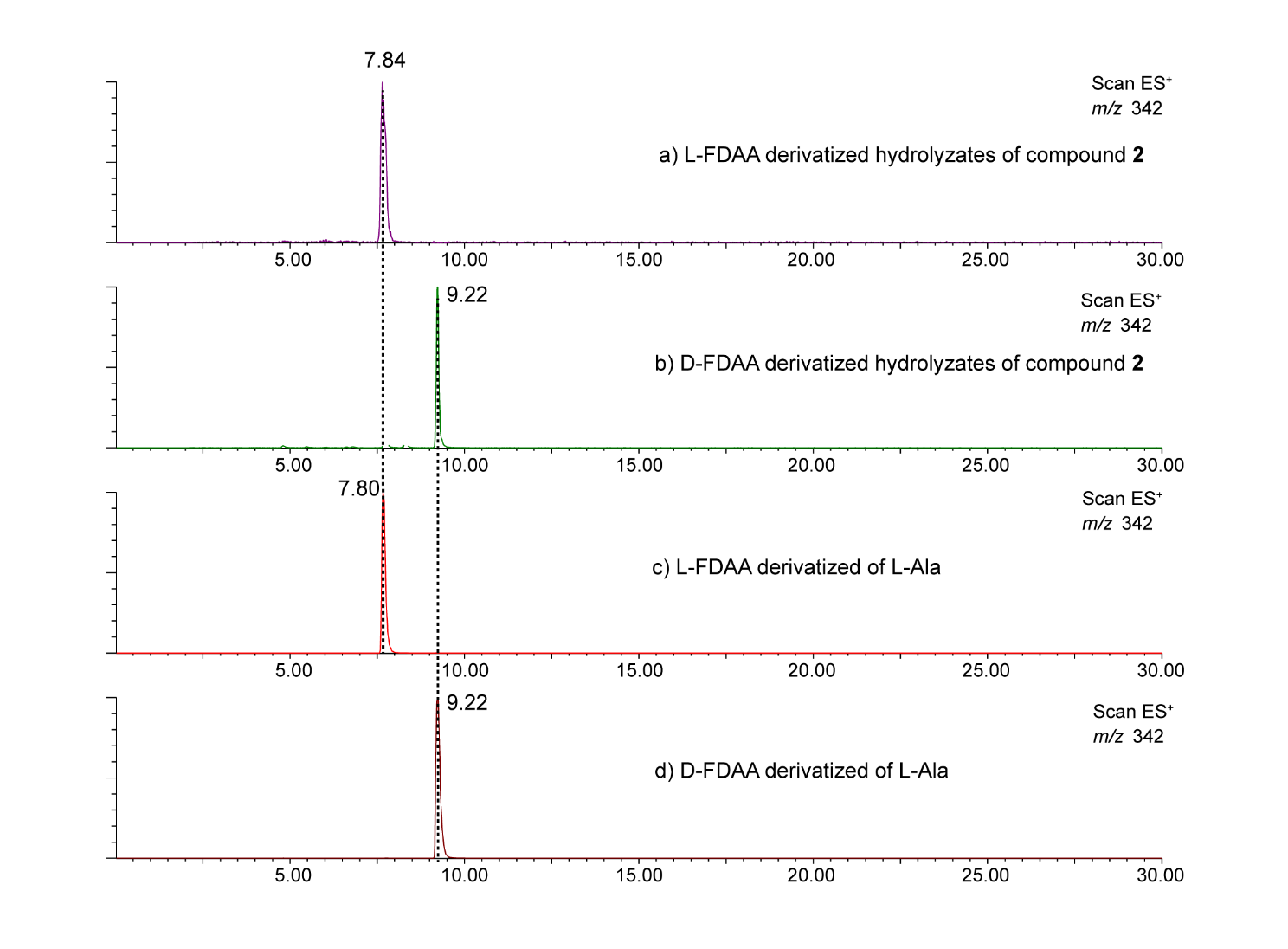


# Fig. S11. The Marfey’s analysis of the acid hydrolysates of compound **2** and *L*-Ala. a) Extracted ion chromatogram at *m/z* 342 for *L*-FDAA derivatized hydrolyzates of **2**. b) Extracted ion chromatogram at *m/z* 342 for *D*-FDAA derivatized hydrolyzates of **2**. c) Extracted ion chromatogram at *m/z* 342 for *L*-FDAA derivatized of *L*-Ala. d) Extracted ion chromatogram at *m/z* 342 for *D*-FDAA derivatized of *L*-Ala.


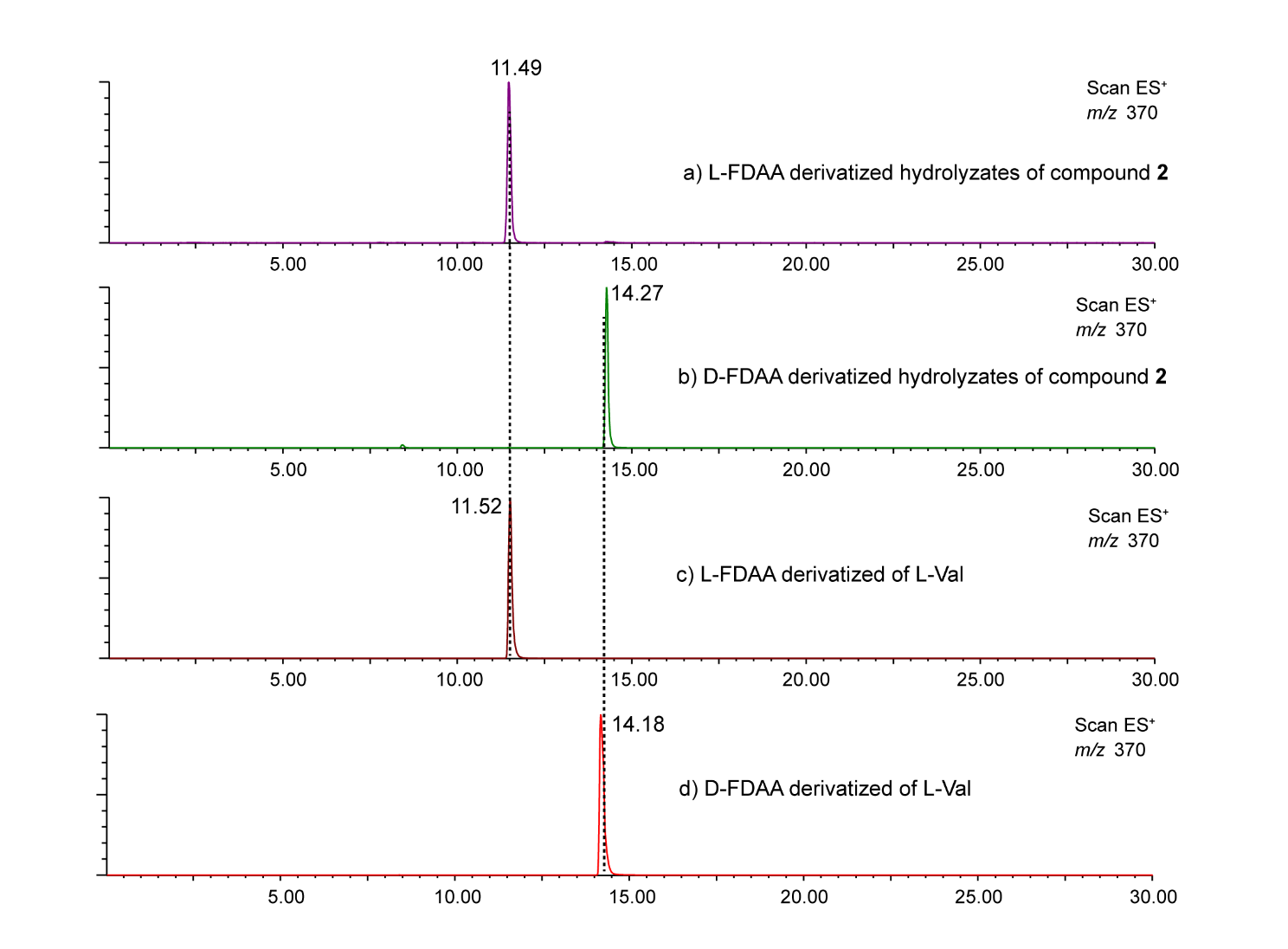


# Fig. S12. The Marfey’s analysis of the acid hydrolysates of compound **2** and *L*-Val. a) Extracted ion chromatogram at *m/z* 370 for *L*-FDAA derivatized hydrolyzates of **2**. b) Extracted ion chromatogram at *m/z* 370 for *D*-FDAA derivatized hydrolyzates of **2**. c) Extracted ion chromatogram at *m/z* 370 for *L*-FDAA derivatized of *L*-Val. d) Extracted ion chromatogram at *m/z* 370 for *D*-FDAA derivatized of *L*-Val.


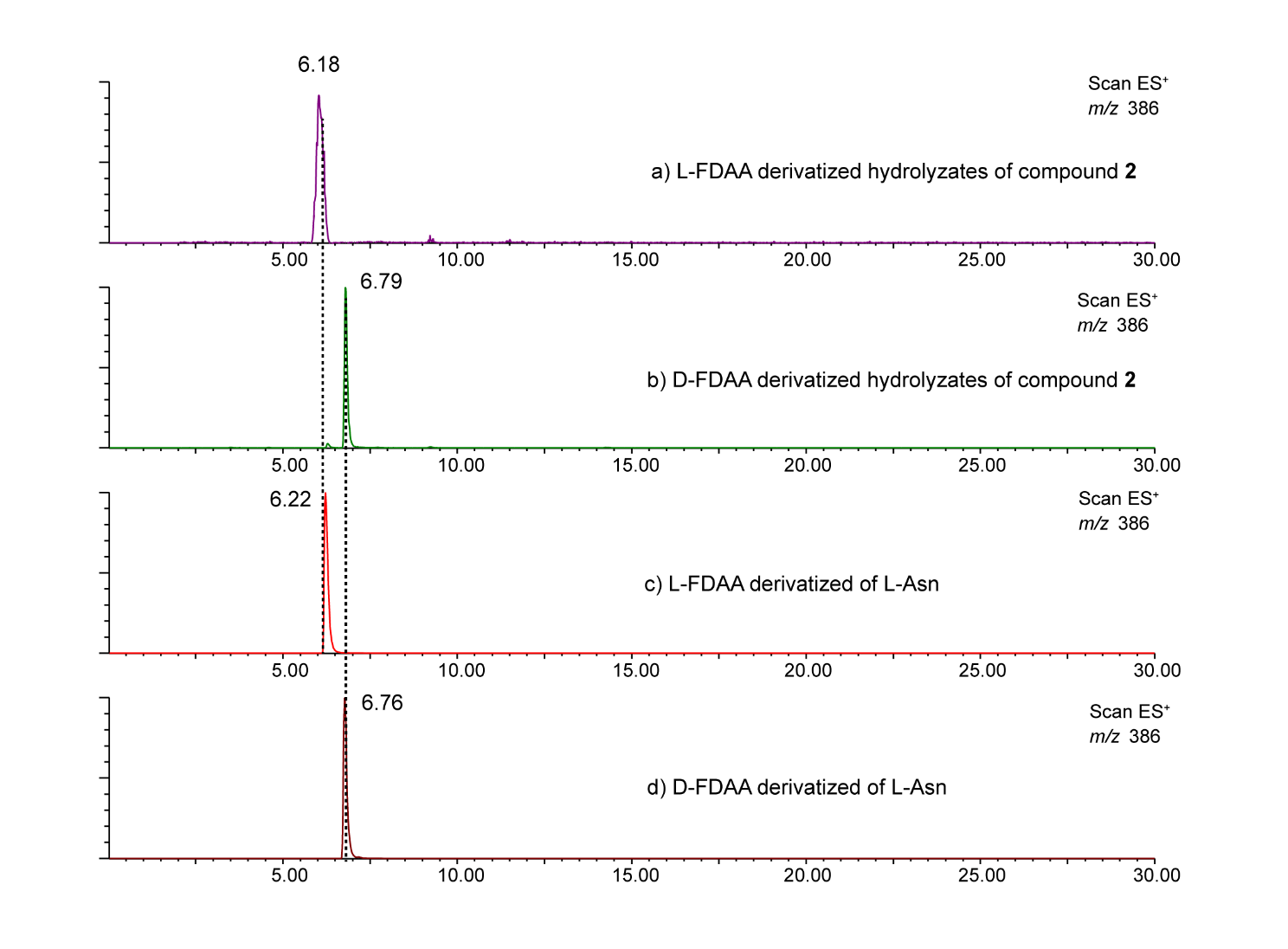


# Fig. S13. The Marfey’s analysis of the acid hydrolysates of compound **2** and *L*-Asn. a) Extracted ion chromatogram at *m/z* 386 for *L*-FDAA derivatized hydrolyzates of **2**. b) Extracted ion chromatogram at *m/z* 386 for *D*-FDAA derivatized hydrolyzates of **2**. c) Extracted ion chromatogram at *m/z* 386 for *L*-FDAA derivatized of *L*-Asn. d) Extracted ion chromatogram at *m/z* 386 for *D*-FDAA derivatized of *L*-Asn.


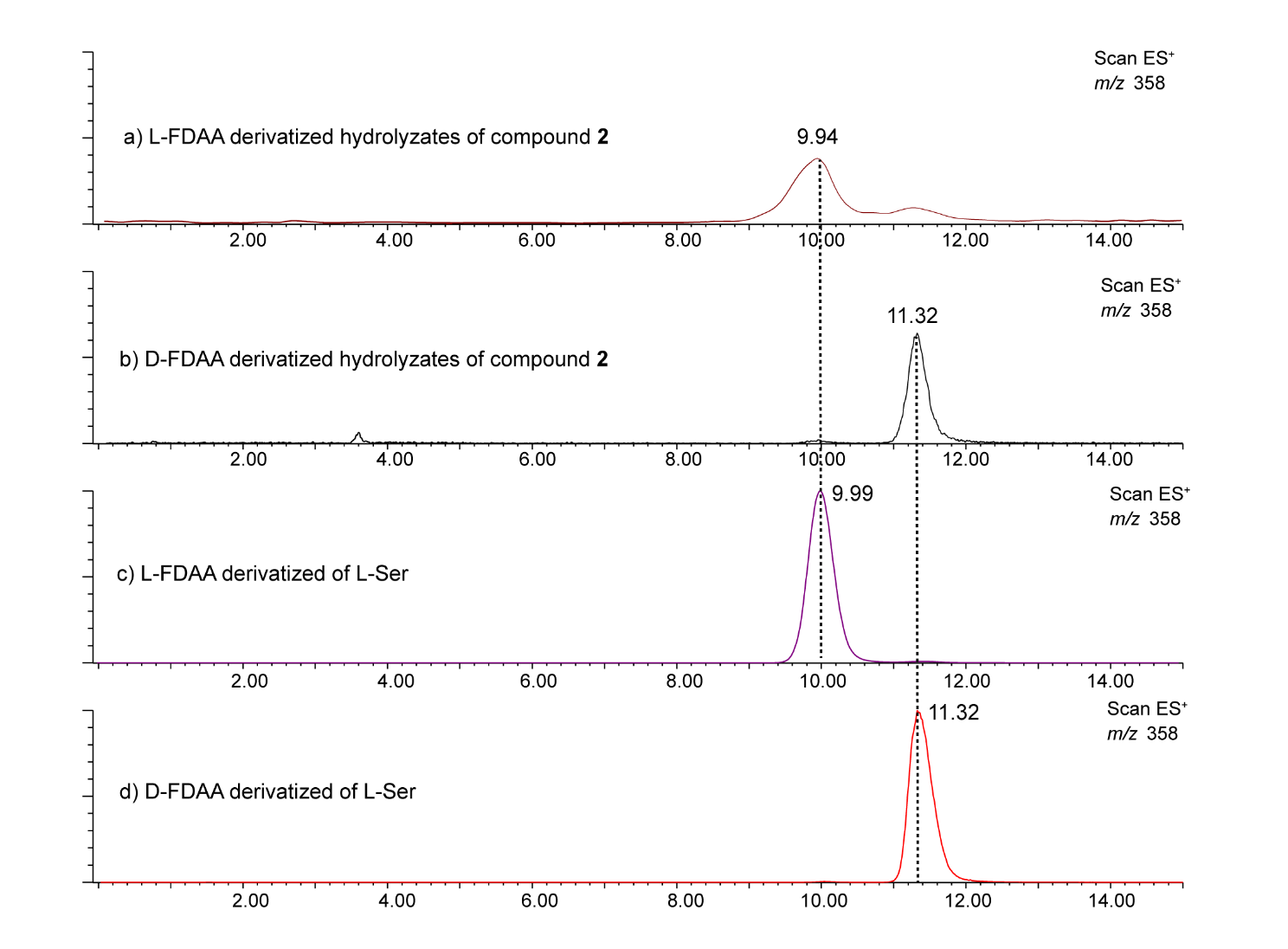


# Fig. S14. The Marfey’s analysis of the acid hydrolysates of compound **2** and *L*-Ser. a) Extracted ion chromatogram at *m/z* 358 for *L*-FDAA derivatized hydrolyzates of **2**. b) Extracted ion chromatogram at *m/z* 358 for *D*-FDAA derivatized hydrolyzates of **2**. c) Extracted ion chromatogram at *m/z* 358 for *L*-FDAA derivatized of *L*-Ser. d) Extracted ion chromatogram at *m/z* 358 for *D*-FDAA derivatized of *L*-Ser.

# Table S1. LC-MS retention times (*t*_R_, min) of the FDAA-derivatized amino acids from **1** and **2** in advanced Marfey’s analysis

| amino acid standards | *m/z* [M+H]^+^ | *L* or *D*-FDAA derivatized amnio acid | | derivatized the hydrolysates of **1** | derivatized the hydrolysates of **2** |
| --- | --- | --- | --- | --- | --- |
| *L*-Ala | 342 | *L* | 7.80 | 7.83 | 7.84 |
|  |  | *D* | 9.22 | 9.21 | 9.22 |
| *L*-Val | 370 | *L* | 11.52 | 11.58 | 11.49 |
|  |  | *D* | 14.18 | 14.23 | 14.27 |
| *L*-Asn | 386 | *L* | 6.22 | 6.23 | 6.18 |
|  |  | *D* | 6.76 | 6.79 | 6.79 |
| *L*-Ser | 358 | *L* | 9.99 | 9.95 | 9.94 |
|  |  | *D* | 11.32 | 11.32 | 11.32 |

# Fig. S15. The ^1^H NMR spectrum of compound **1** in DMSO-*d*_6_ (600 MHz).

# Fig. S16. The ^13^C NMR spectrum of compound **1** in DMSO-*d*_6_ (150 MHz).

# Fig. S17. The ^1^H-^1^H COSY spectrum of compound **1** in DMSO-*d*_6_ (600 MHz).

# Fig. S18. The HSQC spectrum of compound **1** in DMSO-*d*_6_ (600 MHz).

# Fig. S19. The HMBC spectrum of compound **1** in DMSO-*d*_6_ (600 MHz).

# Fig. S20. The ROESY spectrum of compound **1** in DMSO-*d*_6_ (600 MHz).

# Fig. S21. The ^1^H NMR spectrum of compound **2** in DMSO-*d*_6_ (600 MHz).

# Fig. S22. The ^13^C NMR spectrum of compound **2** in DMSO-*d*_6_ (150 MHz).

# Fig. S23. The ^1^H-^1^H COSY spectrum of compound **2** in DMSO-*d*_6_ (600 MHz).

# Fig. S24. The HSQC spectrum of compound **2** in DMSO-*d*_6_ (600 MHz).

# Fig. S25. The HMBC spectrum of compound **2** in DMSO-*d*_6_ (600 MHz).

# Fig. S26. The ROESY spectrum of compound **2** in DMSO-*d*_6_ (600 MHz).

# Table S2. Experimental and Calculated ^13^C NMR Chemical Shifts of **1**a–**1**d

| No. | *δ*_exp_ ^a^ |  | *σ*^x^ (shielding constants)^b^ | | | |  | *δ*_s_ (scaled shifts)^d^ | | | |
| --- | --- | --- | --- | --- | --- | --- | --- | --- | --- | --- | --- |
|  |  |  | **1a** | **1b** | **1c** | **1d** |  | **1a** | **1b** | **1c** | **1d** |
| 1 | 52.2 |  | 127.82 | 127.25 | 134.60 | 125.52 |  | 53.55796 | 53.42514 | 46.94194 | 55.25389 |
| 2 | 34.7 |  | 141.01 | 140.71 | 139.47 | 135.62 |  | 40.96874 | 40.53181 | 42.29128 | 45.58293 |
| 3 | 171.2 |  | 3.57 | 2.41 | 3.59 | 4.83 |  | 172.1407 | 173.0275 | 172.0196 | 170.8601 |
| 4 | 53.4 |  | 120.33 | 115.38 | 115.69 | 118.07 |  | 60.7065 | 64.79463 | 64.99314 | 62.39566 |
| 5 | 28.8 |  | 155.03 | 155.58 | 155.12 | 152.97 |  | 27.58558 | 26.28459 | 27.35596 | 28.96056 |
| 6 | 171.3 |  | 5.09 | 5.93 | 5.61 | 7.52 |  | 170.6918 | 169.6539 | 170.096 | 168.2807 |
| 7 | 20.2 |  | 167.07 | 167.03 | 166.92 | 166.99 |  | 16.09816 | 15.30993 | 16.08414 | 15.53304 |
| 8 | 17.8 |  | 167.26 | 167.59 | 167.74 | 167.86 |  | 15.91379 | 14.77878 | 15.30504 | 14.69701 |
| 9 | 170.3 |  | 5.23 | 4.98 | 4.34 | 5.41 |  | 170.5583 | 170.559 | 171.3082 | 170.3032 |
| 10 | 58.4 |  | 123.58 | 128.52 | 121.54 | 125.98 |  | 57.60117 | 52.20636 | 59.41212 | 54.81481 |
| 11 | 41 |  | 134.22 | 133.20 | 131.69 | 130.58 |  | 47.45072 | 47.71993 | 49.7234 | 50.41003 |
| 12 | 169.1 |  | 6.14 | 11.85 | 11.10 | 10.83 |  | 169.6831 | 163.9861 | 164.8525 | 165.11 |
| 13 | 60.9 |  | 112.52 | 114.91 | 115.36 | 115.00 |  | 68.15481 | 65.24468 | 65.31103 | 65.33384 |
| 14 | 170.3 |  | 9.75 | 6.99 | 8.40 | 5.63 |  | 166.2441 | 168.6394 | 167.4361 | 170.0981 |
| 15 | 49.6 |  | 131.90 | 128.31 | 126.17 | 130.32 |  | 49.664 | 52.40729 | 54.99541 | 50.65741 |
| 16 | 167.6 |  | 12.47 | 11.29 | 11.16 | 11.50 |  | 163.6493 | 164.5184 | 164.793 | 164.4747 |
| 17 | 43.6 |  | 140.64 | 139.82 | 139.92 | 139.37 |  | 41.31784 | 41.38442 | 41.86587 | 41.98902 |
| 18 | 98.7 |  | 79.64 | 81.56 | 80.71 | 81.38 |  | 99.5412 | 97.19585 | 98.3979 | 97.5363 |
| 19 | 132.8 |  | 45.49 | 43.72 | 43.56 | 44.96 |  | 132.1288 | 133.4457 | 133.865 | 132.4183 |
| 20 | 61.4 |  | 121.59 | 122.47 | 123.42 | 121.94 |  | 59.50522 | 58.00405 | 57.61354 | 58.68319 |
| 21 | 36 |  | 149.79 | 150.05 | 145.89 | 145.05 |  | 32.58883 | 31.58183 | 36.16083 | 36.54756 |
| 22 | 172.4 |  | 5.10 | 5.41 | 2.65 | 3.15 |  | 170.6773 | 170.1539 | 172.9263 | 172.4649 |
| 23 | 171.5 |  | 4.78 | 3.45 | 8.30 | 0.64 |  | 170.9846 | 172.0288 | 167.53 | 174.8716 |
| 24 | 48.6 |  | 138.43 | 132.77 | 134.25 | 132.23 |  | 43.42894 | 48.13758 | 47.2779 | 48.83093 |
| 25 | 165.1 |  | 10.58 | 5.65 | 7.31 | 3.91 |  | 165.4453 | 169.9223 | 168.4722 | 171.7373 |
| 26 | 17.6 |  | 168.16 | 171.96 | 171.60 | 171.88 |  | 15.05906 | 10.59014 | 11.62017 | 10.85365 |
| R^2^ |  |  |  |  |  |  |  | 0.9969 | 0.9952 | 0.9950 | 0.9949 |
| Slope (a) |  |  |  |  |  |  |  | 1.0478 | 1.0438 | 1.0474 | 1.0440 |
| Intercept (b) |  |  |  |  |  |  |  | 2.3160 | 3.2394 | 2.4852 | 3.0454 |
| CAME |  |  |  |  |  |  |  | 1.5113 | 2.0405 | 1.9820 | 1.8977 |

# Table S3. Experimental and Calculated ^1^H NMR Chemical Shifts of **1**a–**1**d

| No. | *δ*_exp_ ^a^ |  | *σ*^x^ (shielding constants)^b^ | | | |  | *δ*_s_ (scaled shifts)^d^ | | | |
| --- | --- | --- | --- | --- | --- | --- | --- | --- | --- | --- | --- |
|  |  |  | **1a** | **1b** | **1c** | **1d** |  | **1a** | **1b** | **1c** | **1d** |
| 1 | 4.20 |  | 27.015 | 27.138 | 27.244 | 27.038 |  | 4.3034 | 4.3844 | 4.2554 | 4.4820 |
| 2 | 1.16 |  | 30.453 | 30.480 | 30.531 | 30.484 |  | 0.9875 | 0.9815 | 0.9700 | 0.9217 |
| 3 | 1.16 |  | 30.453 | 30.480 | 30.531 | 30.484 |  | 0.9875 | 0.9815 | 0.9700 | 0.9217 |
| 4 | 1.16 |  | 30.453 | 30.480 | 30.531 | 30.484 |  | 0.9875 | 0.9815 | 0.9700 | 0.9217 |
| 5 | 4.17 |  | 26.627 | 27.353 | 27.240 | 27.330 |  | 4.6777 | 4.1655 | 4.2594 | 4.1803 |
| 6 | 3.01 |  | 29.002 | 30.113 | 30.124 | 29.700 |  | 2.3870 | 1.3552 | 1.3768 | 1.7317 |
| 7 | 2.11 |  | 28.837 | 29.126 | 29.310 | 28.795 |  | 2.5461 | 2.3601 | 2.1904 | 2.6667 |
| 8 | 4.16 |  | 27.678 | 28.350 | 28.265 | 27.358 |  | 3.6640 | 3.1503 | 3.2349 | 4.1514 |
| 9 | 1.97 |  | 28.593 | 28.925 | 28.762 | 29.484 |  | 2.7814 | 2.5648 | 2.7381 | 1.9549 |
| 10 | 0.74 |  | 30.741 | 30.714 | 30.717 | 30.646 |  | 0.7097 | 0.7432 | 0.7841 | 0.7543 |
| 11 | 0.74 |  | 30.741 | 30.714 | 30.717 | 30.646 |  | 0.7097 | 0.7432 | 0.7841 | 0.7543 |
| 12 | 0.74 |  | 30.741 | 30.714 | 30.717 | 30.646 |  | 0.7097 | 0.7432 | 0.7841 | 0.7543 |
| 13 | 0.60 |  | 30.834 | 30.736 | 30.753 | 30.681 |  | 0.6200 | 0.7208 | 0.7481 | 0.7182 |
| 14 | 0.60 |  | 30.834 | 30.736 | 30.753 | 30.681 |  | 0.6200 | 0.7208 | 0.7481 | 0.7182 |
| 15 | 0.60 |  | 30.834 | 30.736 | 30.753 | 30.681 |  | 0.6200 | 0.7208 | 0.7481 | 0.7182 |
| 16 | 3.84 |  | 27.088 | 26.752 | 27.318 | 26.882 |  | 4.2330 | 4.7774 | 4.1814 | 4.6431 |
| 17 | 3.50 |  | 27.523 | 28.064 | 27.395 | 27.853 |  | 3.8135 | 3.4415 | 4.1044 | 3.6399 |
| 18 | 3.50 |  | 27.672 | 27.276 | 27.617 | 27.100 |  | 3.6698 | 4.2439 | 3.8826 | 4.4179 |
| 19 | 3.86 |  | 27.664 | 28.025 | 27.806 | 27.955 |  | 3.6775 | 3.4812 | 3.6937 | 3.5346 |
| 20 | 2.74 |  | 28.809 | 29.105 | 28.653 | 28.630 |  | 2.5731 | 2.3815 | 2.8471 | 2.8372 |
| 21 | 4.84 |  | 26.694 | 27.231 | 26.700 | 27.722 |  | 4.6130 | 4.2897 | 4.7991 | 3.7753 |
| 22 | 2.75 |  | 29.002 | 28.513 | 28.477 | 27.855 |  | 2.3870 | 2.9843 | 3.0230 | 3.6379 |
| 23 | 2.88 |  | 28.622 | 28.218 | 29.273 | 29.704 |  | 2.7535 | 3.2847 | 2.2274 | 1.7276 |
| 24 | 3.49 |  | 28.041 | 27.927 | 27.898 | 27.862 |  | 3.3139 | 3.5810 | 3.6017 | 3.6306 |
| 25 | 4.07 |  | 26.968 | 27.166 | 27.197 | 27.312 |  | 4.3488 | 4.3559 | 4.3023 | 4.1989 |
| 26 | 5.28 |  | 26.083 | 26.107 | 26.075 | 26.213 |  | 5.2024 | 5.4342 | 5.4238 | 5.3343 |
| 27 | 7.16 |  | 24.080 | 24.121 | 24.116 | 24.307 |  | 7.1343 | 7.4564 | 7.3818 | 7.3035 |
| R^2^ |  |  |  |  |  |  |  | 0.9685 | 0.9189 | 0.9319 | 0.9148 |
| Slope (a) |  |  |  |  |  |  |  | 1.0368 | 0.9821 | 1.0005 | 0.9679 |
| Intercept (b) |  |  |  |  |  |  |  | 0.3752 | 0.4081 | 0.3505 | 0.4759 |
| CAME |  |  |  |  |  |  |  | 0.1397 | 0.2068 | 0.1813 | 0.2073 |

# Table S4. DP4+ Probabilities Computed for **1**a-**1**d

|  | **1a** | **1b** | **1c** | **1d** |
| --- | --- | --- | --- | --- |
| DP4+ (H data) | 99.90% | 0.00% | 0.00% | 0.10% |
| DP4+ (C data) | 100.00% | 0.00% | 0.00% | 0.00% |
| DP4+ (all data) | 100.00% | 0.00% | 0.00% | 0.00% |


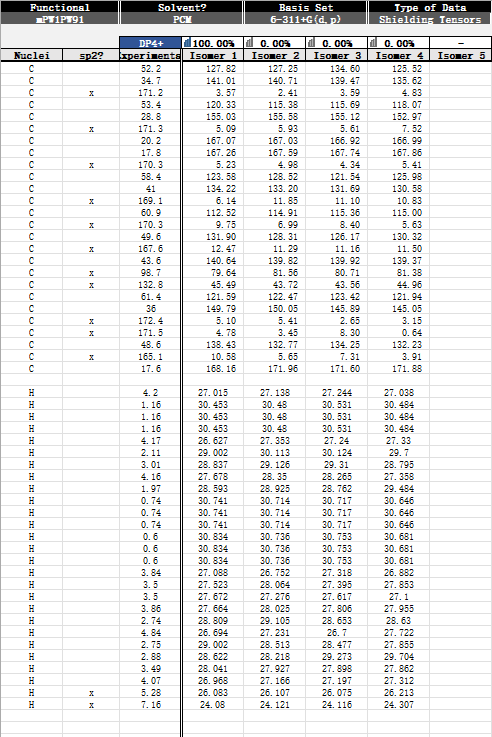


# Table S5. Lowest-energy Conformers Optimized at the M062X/6-311+G (d, p) Level of **1a** with Relative Energies < 3.0 kcal/mol

| **1a_C1** | G 2697.1313 Ha  Boltzman pop. 6.55% | | | **1a_C2** | G 2697.1313 Ha  Boltzman pop. 6.54% | | | **1a_C3** | G 2697.1313 Ha  Boltzman pop. 6.54% | |
| --- | --- | --- | --- | --- | --- | --- | --- | --- | --- | --- |
| 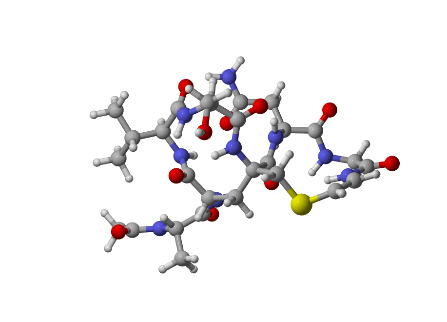 | | | | 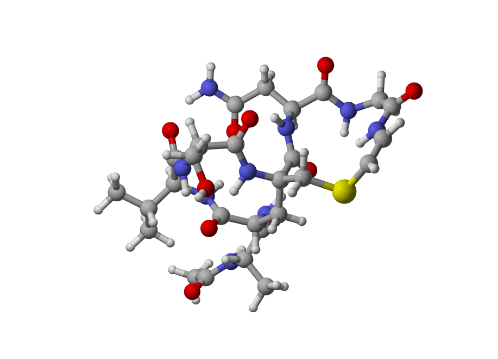 | | | | 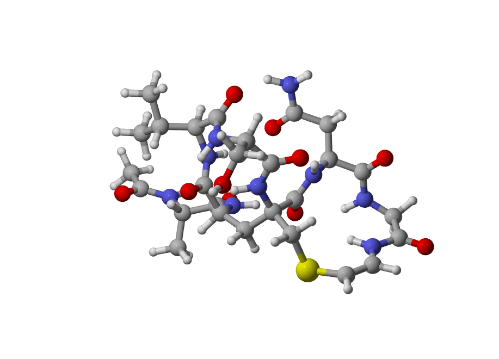 | | |
| **1a_C4** | | G 2697.1313 Ha  Boltzman pop. 6.55% | | **1a_C5** | | G 2697.1306 Ha  Boltzman pop. 41.44% | | **1a_C6** | | G 2697.1280 Ha  Boltzman pop. 1.22% |
| 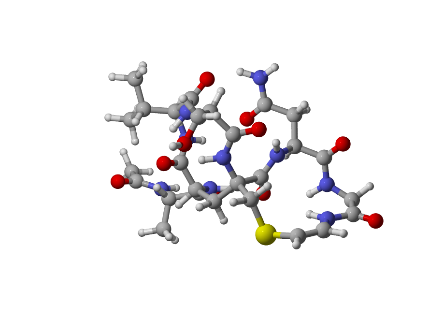 | | | | 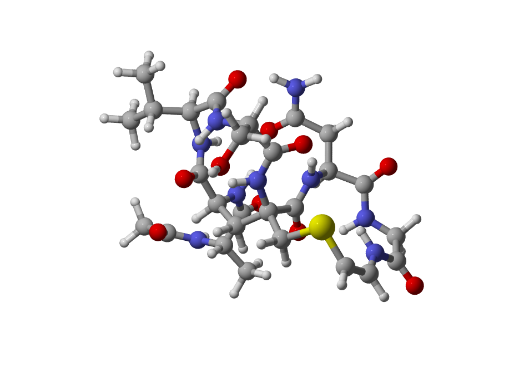 | | | | 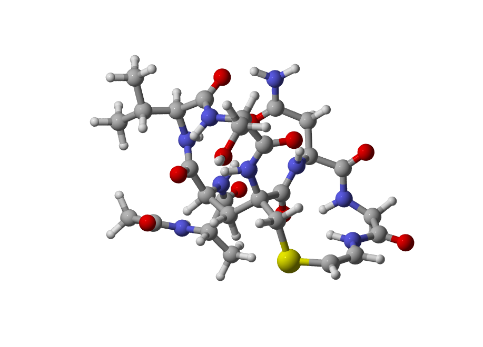 | | |
| **1a_C7** | | | G 2697.1299 Ha  Boltzman pop. 7.96% | **1a_C8** | | | G 2697.1321 Ha  Boltzman pop. 23.2% |  | | |
| 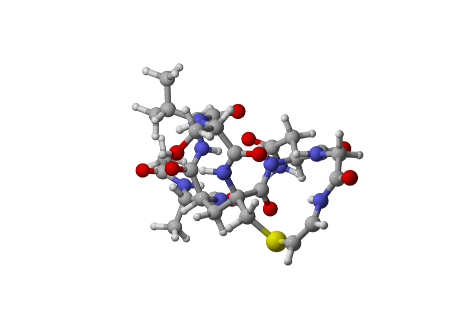 | | | | 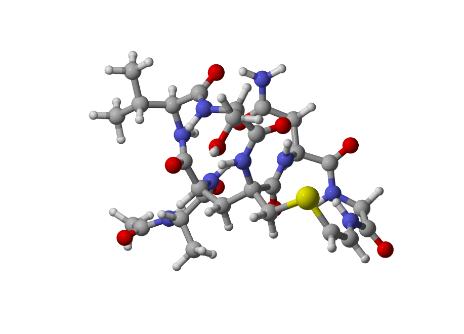 | | | |  |  |  |

# Table S6. Atomic Coordinates for the Lowest-energy Conformers of **1a** (**1a_C1**–**1a_C8**)

| **Atoms** | **1a_C1** | | | **1a_C2** | | | **1a_C3** | | | **1a_C4** | | | **1a_C5** | | |
| --- | --- | --- | --- | --- | --- | --- | --- | --- | --- | --- | --- | --- | --- | --- | --- |
|  | **x** | **y** | **z** | **x** | **y** | **z** | **x** | **y** | **z** | **x** | **y** | **z** | **x** | **y** | **z** |
| **N**  **C**  **C**  **C**  **N**  **O**  **C**  **C**  **C**  **C**  **C**  **N**  **C**  **O**  **C**  **O**  **C**  **C**  **C**  **O**  **C**  **C**  **N**  **O**  **C**  **C**  **N**  **N**  **S**  **C**  **C**  **C**  **O**  **N**  **C**  **C**  **O**  **N**  **O**  **C**  **C**  **N**  **C**  **C**  **O**  **C**  **O**  **H**  **H**  **H**  **H**  **H**  **H**  **H**  **H**  **H**  **H**  **H**  **H**  **H**  **H**  **H**  **H**  **H**  **H**  **H**  **H**  **H**  **H**  **H**  **H**  **H**  **H**  **H**  **H**  **H**  **H**  **H**  **H**  **H**  **H**  **H**  **H**  **H**  **H**  **H**  **H**  **HN** | **-3.10086** | **5.19516** | **-3.70760** | **-3.10058** | **5.19304** | **-3.71915** | **-3.10003** | **5.19366** | **-3.70817** | **-3.09806** | **5.19218** | **-3.70027** | **-3.07772** | **6.10809** | **-3.97057** |
| **C** | **-3.89531** | **5.44372** | **-2.51361** | **-3.89693** | **5.41104** | **-2.52045** | **-3.89311** | **5.41097** | **-2.50719** | **-3.89182** | **5.43318** | **-2.50425** | **-4.04748** | **5.95941** | **-2.89126** |
| **C** | **-3.11509** | **5.16100** | **-1.18342** | **-3.1146** | **5.10717** | **-1.19626** | **-3.10797** | **5.10393** | **-1.18539** | **-3.1097** | **5.14519** | **-1.17633** | **-3.4883** | **5.3203** | **-1.58205** |
| **C** | **-4.62821** | **6.80789** | **-2.48076** | **-4.64048** | **6.76846** | **-2.45975** | **-4.63451** | **6.76939** | **-2.44262** | **-4.62733** | **6.79574** | **-2.46374** | **-4.84107** | **7.24643** | **-2.58476** |
| **N** | **-4.28965** | **7.74493** | **-3.38643** | **-4.30915** | **7.72665** | **-3.34573** | **-4.30379** | **7.7285** | **-3.3278** | **-4.29136** | **7.73816** | **-3.3648** | **-4.67018** | **8.32362** | **-3.38265** |
| **O** | **-5.48464** | **6.97374** | **-1.59415** | **-5.49862** | **6.90911** | **-1.57044** | **-5.49036** | **6.90988** | **-1.55107** | **-5.48348** | **6.95523** | **-1.5757** | **-5.61509** | **7.23821** | **-1.61354** |
| **C** | **-4.79863** | **9.13648** | **-3.42234** | **-4.82974** | **9.1143** | **-3.35365** | **-4.82235** | **9.11692** | **-3.33233** | **-4.80331** | **9.12878** | **-3.39319** | **-5.17861** | **9.68395** | **-3.09694** |
| **C** | **-6.34169** | **9.27230** | **-3.48661** | **-6.37389** | **9.23872** | **-3.41491** | **-6.36646** | **9.24374** | **-3.38989** | **-6.34671** | **9.26177** | **-3.45536** | **-6.70994** | **9.79722** | **-2.90528** |
| **C** | **-4.11764** | **9.94876** | **-2.29668** | **-4.15517** | **9.90926** | **-2.21192** | **-4.14403** | **9.90912** | **-2.19089** | **-4.12298** | **9.9367** | **-2.26402** | **-4.3045** | **10.26397** | **-1.96068** |
| **C** | **-6.72459** | **10.76123** | **-3.54175** | **-6.76889** | **10.72529** | **-3.44134** | **-6.75933** | **10.73093** | **-3.41308** | **-6.73268** | **10.75018** | **-3.50306** | **-7.09336** | **11.27367** | **-2.71087** |
| **C** | **-6.90798** | **8.52069** | **-4.69935** | **-6.93468** | **8.50594** | **-4.64163** | **-6.9311** | **8.51374** | **-4.6165** | **-6.91268** | **8.51478** | **-4.67109** | **-7.45318** | **9.18737** | **-4.10269** |
| **N** | **-2.72898** | **7.44691** | **-0.46460** | **-2.74626** | **7.38077** | **-0.43055** | **-2.73464** | **7.37576** | **-0.41686** | **-2.72738** | **7.42816** | **-0.44629** | **-2.86747** | **7.36867** | **-0.43632** |
| **C** | **-2.29756** | **8.65168** | **-0.01797** | **-2.32412** | **8.57981** | **0.03981** | **-2.30968** | **8.57343** | **0.05445** | **-2.29788** | **8.6316** | **0.00584** | **-2.33735** | **8.57052** | **-0.09538** |
| **O** | **-1.13129** | **8.92477** | **0.28833** | **-1.15983** | **8.85595** | **0.35086** | **-1.14427** | **8.84743** | **0.36319** | **-1.13175** | **8.90559** | **0.31179** | **-1.13559** | **8.85725** | **-0.08007** |
| **C** | **-3.39695** | **9.75333** | **0.03690** | **-3.43202** | **9.67161** | **0.11708** | **-3.41585** | **9.66669** | **0.13606** | **-3.39957** | **9.73062** | **0.06781** | **-3.38141** | **9.67861** | **0.24037** |
| **O** | **-0.62443** | **5.21525** | **-2.37076** | **-0.62463** | **5.20493** | **-2.38209** | **-0.62079** | **5.20033** | **-2.37726** | **-0.62041** | **5.21024** | **-2.36594** | **-0.81032** | **4.8868** | **-2.26591** |
| **C** | **-1.56242** | **5.68267** | **0.77469** | **-1.56544** | **5.60061** | **0.77201** | **-1.55321** | **5.59205** | **0.77985** | **-1.55581** | **5.66006** | **0.78266** | **-2.01306** | **5.18694** | **0.43954** |
| **C** | **-0.83201** | **6.18976** | **-1.62679** | **-0.83985** | **6.1626** | **-1.61872** | **-0.83283** | **6.15702** | **-1.61175** | **-0.82927** | **6.18074** | **-1.61709** | **-1.08644** | **5.98747** | **-1.7631** |
| **C** | **-2.03669** | **6.14976** | **-0.63990** | **-2.0438** | **6.093** | **-0.63262** | **-2.03443** | **6.08739** | **-0.62278** | **-2.03264** | **6.13334** | **-0.62899** | **-2.32292** | **6.03909** | **-0.82143** |
| **O** | **3.16687** | **7.70779** | **-1.76644** | **3.14627** | **7.71689** | **-1.72967** | **3.15509** | **7.70585** | **-1.72918** | **3.16612** | **7.70835** | **-1.75508** | **2.90986** | **7.47471** | **-2.57086** |
| **C** | **2.32282** | **6.95460** | **-2.23801** | **2.30838** | **6.96595** | **-2.21559** | **2.31512** | **6.95693** | **-2.21461** | **2.32295** | **6.9555** | **-2.2288** | **1.95935** | **6.71253** | **-2.71965** |
| **C** | **0.94567** | **7.44565** | **-2.72187** | **0.9268** | **7.45465** | **-2.68912** | **0.93314** | **7.44834** | **-2.68416** | **0.94408** | **7.44585** | **-2.70851** | **0.54891** | **7.20289** | **-3.14841** |
| **N** | **-0.03013** | **7.26598** | **-1.63850** | **-0.04678** | **7.24535** | **-1.60912** | **-0.03826** | **7.23866** | **-1.60229** | **-0.02986** | **7.25883** | **-1.62473** | **-0.39515** | **7.10906** | **-2.02728** |
| **O** | **4.43160** | **4.42211** | **0.49700** | **4.43913** | **4.39623** | **0.46785** | **4.44883** | **4.38018** | **0.45931** | **4.44035** | **4.41437** | **0.49142** | **3.5928** | **3.06154** | **-0.21325** |
| **C** | **3.46398** | **4.60245** | **-0.22520** | **3.46987** | **4.58334** | **-0.25043** | **3.47812** | **4.56956** | **-0.25641** | **3.4715** | **4.59599** | **-0.22881** | **2.90477** | **3.99804** | **-0.59341** |
| **C** | **3.62633** | **4.93513** | **-1.71762** | **3.62915** | **4.9476** | **-1.73578** | **3.63433** | **4.93598** | **-1.74154** | **3.63138** | **4.93628** | **-1.71977** | **3.17091** | **4.69755** | **-1.93309** |
| **N** | **2.48991** | **5.59960** | **-2.33154** | **2.48694** | **5.61457** | **-2.33611** | **2.49164** | **5.60554** | **-2.33804** | **2.49279** | **5.60135** | **-2.329** | **2.01259** | **5.36973** | **-2.49102** |
| **N** | **2.16028** | **4.49863** | **0.21385** | **2.16719** | **4.45982** | **0.18652** | **2.17632** | **4.44708** | **0.18345** | **2.16854** | **4.48695** | **0.21125** | **1.84423** | **4.50178** | **0.12809** |
| **S** | **-0.83835** | **3.97371** | **0.82232** | **-0.82663** | **3.89741** | **0.78413** | **-0.81681** | **3.88777** | **0.78726** | **-0.82821** | **3.95238** | **0.82099** | **-0.61285** | **5.86111** | **1.46476** |
| **C** | **0.52755** | **4.13865** | **1.95471** | **0.53797** | **4.05059** | **1.91974** | **0.5507** | **4.03711** | **1.91987** | **0.53885** | **4.11459** | **1.95238** | **0.26142** | **4.38335** | **1.94748** |
| **C** | **1.79810** | **4.29805** | **1.53327** | **1.80705** | **4.22926** | **1.50161** | **1.81901** | **4.21477** | **1.499** | **1.80849** | **4.27893** | **1.5301** | **1.35313** | **3.93951** | **1.29205** |
| **C** | **-4.04746** | **9.82172** | **1.41598** | **-4.08281** | **9.70724** | **1.49728** | **-4.06337** | **9.7009** | **1.51785** | **-4.04894** | **9.79019** | **1.44784** | **-3.92285** | **9.55394** | **1.66277** |
| **O** | **-4.73320** | **8.58526** | **1.62604** | **-4.7594** | **8.46179** | **1.68232** | **-4.74112** | **8.45601** | **1.70239** | **-4.73198** | **8.55128** | **1.652** | **-4.71198** | **8.36316** | **1.72131** |
| **N** | **-4.39832** | **9.57455** | **-1.01029** | **-4.43217** | **9.50636** | **-0.93346** | **-4.41867** | **9.50459** | **-0.91241** | **-4.40159** | **9.55524** | **-0.97936** | **-4.47612** | **9.70623** | **-0.72623** |
| **C** | **0.97649** | **8.90002** | **-3.18144** | **0.94487** | **8.91794** | **-3.12004** | **0.95226** | **8.91231** | **-3.11268** | **0.97104** | **8.90244** | **-3.16119** | **0.61622** | **8.64827** | **-3.66168** |
| **C** | **-0.40612** | **9.35696** | **-3.65423** | **-0.44171** | **9.37227** | **-3.58354** | **-0.43476** | **9.36933** | **-3.5722** | **-0.4132** | **9.35861** | **-3.62984** | **-0.75747** | **9.29917** | **-3.79297** |
| **O** | **-1.24527** | **8.56112** | **-4.08622** | **-1.27516** | **8.57759** | **-4.02859** | **-1.2704** | **8.57655** | **-4.0165** | **-1.25165** | **8.56284** | **-4.06338** | **-1.73934** | **8.69106** | **-4.24606** |
| **N** | **-0.63481** | **10.69242** | **-3.59081** | **-0.68069** | **10.70459** | **-3.49651** | **-0.6716** | **10.70187** | **-3.48264** | **-0.64432** | **10.69339** | **-3.56082** | **-0.83962** | **10.58972** | **-3.40592** |
| **O** | **-3.32049** | **10.84952** | **-2.55951** | **-3.36555** | **10.82174** | **-2.45651** | **-3.35379** | **10.82096** | **-2.43584** | **-3.32793** | **10.84047** | **-2.52298** | **-3.44888** | **11.12121** | **-2.19305** |
| **C** | **-3.53710** | **4.70859** | **-4.90803** | **-3.5331** | **4.7287** | **-4.92963** | **-3.53616** | **4.73158** | **-4.91823** | **-3.53464** | **4.71169** | **-4.90299** | **-2.68222** | **5.15666** | **-4.87095** |
| **C** | **-5.03233** | **4.40828** | **-5.09095** | **-5.02595** | **4.42072** | **-5.11901** | **-5.02989** | **4.42588** | **-5.10433** | **-5.02954** | **4.40998** | **-5.08615** | **-3.40745** | **3.79284** | **-4.91577** |
| **N** | **-5.33023** | **4.58373** | **-6.50306** | **-5.32512** | **4.62314** | **-6.52723** | **-5.33226** | **4.6305** | **-6.51156** | **-5.32896** | **4.59261** | **-6.49703** | **-4.31178** | **3.78072** | **-6.06646** |
| **C** | **-6.57246** | **4.91530** | **-6.95143** | **-6.57007** | **4.95331** | **-6.96899** | **-6.57781** | **4.96317** | **-6.94976** | **-6.57249** | **4.92296** | **-6.94266** | **-5.63033** | **4.1192** | **-5.97092** |
| **C** | **-6.71750** | **5.06203** | **-8.45607** | **-6.71647** | **5.12875** | **-8.47042** | **-6.72753** | **5.14167** | **-8.4505** | **-6.71924** | **5.077** | **-8.4464** | **-6.41265** | **4.13054** | **-7.27099** |
| **O** | **-2.75023** | **4.48308** | **-5.82738** | **-2.74451** | **4.52849** | **-5.85338** | **-2.75015** | **4.53167** | **-5.84423** | **-2.74826** | **4.49253** | **-5.82431** | **-1.7757** | **5.37985** | **-5.67244** |
| **C** | **-5.35070** | **2.97195** | **-4.62085** | **-5.3332** | **2.97254** | **-4.67882** | **-5.33803** | **2.97755** | **-4.66526** | **-5.34518** | **2.97063** | **-4.62351** | **-2.38644** | **2.65756** | **-5.01565** |
| **O** | **-7.52440** | **5.08424** | **-6.18691** | **-7.52341** | **5.09861** | **-6.20134** | **-7.52904** | **5.1087** | **-6.17956** | **-7.52424** | **5.08528** | **-6.17647** | **-6.16634** | **4.38553** | **-4.89242** |
| **H** | **-2.08790** | **5.19050** | **-3.59366** | **-2.08763** | **5.19397** | **-3.60529** | **-2.08679** | **5.19298** | **-3.59676** | **-2.085** | **5.18867** | **-3.58733** | **-2.46932** | **6.93138** | **-3.99512** |
| **H** | **-4.71908** | **4.72441** | **-2.47521** | **-4.7151** | **4.68472** | **-2.49692** | **-4.71231** | **4.68584** | **-2.48276** | **-4.71417** | **4.71209** | **-2.46869** | **-4.83231** | **5.27919** | **-3.24294** |
| **H** | **-3.87578** | **5.07452** | **-0.40146** | **-3.87457** | **4.99895** | **-0.41631** | **-3.86619** | **4.99552** | **-0.40376** | **-3.86941** | **5.05331** | **-0.39403** | **-4.33201** | **5.21595** | **-0.89316** |
| **H** | **-2.64648** | **4.18183** | **-1.29475** | **-2.63854** | **4.13408** | **-1.32748** | **-2.6336** | **4.13038** | **-1.31932** | **-2.63938** | **4.16748** | **-1.2931** | **-3.1332** | **4.31882** | **-1.82786** |
| **H** | **-3.45860** | **7.56758** | **-3.94533** | **-3.47599** | **7.56813** | **-3.90719** | **-3.47226** | **7.56961** | **-3.89155** | **-3.46019** | **7.56569** | **-3.92507** | **-3.9156** | **8.29486** | **-4.05958** |
| **H** | **-4.38831** | **9.56042** | **-4.34211** | **-4.42328** | **9.56002** | **-4.26479** | **-4.41729** | **9.5635** | **-4.24367** | **-4.39472** | **9.55825** | **-4.31115** | **-4.90967** | **10.27627** | **-3.97565** |
| **H** | **-6.77115** | **8.83375** | **-2.58068** | **-6.79948** | **8.77946** | **-2.51746** | **-6.79069** | **8.78369** | **-2.4922** | **-6.77446** | **8.81808** | **-2.55113** | **-6.99785** | **9.23759** | **-2.01064** |
| **H** | **-7.81299** | **10.86344** | **-3.59425** | **-7.85813** | **10.81964** | **-3.49104** | **-7.84854** | **10.82696** | **-3.46018** | **-7.82134** | **10.85046** | **-3.55386** | **-8.17428** | **11.36532** | **-2.56463** |
| **H** | **-6.38171** | **11.31374** | **-2.66091** | **-6.42969** | **11.26368** | **-2.55039** | **-6.41734** | **11.26741** | **-2.52205** | **-6.38992** | **11.29923** | **-2.62002** | **-6.60221** | **11.71798** | **-1.83872** |
| **H** | **-6.30067** | **11.24792** | **-4.42850** | **-6.34978** | **11.23225** | **-4.31897** | **-6.34143** | **11.23866** | **-4.29084** | **-6.31074** | **11.24189** | **-4.38798** | **-6.8236** | **11.87311** | **-3.58899** |
| **H** | **-7.99457** | **8.64368** | **-4.74451** | **-8.02218** | **8.62145** | **-4.68438** | **-8.01852** | **8.63098** | **-4.65664** | **-7.99953** | **8.63598** | **-4.71474** | **-8.53463** | **9.28614** | **-3.96289** |
| **H** | **-6.70846** | **7.44759** | **-4.66264** | **-6.72695** | **7.43389** | **-4.62527** | **-6.725** | **7.44135** | **-4.60227** | **-6.71114** | **7.44189** | **-4.63951** | **-7.22745** | **8.12485** | **-4.22437** |
| **H** | **-6.48964** | **8.91387** | **-5.63472** | **-6.51959** | **8.91988** | **-5.56946** | **-6.51742** | **8.92852** | **-5.54459** | **-6.49589** | **8.91303** | **-5.60501** | **-7.18888** | **9.70306** | **-5.03477** |
| **H** | **-3.73773** | **7.35016** | **-0.47178** | **-3.75423** | **7.27615** | **-0.4391** | **-3.74277** | **7.27257** | **-0.42308** | **-3.73594** | **7.3293** | **-0.45245** | **-3.86762** | **7.30822** | **-0.27458** |
| **H** | **-2.87033** | **10.69104** | **-0.14966** | **-2.91266** | **10.61692** | **-0.05052** | **-2.89551** | **10.61152** | **-0.03119** | **-2.87516** | **10.67046** | **-0.1142** | **-2.82698** | **10.61441** | **0.1557** |
| **H** | **-2.43270** | **5.71901** | **1.43584** | **-2.43591** | **5.6158** | **1.43375** | **-2.42198** | **5.60739** | **1.44381** | **-2.42539** | **5.69133** | **1.445** | **-1.73056** | **4.1822** | **0.12187** |
| **H** | **-0.81277** | **6.37984** | **1.15269** | **-0.82177** | **6.29625** | **1.1644** | **-0.80755** | **6.28598** | **1.17146** | **-0.80714** | **6.35686** | **1.1633** | **-2.91279** | **5.13233** | **1.05818** |
| **H** | **0.59949** | **6.81990** | **-3.54697** | **0.58557** | **6.84223** | **-3.5262** | **0.58913** | **6.83779** | **-3.52147** | **0.59819** | **6.82325** | **-3.53611** | **0.16822** | **6.54337** | **-3.93496** |
| **H** | **-0.17258** | **8.00345** | **-0.94147** | **-0.19497** | **7.96763** | **-0.89752** | **-0.18364** | **7.95988** | **-0.88903** | **-0.1732** | **7.99272** | **-0.92411** | **-0.49575** | **7.89627** | **-1.37363** |
| **H** | **4.50461** | **5.57783** | **-1.79616** | **4.50183** | **5.59933** | **-1.80125** | **4.50783** | **5.58651** | **-1.80811** | **4.50823** | **5.58118** | **-1.79624** | **3.95796** | **5.44575** | **-1.79404** |
| **H** | **3.83607** | **4.00419** | **-2.25419** | **3.84688** | **4.0296** | **-2.29112** | **3.84932** | **4.01852** | **-2.29886** | **3.84238** | **4.00842** | **-2.26116** | **3.55515** | **3.92998** | **-2.61168** |
| **H** | **1.72119** | **5.05662** | **-2.70987** | **1.72255** | **5.07272** | **-2.7247** | **1.72559** | **5.0654** | **-2.72574** | **1.72457** | **5.05857** | **-2.70865** | **1.14582** | **4.84893** | **-2.6238** |
| **H** | **1.40509** | **4.58538** | **-0.45733** | **1.41107** | **4.55432** | **-0.48257** | **1.4187** | **4.54337** | **-0.48371** | **1.41229** | **4.57523** | **-0.45855** | **1.33961** | **5.29822** | **-0.24503** |
| **H** | **0.32826** | **4.00430** | **3.01202** | **0.34** | **3.89291** | **2.97406** | **0.35503** | **3.87789** | **2.9744** | **0.34124** | **3.97443** | **3.00924** | **-0.05506** | **3.87933** | **2.85417** |
| **H** | **2.63341** | **4.29374** | **2.22636** | **2.64254** | **4.21772** | **2.19441** | **2.65615** | **4.20091** | **2.18977** | **2.64471** | **4.27298** | **2.22209** | **1.91578** | **3.07905** | **1.64031** |
| **H** | **-3.26958** | **9.97811** | **2.17444** | **-3.30604** | **9.85408** | **2.25878** | **-3.28464** | **9.8454** | **2.2778** | **-3.27069** | **9.94412** | **2.20643** | **-3.08301** | **9.50961** | **2.36817** |
| **H** | **-4.74452** | **10.66879** | **1.43325** | **-4.78614** | **10.54861** | **1.53164** | **-4.76552** | **10.54311** | **1.55529** | **-4.74769** | **10.63576** | **1.47035** | **-4.53329** | **10.43887** | **1.88388** |
| **H** | **-5.29227** | **8.64626** | **2.41004** | **-5.31725** | **8.50215** | **2.46852** | **-5.29887** | **8.4965** | **2.48867** | **-5.29068** | **8.60707** | **2.43667** | **-5.12983** | **8.28493** | **2.58731** |
| **H** | **-5.06806** | **8.81562** | **-0.89457** | **-5.096** | **8.74011** | **-0.83304** | **-5.08324** | **8.73902** | **-0.81171** | **-5.06968** | **8.79439** | **-0.86685** | **-5.16452** | **8.95822** | **-0.66378** |
| **H** | **1.67909** | **8.98879** | **-4.01871** | **1.64645** | **9.02926** | **-3.95547** | **1.65206** | **9.02409** | **-3.94955** | **1.67223** | **8.9967** | **-3.99903** | **1.08408** | **8.6404** | **-4.65248** |
| **H** | **1.36359** | **9.54486** | **-2.38616** | **1.32661** | **9.55038** | **-2.3123** | **1.33674** | **9.54288** | **-2.30476** | **1.35786** | **9.54436** | **-2.36342** | **1.27344** | **9.2343** | **-3.01352** |
| **H** | **-1.60557** | **10.99035** | **-3.64176** | **-1.6538** | **10.99578** | **-3.5411** | **-1.6444** | **10.99447** | **-3.52451** | **-1.61576** | **10.98958** | **-3.60879** | **-1.76636** | **10.99407** | **-3.28338** |
| **H** | **-0.00428** | **11.28402** | **-3.07017** | **-0.05447** | **11.29168** | **-2.96564** | **-0.04333** | **11.28726** | **-2.95232** | **-0.01421** | **11.28399** | **-3.03853** | **-0.07738** | **11.01755** | **-2.90227** |
| **H** | **-5.66002** | **5.11185** | **-4.53760** | **-5.65904** | **5.1078** | **-4.55129** | **-5.66066** | **5.11308** | **-4.53416** | **-5.65779** | **5.10955** | **-4.52837** | **-4.039** | **3.64133** | **-4.04119** |
| **H** | **-4.54600** | **4.47080** | **-7.13428** | **-4.53998** | **4.52939** | **-7.16046** | **-4.54881** | **4.53659** | **-7.14685** | **-4.54505** | **4.48453** | **-7.1295** | **-3.88884** | **3.7023** | **-6.98147** |
| **H** | **-7.44786** | **4.32875** | **-8.81044** | **-7.43502** | **4.39154** | **-8.84055** | **-7.45248** | **4.41017** | **-8.81937** | **-7.44622** | **4.34204** | **-8.80423** | **-7.27029** | **3.46032** | **-7.16769** |
| **H** | **-7.11823** | **6.05570** | **-8.67450** | **-7.13256** | **6.12028** | **-8.66869** | **-7.13712** | **6.13646** | **-8.64618** | **-7.12493** | **6.06984** | **-8.65929** | **-6.80166** | **5.13926** | **-7.43752** |
| **H** | **-5.77956** | **4.92402** | **-9.00090** | **-5.77618** | **5.01643** | **-9.01711** | **-5.78972** | **5.02356** | **-9.00021** | **-5.78099** | **4.9464** | **-8.99255** | **-5.82243** | **3.82714** | **-8.13991** |
| **H** | **-6.41018** | **2.76798** | **-4.79341** | **-6.39104** | **2.76394** | **-4.85584** | **-6.39658** | **2.77062** | **-4.83993** | **-6.40447** | **2.76588** | **-4.79625** | **-2.90313** | **1.69457** | **-5.06262** |
| **H** | **-5.14304** | **2.83537** | **-3.55423** | **-5.1246** | **2.81558** | **-3.6152** | **-5.127** | **2.81893** | **-3.60236** | **-5.13637** | **2.8287** | **-3.55782** | **-1.72913** | **2.6668** | **-4.14158** |
| **H** | **-4.74910** | **2.25141** | **-5.18268** | **-4.72593** | **2.26848** | **-5.25524** | **-4.73315** | **2.2734** | **-5.24409** | **-4.74287** | **2.2541** | **-5.1897** | **-1.75972** | **2.78086** | **-5.90322** |
| **Atoms** | **1a_C6** | | | **1a_C7** | | | **1a_C8** | | |  |  |  |  |  |  |
|  | **x** | **y** | **z** | **x** | **y** | **z** | **x** | **y** | **z** |  |  |  |  |  |  |
| **N**  **C**  **C**  **C**  **N**  **O**  **C**  **C**  **C**  **C**  **C**  **N**  **C**  **O**  **C**  **O**  **C**  **C**  **C**  **O**  **C**  **C**  **N**  **O**  **C**  **C**  **N**  **N**  **S**  **C**  **C**  **C**  **O**  **N**  **C**  **C**  **O**  **N**  **O**  **C**  **C**  **N**  **C**  **C**  **O**  **C**  **O**  **H**  **H**  **H**  **H**  **H**  **H**  **H**  **H**  **H**  **H**  **H**  **H**  **H**  **H**  **H**  **H**  **H**  **H**  **H**  **H**  **H**  **H**  **H**  **H**  **H**  **H**  **H**  **H**  **H**  **H**  **H**  **H**  **H**  **H**  **H**  **H**  **H**  **H**  **H**  **H**  **HN** | **-3.09678** | **6.07385** | **-3.91578** | **-3.39666** | **4.95771** | **-3.78157** | **-3.15457** | **6.10288** | **-3.8626** |  |  |  |  |  |  |
| **C** | **-4.03701** | **5.9293** | **-2.80971** | **-4.15205** | **5.29354** | **-2.57594** | **-4.02624** | **5.96168** | **-2.70921** |  |  |  |  |  |  |
| **C** | **-3.43282** | **5.35711** | **-1.48321** | **-3.41355** | **4.81989** | **-1.27918** | **-3.36781** | **5.3334** | **-1.43869** |  |  |  |  |  |  |
| **C** | **-4.85514** | **7.2124** | **-2.54471** | **-4.592** | **6.77575** | **-2.43963** | **-4.823** | **7.24147** | **-2.35864** |  |  |  |  |  |  |
| **N** | **-4.73047** | **8.25143** | **-3.39814** | **-4.0729** | **7.68446** | **-3.28338** | **-4.66133** | **8.32357** | **-3.14898** |  |  |  |  |  |  |
| **O** | **-5.58781** | **7.24901** | **-1.54122** | **-5.36403** | **7.05256** | **-1.5067** | **-5.57772** | **7.21167** | **-1.37352** |  |  |  |  |  |  |
| **C** | **-5.22449** | **9.62423** | **-3.14722** | **-4.15356** | **9.15221** | **-3.0942** | **-5.16881** | **9.68195** | **-2.85339** |  |  |  |  |  |  |
| **C** | **-6.74517** | **9.74786** | **-2.88822** | **-5.57614** | **9.75234** | **-3.06472** | **-6.69568** | **9.78949** | **-2.62666** |  |  |  |  |  |  |
| **C** | **-4.2974** | **10.26023** | **-2.08253** | **-3.22455** | **9.4587** | **-1.89747** | **-4.27131** | **10.26213** | **-1.737** |  |  |  |  |  |  |
| **C** | **-7.12099** | **11.23199** | **-2.74353** | **-5.48087** | **11.28528** | **-2.96923** | **-7.07753** | **11.26327** | **-2.40972** |  |  |  |  |  |  |
| **C** | **-7.54374** | **9.08458** | **-4.01985** | **-6.37056** | **9.3362** | **-4.31156** | **-7.4648** | **9.19155** | **-3.81364** |  |  |  |  |  |  |
| **N** | **-2.8337** | **7.50872** | **-0.56163** | **-2.68774** | **6.9351** | **-0.28528** | **-2.75903** | **7.39051** | **-0.29582** |  |  |  |  |  |  |
| **C** | **-2.2879** | **8.64885** | **-0.07875** | **-2.00532** | **7.94122** | **0.32055** | **-2.2389** | **8.59558** | **0.05442** |  |  |  |  |  |  |
| **O** | **-1.08764** | **8.81902** | **0.16549** | **-0.82482** | **7.88702** | **0.67467** | **-1.04284** | **8.90249** | **0.04337** |  |  |  |  |  |  |
| **C** | **-3.28513** | **9.82977** | **0.11866** | **-2.81806** | **9.25599** | **0.47847** | **-3.29235** | **9.6782** | **0.43797** |  |  |  |  |  |  |
| **O** | **-0.79604** | **4.99514** | **-2.29418** | **-0.8085** | **4.64444** | **-2.40798** | **-0.72002** | **4.97361** | **-2.22581** |  |  |  |  |  |  |
| **C** | **-1.95073** | **5.55614** | **0.6137** | **-1.61333** | **4.89872** | **0.54193** | **-1.84266** | **5.21589** | **0.53774** |  |  |  |  |  |  |
| **C** | **-1.04971** | **6.07383** | **-1.73598** | **-1.13055** | **5.70262** | **-1.85604** | **-0.98377** | **6.05465** | **-1.67822** |  |  |  |  |  |  |
| **C** | **-2.28181** | **6.15108** | **-0.79407** | **-2.19223** | **5.62294** | **-0.72888** | **-2.19919** | **6.0756** | **-0.70745** |  |  |  |  |  |  |
| **O** | **2.90493** | **7.46036** | **-2.32195** | **2.94087** | **7.35138** | **-2.92546** | **2.98582** | **7.60767** | **-2.51411** |  |  |  |  |  |  |
| **C** | **1.99042** | **6.72636** | **-2.68169** | **1.8761** | **7.32388** | **-2.31551** | **2.04752** | **6.83085** | **-2.661** |  |  |  |  |  |  |
| **C** | **0.60235** | **7.26679** | **-3.09553** | **0.57535** | **6.95919** | **-3.07182** | **0.62289** | **7.30121** | **-3.05735** |  |  |  |  |  |  |
| **N** | **-0.31423** | **7.18094** | **-1.95181** | **-0.58983** | **6.89648** | **-2.19817** | **-0.29405** | **7.18217** | **-1.91818** |  |  |  |  |  |  |
| **O** | **3.89369** | **4.01176** | **0.11877** | **3.47943** | **6.79328** | **2.12763** | **3.78124** | **3.1835** | **-0.22315** |  |  |  |  |  |  |
| **C** | **2.94078** | **4.2604** | **-0.60487** | **2.81958** | **6.61184** | **1.11477** | **3.07205** | **4.11075** | **-0.5853** |  |  |  |  |  |  |
| **C** | **3.15503** | **4.64207** | **-2.07828** | **2.89927** | **7.5816** | **-0.07415** | **3.30317** | **4.8228** | **-1.92494** |  |  |  |  |  |  |
| **N** | **2.06703** | **5.36313** | **-2.70945** | **1.75856** | **7.48612** | **-0.96816** | **2.12589** | **5.48502** | **-2.45525** |  |  |  |  |  |  |
| **N** | **1.63016** | **4.19144** | **-0.16862** | **1.9508** | **5.55507** | **0.95177** | **2.01583** | **4.59324** | **0.15747** |  |  |  |  |  |  |
| **S** | **-1.42101** | **3.77761** | **0.66055** | **-0.33145** | **3.56591** | **0.30509** | **-0.43644** | **5.90733** | **1.54192** |  |  |  |  |  |  |
| **C** | **0.05881** | **3.82282** | **1.65295** | **0.59795** | **3.76058** | **1.82093** | **0.4673** | **4.44048** | **2.00298** |  |  |  |  |  |  |
| **C** | **1.30484** | **3.94872** | **1.15592** | **1.59623** | **4.65935** | **1.94632** | **1.55411** | **4.01691** | **1.32631** |  |  |  |  |  |  |
| **C** | **-3.77027** | **9.89651** | **1.56515** | **-3.55648** | **9.33958** | **1.81037** | **-3.79578** | **9.5168** | **1.87052** |  |  |  |  |  |  |
| **O** | **-4.55684** | **8.72707** | **1.8026** | **-4.58345** | **8.34759** | **1.79565** | **-4.56242** | **8.31087** | **1.92356** |  |  |  |  |  |  |
| **N** | **-4.41216** | **9.78304** | **-0.80779** | **-3.74605** | **9.43875** | **-0.63724** | **-4.4122** | **9.70194** | **-0.49969** |  |  |  |  |  |  |
| **C** | **0.68348** | **8.71078** | **-3.59473** | **0.33936** | **7.83642** | **-4.31994** | **0.64247** | **8.74752** | **-3.56605** |  |  |  |  |  |  |
| **C** | **-0.69979** | **9.32497** | **-3.80422** | **-0.79448** | **7.25175** | **-5.15766** | **-0.75906** | **9.34051** | **-3.68473** |  |  |  |  |  |  |
| **O** | **-1.66587** | **8.66161** | **-4.2051** | **-1.93266** | **7.7375** | **-5.13277** | **-1.72805** | **8.671** | **-4.07367** |  |  |  |  |  |  |
| **N** | **-0.80554** | **10.64694** | **-3.5431** | **-0.51268** | **6.14724** | **-5.88802** | **-0.87854** | **10.64436** | **-3.35509** |  |  |  |  |  |  |
| **O** | **-3.45255** | **11.09913** | **-2.40252** | **-2.00962** | **9.59793** | **-2.07702** | **-3.42204** | **11.11956** | **-1.98871** |  |  |  |  |  |  |
| **C** | **-2.70414** | **5.10764** | **-4.80341** | **-3.8675** | **4.78397** | **-5.04637** | **-3.13963** | **5.35703** | **-5.0096** |  |  |  |  |  |  |
| **C** | **-3.41065** | **3.73308** | **-4.80388** | **-5.37978** | **4.83565** | **-5.28583** | **-4.1981** | **4.26051** | **-5.21702** |  |  |  |  |  |  |
| **N** | **-4.32529** | **3.67458** | **-5.94495** | **-5.59165** | **5.2789** | **-6.65301** | **-4.34863** | **4.09154** | **-6.6531** |  |  |  |  |  |  |
| **C** | **-5.63991** | **4.02901** | **-5.85273** | **-6.70231** | **5.97715** | **-7.02356** | **-5.50843** | **3.64651** | **-7.21139** |  |  |  |  |  |  |
| **C** | **-6.43493** | **3.98831** | **-7.14453** | **-6.76496** | **6.40076** | **-8.47998** | **-5.52344** | **3.55601** | **-8.72732** |  |  |  |  |  |  |
| **O** | **-1.81766** | **5.32653** | **-5.62727** | **-3.1005** | **4.54568** | **-5.98781** | **-2.28527** | **5.55248** | **-5.87415** |  |  |  |  |  |  |
| **C** | **-2.37393** | **2.61048** | **-4.8823** | **-6.00494** | **3.44604** | **-5.03231** | **-3.77948** | **2.94063** | **-4.53616** |  |  |  |  |  |  |
| **O** | **-6.16278** | **4.34857** | **-4.78234** | **-7.6038** | **6.24472** | **-6.22775** | **-6.48671** | **3.33059** | **-6.53157** |  |  |  |  |  |  |
| **H** | **-2.4803** | **6.89055** | **-3.94542** | **-2.40585** | **4.75118** | **-3.66944** | **-2.40862** | **6.8012** | **-3.84311** |  |  |  |  |  |  |
| **H** | **-4.80867** | **5.21681** | **-3.12263** | **-5.09217** | **4.73362** | **-2.57334** | **-4.80936** | **5.24655** | **-2.97436** |  |  |  |  |  |  |
| **H** | **-4.25777** | **5.26308** | **-0.77051** | **-4.16313** | **4.79607** | **-0.48366** | **-4.17515** | **5.18757** | **-0.71434** |  |  |  |  |  |  |
| **H** | **-3.05404** | **4.35581** | **-1.69194** | **-3.0794** | **3.79398** | **-1.44961** | **-2.98673** | **4.35228** | **-1.72155** |  |  |  |  |  |  |
| **H** | **-4.02427** | **8.17795** | **-4.12087** | **-3.36831** | **7.39671** | **-3.96666** | **-3.90502** | **8.29362** | **-3.82675** |  |  |  |  |  |  |
| **H** | **-4.99835** | **10.17698** | **-4.06291** | **-3.62412** | **9.5618** | **-3.95817** | **-4.92221** | **10.27514** | **-3.73802** |  |  |  |  |  |  |
| **H** | **-6.98975** | **9.22917** | **-1.95668** | **-6.10949** | **9.37308** | **-2.18654** | **-6.96262** | **9.22109** | **-1.73092** |  |  |  |  |  |  |
| **H** | **-8.19374** | **11.33094** | **-2.54988** | **-6.48321** | **11.72359** | **-2.93345** | **-8.15545** | **11.35111** | **-2.24139** |  |  |  |  |  |  |
| **H** | **-6.58932** | **11.71609** | **-1.9175** | **-4.9422** | **11.61569** | **-2.07506** | **-6.57025** | **11.69945** | **-1.54282** |  |  |  |  |  |  |
| **H** | **-6.89478** | **11.79011** | **-3.66032** | **-4.96821** | **11.7017** | **-3.84502** | **-6.82635** | **11.87199** | **-3.28685** |  |  |  |  |  |  |
| **H** | **-8.61736** | **9.19058** | **-3.83324** | **-7.36441** | **9.79467** | **-4.29438** | **-8.54297** | **9.28313** | **-3.64758** |  |  |  |  |  |  |
| **H** | **-7.32269** | **8.01749** | **-4.10394** | **-6.5115** | **8.25512** | **-4.3785** | **-7.23841** | **8.13155** | **-3.95514** |  |  |  |  |  |  |
| **H** | **-7.32508** | **9.55743** | **-4.98587** | **-5.86552** | **9.66999** | **-5.22661** | **-7.22473** | **9.71843** | **-4.74568** |  |  |  |  |  |  |
| **H** | **-3.84761** | **7.4935** | **-0.5406** | **-3.69316** | **7.0657** | **-0.35109** | **-3.74911** | **7.30642** | **-0.08878** |  |  |  |  |  |  |
| **H** | **-2.70669** | **10.72961** | **-0.09833** | **-2.06359** | **10.04562** | **0.45022** | **-2.75663** | **10.62514** | **0.35685** |  |  |  |  |  |  |
| **H** | **-2.85365** | **5.65942** | **1.22158** | **-2.46314** | **4.48024** | **1.08904** | **-1.54724** | **4.22048** | **0.20244** |  |  |  |  |  |  |
| **H** | **-1.15902** | **6.15075** | **1.07416** | **-1.13774** | **5.65353** | **1.16411** | **-2.72786** | **5.13549** | **1.17449** |  |  |  |  |  |  |
| **H** | **0.18035** | **6.63096** | **-3.87914** | **0.75346** | **5.92886** | **-3.39414** | **0.23922** | **6.63615** | **-3.83713** |  |  |  |  |  |  |
| **H** | **-0.38339** | **7.95393** | **-1.28544** | **-1.02649** | **7.77175** | **-1.91784** | **-0.39563** | **7.96029** | **-1.25361** |  |  |  |  |  |  |
| **H** | **4.05288** | **5.26108** | **-2.10775** | **3.00146** | **8.58478** | **0.35176** | **4.08359** | **5.57959** | **-1.79585** |  |  |  |  |  |  |
| **H** | **3.36132** | **3.72188** | **-2.63597** | **3.8038** | **7.37367** | **-0.65405** | **3.68254** | **4.06509** | **-2.61702** |  |  |  |  |  |  |
| **H** | **1.2094** | **4.87283** | **-2.94612** | **0.83485** | **7.59766** | **-0.55677** | **1.26839** | **4.94995** | **-2.58902** |  |  |  |  |  |  |
| **H** | **0.867** | **4.32961** | **-0.82557** | **1.46212** | **5.4656** | **0.06771** | **1.49459** | **5.38606** | **-0.20018** |  |  |  |  |  |  |
| **H** | **-0.05797** | **3.65292** | **2.71803** | **0.37946** | **3.08793** | **2.64299** | **0.17361** | **3.92789** | **2.91241** |  |  |  |  |  |  |
| **H** | **2.17145** | **3.88228** | **1.80627** | **2.17268** | **4.75424** | **2.86098** | **2.13559** | **3.16311** | **1.65977** |  |  |  |  |  |  |
| **H** | **-2.90119** | **9.93573** | **2.23405** | **-2.84021** | **9.16679** | **2.62352** | **-2.93821** | **9.47347** | **2.55409** |  |  |  |  |  |  |
| **H** | **-4.36829** | **10.80753** | **1.69494** | **-3.9824** | **10.34586** | **1.91866** | **-4.41601** | **10.38591** | **2.12435** |  |  |  |  |  |  |
| **H** | **-4.96775** | **8.77759** | **2.67421** | **-5.11902** | **8.41287** | **2.59578** | **-4.95684** | **8.2066** | **2.79784** |  |  |  |  |  |  |
| **H** | **-5.12432** | **9.07573** | **-0.6448** | **-4.69168** | **9.08663** | **-0.51908** | **-5.09713** | **8.9531** | **-0.41709** |  |  |  |  |  |  |
| **H** | **1.21009** | **8.71708** | **-4.5563** | **1.278** | **7.87485** | **-4.88102** | **1.10706** | **8.76166** | **-4.55843** |  |  |  |  |  |  |
| **H** | **1.28739** | **9.30761** | **-2.90454** | **0.0533** | **8.85087** | **-4.03248** | **1.27923** | **9.3557** | **-2.91756** |  |  |  |  |  |  |
| **H** | **-1.74141** | **11.03989** | **-3.46954** | **-1.29605** | **5.59191** | **-6.22861** | **-1.81437** | **11.01738** | **-3.20763** |  |  |  |  |  |  |
| **H** | **-0.05199** | **11.1395** | **-3.08803** | **0.3939** | **5.70662** | **-5.83621** | **-0.11674** | **11.12139** | **-2.89682** |  |  |  |  |  |  |
| **H** | **-4.02972** | **3.59563** | **-3.91828** | **-5.86435** | **5.56638** | **-4.63294** | **-5.17555** | **4.56592** | **-4.83078** |  |  |  |  |  |  |
| **H** | **-3.91106** | **3.55589** | **-6.85955** | **-4.83364** | **5.10495** | **-7.3009** | **-3.56421** | **4.39808** | **-7.21617** |  |  |  |  |  |  |
| **H** | **-7.29751** | **3.3317** | **-7.00237** | **-7.65367** | **5.95558** | **-8.93646** | **-6.35604** | **4.15576** | **-9.10543** |  |  |  |  |  |  |
| **H** | **-6.81646** | **4.99195** | **-7.35369** | **-5.88276** | **6.1115** | **-9.05754** | **-4.59445** | **3.89456** | **-9.19425** |  |  |  |  |  |  |
| **H** | **-5.85618** | **3.63964** | **-8.00413** | **-6.88253** | **7.48705** | **-8.5236** | **-5.71021** | **2.51719** | **-9.01445** |  |  |  |  |  |  |
| **H** | **-2.87708** | **1.63944** | **-4.90125** | **-7.07801** | **3.49984** | **-5.23067** | **-4.52435** | **2.17471** | **-4.76491** |  |  |  |  |  |  |
| **H** | **-1.71338** | **2.65056** | **-4.01169** | **-5.86097** | **3.1186** | **-3.99726** | **-3.71542** | **3.04492** | **-3.44892** |  |  |  |  |  |  |
| **H** | **-1.75468** | **2.72038** | **-5.77699** | **-5.55393** | **2.70208** | **-5.69549** | **-2.80315** | **2.61584** | **-4.90749** |  |  |  |  |  |  |

# Table S7. Lowest-energy Conformers Optimized at the M062X/6-311+G (d, p) Level of **1b** with Relative Energies < 3.0 kcal/mol

| **1b_C1** | G 2697.1377 Ha  Boltzman pop. 47.10% | **1b_C2** | G 2697.1378 Ha  Boltzman pop. 47.11% | **1b_C3** | G 2697.1377 Ha  Boltzman pop. 5.79% |
| --- | --- | --- | --- | --- | --- |
| 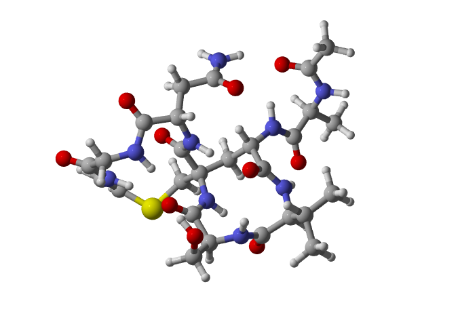 | | 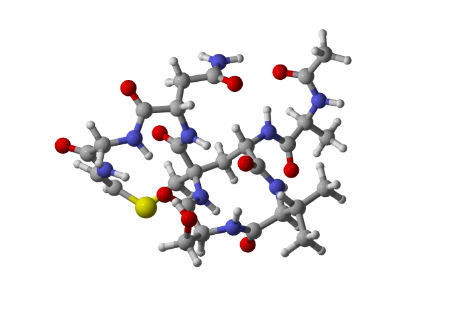 | | 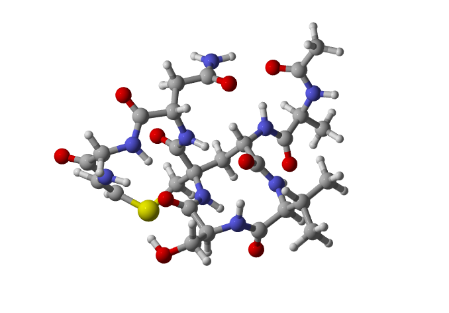 | |

# Table S8. Atomic Coordinates for the Lowest-energy Conformers of **1b** (**1b_C1**–**1b_C3**)

| Atoms | **1b_C1** | | | **1b_C2** | | | **1b_C3** | | |
| --- | --- | --- | --- | --- | --- | --- | --- | --- | --- |
|  | x | y | z | x | y | z | x | y | z |
| N | -3.05641 | 1.96059 | -1.83524 | -3.05983 | 1.95991 | -1.84925 | -3.01814 | 1.95781 | -1.87847 |
| C | -4.25721 | 1.81296 | -1.00296 | -4.25879 | 1.81235 | -1.0143 | -4.21425 | 1.84896 | -1.03417 |
| C | -5.1292 | 0.61364 | -1.48729 | -5.13335 | 0.61493 | -1.49876 | -5.1113 | 0.65793 | -1.4898 |
| C | -4.03508 | 1.81725 | 0.52725 | -4.03308 | 1.81357 | 0.51539 | -3.97054 | 1.87953 | 0.49254 |
| N | -3.12071 | 0.96319 | 1.03867 | -3.11838 | 0.95767 | 1.02309 | -3.11321 | 0.97661 | 1.01167 |
| O | -4.75946 | 2.54108 | 1.22611 | -4.75503 | 2.53692 | 1.21727 | -4.63751 | 2.67243 | 1.1792 |
| C | -3.09419 | 0.53482 | 2.45483 | -3.08902 | 0.52665 | 2.43839 | -2.98874 | 0.63588 | 2.44728 |
| C | -2.74587 | 1.63874 | 3.47678 | -2.73706 | 1.6283 | 3.46156 | -2.54135 | 1.79313 | 3.3696 |
| C | -4.41812 | -0.24524 | 2.63736 | -4.41341 | -0.25223 | 2.62258 | -4.30241 | -0.09692 | 2.81945 |
| C | -2.61835 | 1.02503 | 4.88145 | -2.60703 | 1.0119 | 4.86481 | -2.36585 | 1.26771 | 4.804 |
| C | -1.44908 | 2.35941 | 3.07759 | -1.44034 | 2.34819 | 3.06072 | -1.24166 | 2.42899 | 2.85424 |
| N | -6.52264 | 0.14202 | 0.48139 | -6.52265 | 0.14121 | 0.47236 | -6.4674 | 0.28333 | 0.51969 |
| C | -7.35473 | 0.4456 | 1.503 | -7.35201 | 0.44378 | 1.49645 | -7.29733 | 0.60154 | 1.54181 |
| O | -8.39869 | 1.10542 | 1.40499 | -8.39556 | 1.1048 | 1.40206 | -8.37129 | 1.20569 | 1.41818 |
| C | -6.85588 | -0.04969 | 2.89142 | -6.85036 | -0.05441 | 2.88281 | -6.752 | 0.21016 | 2.94243 |
| O | -8.10037 | 1.82845 | -2.27496 | -8.10471 | 1.83452 | -2.27737 | -8.07413 | 1.87738 | -2.27092 |
| C | -7.24516 | -0.70945 | -1.72693 | -7.25121 | -0.70541 | -1.7358 | -7.23758 | -0.65797 | -1.64807 |
| C | -7.35982 | 1.80293 | -1.29286 | -7.36208 | 1.80634 | -1.2969 | -7.32562 | 1.87067 | -1.29567 |
| C | -6.59948 | 0.47898 | -0.95235 | -6.60248 | 0.48092 | -0.96057 | -6.57183 | 0.55186 | -0.92643 |
| O | -9.8615 | 5.08194 | -1.39087 | -9.86084 | 5.08784 | -1.38293 | -9.80853 | 5.14123 | -1.4714 |
| C | -9.19768 | 4.27598 | -0.74709 | -9.19637 | 4.27999 | -0.74216 | -9.14135 | 4.36502 | -0.79538 |
| C | -7.65432 | 4.22581 | -0.88943 | -7.65339 | 4.22867 | -0.88797 | -7.59929 | 4.30501 | -0.94804 |
| N | -7.09538 | 2.90993 | -0.55458 | -7.09494 | 2.91161 | -0.55701 | -7.04694 | 2.99341 | -0.58542 |
| O | -12.73046 | 2.10701 | -1.25866 | -12.73257 | 2.11528 | -1.24977 | -12.69604 | 2.21513 | -1.18676 |
| C | -11.65244 | 2.04099 | -0.6865 | -11.65325 | 2.04718 | -0.68026 | -11.6087 | 2.1611 | -0.63154 |
| C | -11.15902 | 3.1698 | 0.2367 | -11.15662 | 3.17385 | 0.24378 | -11.09809 | 3.31346 | 0.25293 |
| N | -9.72806 | 3.41724 | 0.16948 | -9.72557 | 3.41991 | 0.17382 | -9.66671 | 3.55041 | 0.16266 |
| N | -10.80211 | 0.95844 | -0.80881 | -10.8043 | 0.96403 | -0.80656 | -10.76303 | 1.07323 | -0.7388 |
| S | -8.74843 | -1.45938 | -0.94307 | -8.75326 | -1.45547 | -0.94976 | -8.73598 | -1.37368 | -0.82624 |
| C | -10.11428 | -1.03816 | -2.0049 | -10.1213 | -1.03122 | -2.00755 | -10.11141 | -0.96678 | -1.88163 |
| C | -10.95656 | -0.01022 | -1.78122 | -10.96209 | -0.00288 | -1.78017 | -10.94064 | 0.07577 | -1.67769 |
| C | -7.78401 | 0.42415 | 4.0119 | -7.77522 | 0.41875 | 4.00628 | -7.64949 | 0.75381 | 4.06575 |
| O | -7.77685 | 1.84027 | 4.14599 | -7.76606 | 1.83464 | 4.14266 | -8.95054 | 0.20038 | 4.02813 |
| N | -5.49116 | 0.42428 | 3.12879 | -5.48453 | 0.4176 | 3.11764 | -5.38047 | 0.70233 | 3.08812 |
| C | -7.25345 | 4.74535 | -2.28136 | -7.25502 | 4.75069 | -2.27965 | -7.20557 | 4.78636 | -2.35568 |
| C | -5.74486 | 4.83661 | -2.45983 | -5.74675 | 4.84121 | -2.46094 | -5.69779 | 4.84678 | -2.55274 |
| O | -4.97035 | 5.01462 | -1.51505 | -4.97034 | 5.01736 | -1.51736 | -4.90921 | 5.026 | -1.61962 |
| N | -5.29162 | 4.69892 | -3.73516 | -5.29605 | 4.70495 | -3.73731 | -5.26111 | 4.67786 | -3.82994 |
| O | -4.50268 | -1.39918 | 2.20015 | -4.50026 | -1.40533 | 2.18354 | -4.37226 | -1.32244 | 2.75108 |
| C | -2.02635 | 1.10026 | -1.98131 | -2.03109 | 1.09864 | -1.99919 | -2.01265 | 1.06821 | -2.01806 |
| C | -1.00467 | 1.5068 | -3.07878 | -1.01158 | 1.506 | -3.09837 | -0.99816 | 1.41882 | -3.14056 |
| N | -0.51441 | 2.8815 | -2.90412 | -0.51864 | 2.87949 | -2.92159 | -0.47771 | 2.78829 | -3.01925 |
| C | -1.16365 | 3.98485 | -3.36043 | -1.16719 | 3.98495 | -3.37384 | -1.1132 | 3.89053 | -3.49782 |
| C | -0.46896 | 5.31554 | -3.1821 | -0.46982 | 5.31408 | -3.19426 | -0.38135 | 5.2087 | -3.38913 |
| O | -1.88651 | 0.06056 | -1.32511 | -1.89093 | 0.05764 | -1.34513 | -1.89186 | 0.04073 | -1.33812 |
| C | 0.17122 | 0.53626 | -3.13657 | 0.16247 | 0.53362 | -3.16152 | 0.15734 | 0.4232 | -3.18274 |
| O | -2.2852 | 3.90077 | -3.88402 | -2.29009 | 3.90393 | -3.89501 | -2.25132 | 3.81592 | -3.98585 |
| H | -3.07702 | 2.75159 | -2.47697 | -3.08073 | 2.75228 | -2.48927 | -3.03116 | 2.73207 | -2.54126 |
| H | -4.79095 | 2.74252 | -1.17709 | -4.7919 | 2.74283 | -1.18553 | -4.7315 | 2.78615 | -1.21878 |
| H | -5.19628 | 0.7004 | -2.57679 | -5.20289 | 0.7038 | -2.58793 | -5.19666 | 0.73138 | -2.57879 |
| H | -4.60339 | -0.32454 | -1.27778 | -4.60808 | -0.32422 | -1.29225 | -4.5952 | -0.28559 | -1.2792 |
| H | -2.62645 | 0.37695 | 0.36204 | -2.62635 | 0.37214 | 0.34423 | -2.62658 | 0.38005 | 0.33709 |
| H | -2.3132 | -0.22978 | 2.50229 | -2.30877 | -0.23891 | 2.48258 | -2.22261 | -0.1431 | 2.48603 |
| H | -3.5462 | 2.38653 | 3.48428 | -3.53651 | 2.37699 | 3.47227 | -3.31324 | 2.56955 | 3.37088 |
| H | -2.39391 | 1.80393 | 5.61691 | -2.38012 | 1.78923 | 5.60118 | -2.06552 | 2.08144 | 5.47158 |
| H | -3.53753 | 0.52382 | 5.20012 | -3.526 | 0.51105 | 5.18465 | -3.29053 | 0.83712 | 5.20101 |
| H | -1.8039 | 0.29072 | 4.91585 | -1.79325 | 0.27669 | 4.89605 | -1.58811 | 0.49553 | 4.84895 |
| H | -1.54217 | 2.84809 | 2.10409 | -1.53507 | 2.83873 | 2.08832 | -1.36634 | 2.85123 | 1.85317 |
| H | -0.60676 | 1.65743 | 3.0297 | -0.59898 | 1.6453 | 3.00966 | -0.4298 | 1.69149 | 2.81696 |
| H | -1.20139 | 3.12688 | 3.81794 | -1.19008 | 3.11405 | 3.80188 | -0.92661 | 3.23738 | 3.52191 |
| H | -5.7918 | -0.52697 | 0.7152 | -5.79185 | -0.52885 | 0.70324 | -5.69329 | -0.32021 | 0.77341 |
| H | -6.82427 | -1.14456 | 2.87889 | -6.82003 | -1.1493 | 2.86841 | -6.71798 | -0.88178 | 3.01317 |
| H | -6.50992 | -1.5181 | -1.77781 | -6.5169 | -1.51469 | -1.79004 | -6.50806 | -1.47289 | -1.68002 |
| H | -7.50177 | -0.39545 | -2.73826 | -7.50995 | -0.38926 | -2.74592 | -7.50147 | -0.37649 | -2.66725 |
| H | -7.23609 | 4.90956 | -0.14371 | -7.23296 | 4.91056 | -0.14177 | -7.17114 | 5.00359 | -0.22209 |
| H | -6.4061 | 2.87122 | 0.1974 | -6.40388 | 2.87078 | 0.1932 | -6.34541 | 2.97177 | 0.15342 |
| H | -11.69215 | 4.07158 | -0.06432 | -11.68944 | 4.0767 | -0.05458 | -11.63071 | 4.2086 | -0.06823 |
| H | -11.43773 | 2.92829 | 1.26887 | -11.4334 | 2.93094 | 1.27614 | -11.36518 | 3.10469 | 1.29509 |
| H | -9.10621 | 2.74281 | 0.60294 | -9.10341 | 2.7441 | 0.60469 | -9.0449 | 2.90303 | 0.63506 |
| H | -9.95326 | 0.92434 | -0.25201 | -9.95424 | 0.92799 | -0.25171 | -9.90365 | 1.05255 | -0.19741 |
| H | -10.32828 | -1.72073 | -2.82011 | -10.33795 | -1.71232 | -2.82329 | -10.345 | -1.67216 | -2.67167 |
| H | -11.83349 | 0.13536 | -2.40429 | -11.84042 | 0.1445 | -2.40083 | -11.82587 | 0.20949 | -2.29165 |
| H | -7.42263 | 0.01976 | 4.9608 | -7.41213 | 0.01249 | 4.95372 | -7.67846 | 1.85281 | 4.02092 |
| H | -8.79604 | 0.04389 | 3.83093 | -8.78812 | 0.03998 | 3.82715 | -7.20685 | 0.46471 | 5.02223 |
| H | -8.261 | 2.1946 | 3.38537 | -8.25169 | 2.19084 | 3.38387 | -9.36327 | 0.50701 | 3.20662 |
| H | -5.40359 | 1.424 | 3.27242 | -5.39554 | 1.41692 | 3.26318 | -5.24317 | 1.6873 | 2.87542 |
| H | -7.68253 | 5.74647 | -2.39607 | -7.68353 | 5.7524 | -2.39139 | -7.61895 | 5.79177 | -2.48907 |
| H | -7.70382 | 4.11864 | -3.05403 | -7.70755 | 4.12598 | -3.05267 | -7.67545 | 4.15064 | -3.10904 |
| H | -4.29239 | 4.73247 | -3.91301 | -4.29719 | 4.73829 | -3.91709 | -4.26368 | 4.6932 | -4.01914 |
| H | -5.92118 | 4.51972 | -4.50207 | -5.9272 | 4.52731 | -4.50328 | -5.90209 | 4.5016 | -4.58808 |
| H | -1.54784 | 1.49608 | -4.03141 | -1.5572 | 1.49859 | -4.04963 | -1.55598 | 1.38655 | -4.08444 |
| H | 0.37734 | 2.99972 | -2.44303 | 0.37445 | 2.99519 | -2.46247 | 0.43227 | 2.90051 | -2.59363 |
| H | 0.52969 | 5.2325 | -2.7459 | -0.39766 | 5.80472 | -4.16879 | 0.62418 | 5.11762 | -2.97076 |
| H | -1.09039 | 5.94847 | -2.54168 | 0.52967 | 5.2284 | -2.76047 | -0.97235 | 5.88384 | -2.76331 |
| H | -0.39548 | 5.80428 | -4.15749 | -1.08871 | 5.94674 | -2.55114 | -0.31658 | 5.65466 | -4.38547 |
| H | 0.82977 | 0.79609 | -3.97022 | 0.81936 | 0.79436 | -3.9962 | 0.80795 | 0.64159 | -4.03428 |
| H | -0.18937 | -0.4844 | -3.27316 | -0.2002 | -0.48609 | -3.29973 | -0.22526 | -0.59389 | -3.27992 |
| H | 0.74507 | 0.55216 | -2.20424 | 0.73868 | 0.54623 | -2.23061 | 0.74579 | 0.45873 | -2.26009 |

# Table S9. Lowest-energy Conformers Optimized at the M062X/6-311+G (d, p) Level of **1c** with Relative Energies < 3.0 kcal/mol

| **1c_C1** | G 2697.1415 Ha  Boltzman pop. 35.15% | **1c_C2** | G 2697.1411 Ha  Boltzman pop. 30.52% | **1c_C3** | G 2697.1411 Ha  Boltzman pop. 30.47% |
| --- | --- | --- | --- | --- | --- |
| 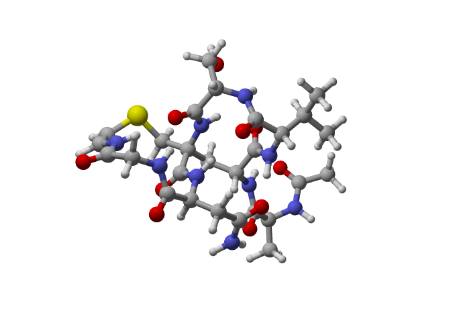 | | 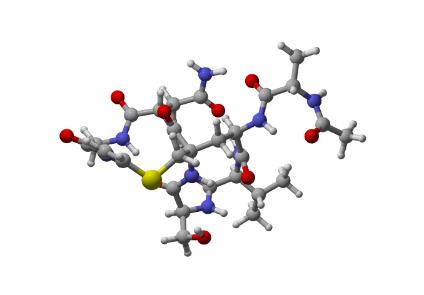 | | 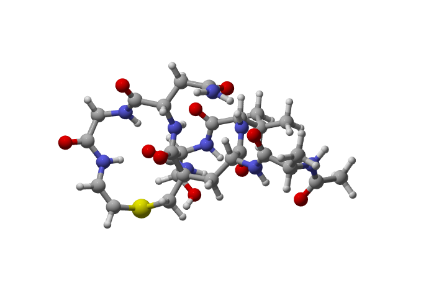 | |
| **1c_C4** | G 2697.1401 Ha  Boltzman pop. 2.65% | **1c_C5** | G 2697.1413 Ha  Boltzman pop. 1.21% |  | |
| 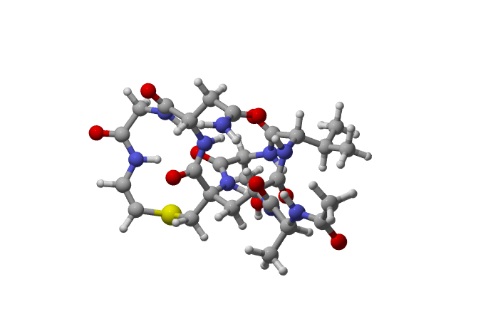 | | 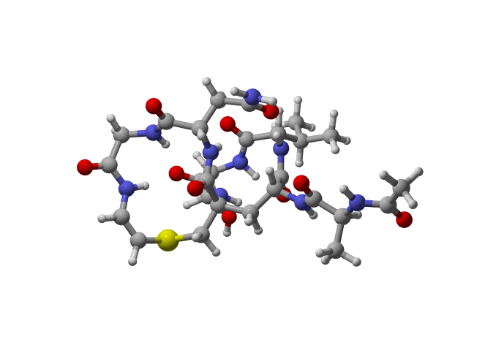 | |  |  |

# Table S10. Atomic Coordinates for the Lowest-energy Conformers of **1c** (**1c_C1**–**1c_C5**)

| **Atoms** | **1c_C1** | | | **1c_C2** | | | **1c_C3** | | | **1c_C4** | | | **1c_C5** | | |
| --- | --- | --- | --- | --- | --- | --- | --- | --- | --- | --- | --- | --- | --- | --- | --- |
|  | **x** | **y** | **z** | **x** | **y** | **z** | **x** | **y** | **z** | **x** | **y** | **z** | **x** | **y** | **z** |
| **N** | **4.23674** | **1.38727** | **-8.54054** | **4.22773** | **1.38258** | **-8.5563** | **4.23483** | **1.37414** | **-8.51607** | **4.01719** | **1.12006** | **-8.09837** | **3.90646** | **0.99512** | **-8.03503** |
| **C** | **3.29626** | **2.31857** | **-7.91971** | **3.27983** | **2.30333** | **-7.93227** | **3.27937** | **2.29888** | **-7.90956** | **3.12415** | **2.15985** | **-7.58483** | **3.04712** | **2.09904** | **-7.6078** |
| **C** | **4.01364** | **3.0643** | **-6.76596** | **3.98833** | **3.04355** | **-6.76958** | **3.97795** | **3.05767** | **-6.75287** | **3.86317** | **2.96486** | **-6.48516** | **3.76915** | **2.88197** | **-6.47858** |
| **C** | **2.07537** | **1.53378** | **-7.39096** | **2.06223** | **1.51126** | **-7.40752** | **2.0624** | **1.50826** | **-7.38128** | **1.86625** | **1.46967** | **-7.01658** | **1.72899** | **1.4819** | **-7.10822** |
| **N** | **0.94434** | **1.63713** | **-8.12013** | **0.93608** | **1.58884** | **-8.14514** | **0.94023** | **1.57078** | **-8.12639** | **0.7433** | **1.58639** | **-7.75019** | **0.64082** | **1.69995** | **-7.87131** |
| **O** | **2.16011** | **0.89529** | **-6.33084** | **2.14198** | **0.89552** | **-6.33211** | **2.13871** | **0.90712** | **-6.2974** | **1.93611** | **0.87929** | **-5.92643** | **1.72097** | **0.84625** | **-6.04077** |
| **C** | **-0.40608** | **1.34981** | **-7.59241** | **-0.41221** | **1.30442** | **-7.60718** | **-0.40989** | **1.28708** | **-7.59262** | **-0.62257** | **1.37973** | **-7.22316** | **-0.75047** | **1.52731** | **-7.40368** |
| **C** | **-0.6682** | **-0.10632** | **-7.15364** | **-0.66804** | **-0.14592** | **-7.14703** | **-0.66162** | **-0.15838** | **-7.11518** | **-0.93865** | **-0.03053** | **-6.68296** | **-1.14684** | **0.10748** | **-6.94733** |
| **C** | **-0.64771** | **2.47903** | **-6.56424** | **-0.64923** | **2.44959** | **-6.59561** | **-0.65812** | **2.44413** | **-6.59744** | **-0.82941** | **2.59132** | **-6.28614** | **-0.94167** | **2.69849** | **-6.41498** |
| **C** | **-2.14689** | **-0.2713** | **-6.76326** | **-2.14648** | **-0.3118** | **-6.75551** | **-2.14142** | **-0.32613** | **-6.7297** | **-2.42567** | **-0.11339** | **-6.29769** | **-2.6485** | **0.07422** | **-6.61541** |
| **C** | **-0.28822** | **-1.08518** | **-8.27484** | **-0.28343** | **-1.13994** | **-8.25333** | **-0.26633** | **-1.16471** | **-8.20649** | **-0.58488** | **-1.10145** | **-7.72597** | **-0.80421** | **-0.92462** | **-8.03227** |
| **N** | **2.08211** | **3.68108** | **-5.36336** | **2.0472** | **3.65696** | **-5.36401** | **2.02602** | **3.68056** | **-5.36679** | **1.94301** | **3.763** | **-5.1624** | **1.85956** | **3.70826** | **-5.16318** |
| **C** | **0.99122** | **4.32437** | **-4.88306** | **0.9521** | **4.31151** | **-4.90077** | **0.9252** | **4.33576** | **-4.91837** | **0.87637** | **4.48793** | **-4.74408** | **0.80655** | **4.45351** | **-4.74772** |
| **O** | **0.82234** | **5.54761** | **-4.87192** | **0.81079** | **5.53769** | **-4.86989** | **0.77782** | **5.56156** | **-4.90443** | **0.7585** | **5.71295** | **-4.83609** | **0.73159** | **5.68439** | **-4.80124** |
| **C** | **-0.1452** | **3.3972** | **-4.37505** | **-0.20066** | **3.40181** | **-4.41322** | **-0.22591** | **3.42688** | **-4.4253** | **-0.29776** | **3.65164** | **-4.16948** | **-0.41354** | **3.63948** | **-4.23886** |
| **O** | **3.87513** | **6.13277** | **-7.34885** | **3.85179** | **6.11989** | **-7.32544** | **3.83028** | **6.1251** | **-7.35163** | **3.87496** | **5.97541** | **-7.29554** | **3.82117** | **5.92694** | **-7.25815** |
| **C** | **4.34338** | **4.72701** | **-4.96368** | **4.31344** | **4.69488** | **-4.95643** | **4.28468** | **4.73536** | **-4.96048** | **4.24084** | **4.74165** | **-4.80267** | **4.18389** | **4.6346** | **-4.78779** |
| **C** | **2.9873** | **5.33776** | **-7.0541** | **2.96414** | **5.31909** | **-7.04591** | **2.94461** | **5.32431** | **-7.06591** | **2.94479** | **5.24986** | **-6.95626** | **2.89177** | **5.19479** | **-6.93444** |
| **C** | **3.28726** | **4.231** | **-6.00407** | **3.2583** | **4.20393** | **-6.00113** | **3.23808** | **4.22489** | **-6.00436** | **3.17905** | **4.21354** | **-5.82163** | **3.1097** | **4.13985** | **-5.81127** |
| **O** | **0.88723** | **8.6321** | **-8.91147** | **0.92417** | **8.6304** | **-8.90763** | **0.89766** | **8.60086** | **-8.98208** | **0.99249** | **8.47599** | **-9.10044** | **1.10224** | **8.49069** | **-9.01331** |
| **C** | **0.90619** | **7.64781** | **-8.17772** | **0.93169** | **7.64523** | **-8.17519** | **0.90626** | **7.62547** | **-8.23667** | **0.96778** | **7.5533** | **-8.29117** | **1.03404** | **7.56219** | **-8.21198** |
| **C** | **1.48852** | **6.32089** | **-8.72309** | **1.4859** | **6.30809** | **-8.72593** | **1.47077** | **6.28425** | **-8.7666** | **1.51815** | **6.17108** | **-8.7197** | **1.45687** | **6.14538** | **-8.67868** |
| **N** | **1.78642** | **5.31485** | **-7.7069** | **1.77082** | **5.29718** | **-7.71059** | **1.75494** | **5.28809** | **-7.73653** | **1.75298** | **5.23309** | **-7.62493** | **1.70195** | **5.18906** | **-7.60592** |
| **O** | **1.91138** | **10.42262** | **-5.20672** | **1.96545** | **10.37334** | **-5.11556** | **1.90892** | **10.39903** | **-5.20814** | **2.0529** | **10.50255** | **-5.52852** | **2.23394** | **10.44385** | **-5.36735** |
| **C** | **1.57627** | **9.25334** | **-5.32905** | **1.61461** | **9.21306** | **-5.27217** | **1.56458** | **9.23503** | **-5.35089** | **1.66849** | **9.34305** | **-5.56417** | **1.812** | **9.29878** | **-5.43734** |
| **C** | **0.31245** | **8.85466** | **-6.11453** | **0.36768** | **8.85626** | **-6.10319** | **0.32421** | **8.86069** | **-6.18401** | **0.39752** | **8.93724** | **-6.33468** | **0.54405** | **8.95656** | **-6.24099** |
| **N** | **0.48004** | **7.63524** | **-6.88791** | **0.51866** | **7.63802** | **-6.88098** | **0.48599** | **7.63312** | **-6.94479** | **0.52509** | **7.65649** | **-7.01103** | **0.62211** | **7.67635** | **-6.92484** |
| **N** | **2.29366** | **8.20327** | **-4.78626** | **2.29513** | **8.14065** | **-4.72307** | **2.2472** | **8.17354** | **-4.78346** | **2.33466** | **8.30879** | **-4.93148** | **2.42521** | **8.22971** | **-4.81177** |
| **S** | **3.77524** | **5.69842** | **-3.495** | **3.72827** | **5.58133** | **-3.44115** | **3.68629** | **5.63841** | **-3.46029** | **3.69471** | **5.85266** | **-3.42787** | **3.65357** | **5.68563** | **-3.35973** |
| **C** | **4.33749** | **7.36492** | **-3.78093** | **4.28162** | **7.26499** | **-3.63159** | **4.23197** | **7.32234** | **-3.66967** | **4.33091** | **7.46593** | **-3.83737** | **4.35349** | **7.29334** | **-3.67814** |
| **C** | **3.56945** | **8.35489** | **-4.2772** | **3.54106** | **8.26926** | **-4.14184** | **3.48918** | **8.31607** | **-4.19707** | **3.61055** | **8.44543** | **-4.41889** | **3.68606** | **8.31328** | **-4.25262** |
| **C** | **0.07995** | **2.9265** | **-2.93968** | **-0.03233** | **2.96295** | **-2.95128** | **-0.06365** | **3.00792** | **-2.95686** | **-0.09194** | **3.28187** | **-2.70202** | **-0.26022** | **3.19132** | **-2.78705** |
| **O** | **1.15245** | **1.98284** | **-2.96143** | **1.02618** | **2.02357** | **-2.78199** | **0.99812** | **2.07563** | **-2.76963** | **0.94124** | **2.29573** | **-2.64931** | **0.73258** | **2.16394** | **-2.7588** |
| **N** | **-0.34533** | **2.24692** | **-5.2548** | **-0.37614** | **2.23748** | **-5.27709** | **-0.39101** | **2.25057** | **-5.27482** | **-0.54459** | **2.44732** | **-4.96044** | **-0.68844** | **2.48466** | **-5.09366** |
| **C** | **0.61527** | **5.73839** | **-9.85338** | **0.5978** | **5.73765** | **-9.85071** | **0.59257** | **5.69464** | **-9.88926** | **0.66331** | **5.53271** | **-9.83374** | **0.44049** | **5.59034** | **-9.70127** |
| **C** | **1.28018** | **4.51972** | **-10.48183** | **1.248** | **4.51371** | **-10.48561** | **1.25275** | **4.46557** | **-10.50359** | **1.31046** | **4.24894** | **-10.33709** | **1.0372** | **4.4599** | **-10.52513** |
| **O** | **0.80871** | **3.38215** | **-10.33997** | **0.77601** | **3.37826** | **-10.33155** | **0.78538** | **3.32995** | **-10.3373** | **0.81087** | **3.13834** | **-10.10651** | **0.89755** | **3.26695** | **-10.22729** |
| **N** | **2.42817** | **4.72514** | **-11.16654** | **2.38564** | **4.71377** | **-11.18917** | **2.39388** | **4.66187** | **-11.2025** | **2.47978** | **4.36613** | **-11.00659** | **1.75989** | **4.83092** | **-11.60898** |
| **O** | **-0.97955** | **3.60096** | **-6.95638** | **-0.95007** | **3.5728** | **-7.01093** | **-0.96228** | **3.56029** | **-7.02901** | **-1.10887** | **3.69298** | **-6.76597** | **-1.17702** | **3.82998** | **-6.85113** |
| **C** | **4.64689** | **1.49846** | **-9.83111** | **4.62158** | **1.49049** | **-9.85271** | **4.63552** | **1.46654** | **-9.81158** | **4.5378** | **1.11829** | **-9.3539** | **4.66262** | **1.03196** | **-9.16702** |
| **C** | **5.6301** | **0.38798** | **-10.28328** | **5.61919** | **0.39346** | **-10.30637** | **5.63946** | **0.36722** | **-10.24529** | **5.49749** | **-0.0382** | **-9.65966** | **5.5596** | **-0.19429** | **-9.39173** |
| **N** | **4.97318** | **-0.92361** | **-10.30649** | **4.98935** | **-0.93172** | **-10.30182** | **5.01406** | **-0.95992** | **-10.22705** | **5.4757** | **-0.26249** | **-11.09401** | **5.90387** | **-0.2255** | **-10.80149** |
| **C** | **4.86728** | **-1.71774** | **-9.20407** | **4.91396** | **-1.71177** | **-9.18692** | **4.93521** | **-1.7258** | **-9.10264** | **5.74546** | **-1.48542** | **-11.63743** | **6.24159** | **-1.38211** | **-11.43949** |
| **C** | **4.27454** | **-3.09597** | **-9.4127** | **4.35583** | **-3.10787** | **-9.36896** | **4.38302** | **-3.12613** | **-9.26971** | **5.64856** | **-1.57697** | **-13.14952** | **6.52928** | **-1.26168** | **-12.92569** |
| **O** | **4.29661** | **2.40084** | **-10.59454** | **4.24782** | **2.38119** | **-10.61828** | **4.26278** | **2.34567** | **-10.5909** | **4.30198** | **1.99404** | **-10.19135** | **4.65937** | **1.98343** | **-9.94767** |
| **C** | **6.23791** | **0.6983** | **-11.64751** | **6.19873** | **0.69754** | **-11.68422** | **6.22578** | **0.65541** | **-11.6237** | **6.91619** | **0.28029** | **-9.14241** | **6.81166** | **-0.13648** | **-8.49166** |
| **O** | **5.23913** | **-1.3314** | **-8.09105** | **5.28551** | **-1.29861** | **-8.08326** | **5.29938** | **-1.29721** | **-8.0024** | **6.05106** | **-2.45443** | **-10.94294** | **6.31411** | **-2.45938** | **-10.84742** |
| **H** | **4.56665** | **0.58229** | **-8.00556** | **4.57827** | **0.587** | **-8.01979** | **4.58527** | **0.58696** | **-7.96725** | **4.22011** | **0.34139** | **-7.48388** | **3.9498** | **0.18374** | **-7.43125** |
| **H** | **2.99295** | **3.01251** | **-8.70296** | **2.97313** | **3.00008** | **-8.71188** | **2.974** | **2.98387** | **-8.7** | **2.87246** | **2.79167** | **-8.43541** | **2.88521** | **2.72619** | **-8.48519** |
| **H** | **4.93161** | **3.48928** | **-7.18038** | **4.90658** | **3.47572** | **-7.17596** | **4.89659** | **3.48851** | **-7.15976** | **4.80133** | **3.31923** | **-6.92047** | **4.72826** | **3.21175** | **-6.88695** |
| **H** | **4.30276** | **2.31412** | **-6.02191** | **4.27705** | **2.28835** | **-6.03075** | **4.26573** | **2.31378** | **-6.00229** | **4.1143** | **2.26332** | **-5.68193** | **3.98081** | **2.16488** | **-5.67764** |
| **H** | **0.95544** | **2.24066** | **-8.94899** | **0.94217** | **2.19274** | **-8.97405** | **0.94843** | **2.16377** | **-8.96311** | **0.78888** | **2.13083** | **-8.6184** | **0.74646** | **2.27751** | **-8.71333** |
| **H** | **-1.08262** | **1.58336** | **-8.41959** | **-1.09271** | **1.52485** | **-8.43472** | **-1.08651** | **1.49353** | **-8.42691** | **-1.28108** | **1.57387** | **-8.07462** | **-1.36977** | **1.79069** | **-8.26601** |
| **H** | **-0.04016** | **-0.33939** | **-6.2874** | **-0.0394** | **-0.36359** | **-6.2772** | **-0.03683** | **-0.36191** | **-6.23918** | **-0.32691** | **-0.22133** | **-5.79485** | **-0.57592** | **-0.15481** | **-6.05066** |
| **H** | **-2.33993** | **-1.296** | **-6.4297** | **-2.33428** | **-1.33169** | **-6.40504** | **-2.32649** | **-1.34235** | **-6.36731** | **-2.65779** | **-1.10348** | **-5.89267** | **-2.93852** | **-0.9233** | **-6.27046** |
| **H** | **-2.43855** | **0.40247** | **-5.9512** | **-2.44339** | **0.37339** | **-5.95504** | **-2.4459** | **0.36778** | **-5.93966** | **-2.70033** | **0.62795** | **-5.54028** | **-2.9156** | **0.78785** | **-5.82915** |
| **H** | **-2.80374** | **-0.07137** | **-7.6188** | **-2.80312** | **-0.12975** | **-7.61518** | **-2.79412** | **-0.15809** | **-7.5952** | **-3.06754** | **0.04561** | **-7.17283** | **-3.25229** | **0.30711** | **-7.50113** |
| **H** | **-0.49122** | **-2.11464** | **-7.96164** | **-0.48343** | **-2.16511** | **-7.92487** | **-0.46331** | **-2.18652** | **-7.86593** | **-0.82797** | **-2.09723** | **-7.34152** | **-1.10165** | **-1.92667** | **-7.70685** |
| **H** | **0.77209** | **-1.01051** | **-8.53017** | **0.77691** | **-1.06595** | **-8.50877** | **0.79501** | **-1.08899** | **-8.45719** | **0.47917** | **-1.08751** | **-7.97581** | **0.26706** | **-0.94487** | **-8.24844** |
| **H** | **-0.8712** | **-0.88991** | **-9.1835** | **-0.86618** | **-0.96022** | **-9.16533** | **-0.84497** | **-0.9995** | **-9.12384** | **-1.15279** | **-0.95253** | **-8.65258** | **-1.33457** | **-0.70535** | **-8.9672** |
| **H** | **2.10757** | **2.67716** | **-5.20532** | **2.02264** | **2.64183** | **-5.30065** | **2.006** | **2.66626** | **-5.28993** | **1.92941** | **2.77814** | **-4.91201** | **1.80864** | **2.71629** | **-4.94828** |
| **H** | **-1.03742** | **4.02656** | **-4.40187** | **-1.08991** | **4.03328** | **-4.48388** | **-1.11775** | **4.05307** | **-4.50913** | **-1.16276** | **4.31405** | **-4.24643** | **-1.24963** | **4.33911** | **-4.30388** |
| **H** | **5.09955** | **5.31096** | **-5.48917** | **5.03616** | **5.33124** | **-5.46783** | **5.00669** | **5.36895** | **-5.47631** | **5.03249** | **5.24601** | **-5.35752** | **4.95943** | **5.16986** | **-5.33651** |
| **H** | **4.81839** | **3.84001** | **-4.53638** | **4.83315** | **3.81161** | **-4.57593** | **4.80709** | **3.86008** | **-4.56551** | **4.66601** | **3.8707** | **-4.29661** | **4.62798** | **3.74824** | **-4.32746** |
| **H** | **2.45506** | **6.63218** | **-9.12754** | **2.45498** | **6.60312** | **-9.13663** | **2.44082** | **6.57898** | **-9.17522** | **2.50687** | **6.42006** | **-9.11354** | **2.4181** | **6.32393** | **-9.16843** |
| **H** | **1.04004** | **4.69474** | **-7.40988** | **1.02044** | **4.67366** | **-7.42939** | **1.00581** | **4.66518** | **-7.45069** | **0.97378** | **4.67468** | **-7.29205** | **0.91642** | **4.64161** | **-7.27246** |
| **H** | **-0.52206** | **8.73344** | **-5.4146** | **-0.49215** | **8.76094** | **-5.42999** | **-0.53884** | **8.76957** | **-5.51435** | **-0.4491** | **8.9026** | **-5.63977** | **-0.3187** | **8.96061** | **-5.56495** |
| **H** | **0.08567** | **9.67993** | **-6.78941** | **0.18852** | **9.69179** | **-6.77986** | **0.14436** | **9.68625** | **-6.87263** | **0.21192** | **9.71703** | **-7.07317** | **0.4128** | **9.74828** | **-6.97856** |
| **H** | **0.53069** | **6.76306** | **-6.3719** | **0.54764** | **6.76154** | **-6.37133** | **0.51656** | **6.76361** | **-6.42342** | **0.53561** | **6.82559** | **-6.42919** | **0.58223** | **6.83796** | **-6.35425** |
| **H** | **1.91328** | **7.25831** | **-4.81634** | **1.90256** | **7.20231** | **-4.78641** | **1.85954** | **7.23252** | **-4.83636** | **1.91406** | **7.38162** | **-4.89263** | **1.97535** | **7.31577** | **-4.80841** |
| **H** | **5.33935** | **7.60517** | **-3.44134** | **5.26075** | **7.49675** | **-3.22678** | **5.20762** | **7.56442** | **-3.2625** | **5.33844** | **7.68982** | **-3.50366** | **5.35408** | **7.47246** | **-3.29968** |
| **H** | **3.94443** | **9.37211** | **-4.3344** | **3.92225** | **9.28589** | **-4.14792** | **3.86528** | **9.33446** | **-4.21434** | **4.02963** | **9.4384** | **-4.54943** | **4.13841** | **9.29646** | **-4.33764** |
| **H** | **0.31863** | **3.79473** | **-2.31344** | **0.10564** | **3.85049** | **-2.32386** | **0.06689** | **3.90417** | **-2.34033** | **0.1828** | **4.18388** | **-2.14194** | **0.0343** | **4.05383** | **-2.17694** |
| **H** | **-0.84181** | **2.4592** | **-2.56782** | **-0.94845** | **2.45558** | **-2.63454** | **-0.97923** | **2.50057** | **-2.6385** | **-1.03191** | **2.88206** | **-2.29886** | **-1.22489** | **2.81001** | **-2.42648** |
| **H** | **1.38471** | **1.72231** | **-2.06191** | **1.85415** | **2.49173** | **-2.61215** | **1.82284** | **2.54963** | **-2.60019** | **1.15102** | **2.08366** | **-1.73162** | **0.91321** | **1.90113** | **-1.84809** |
| **H** | **0.15083** | **1.39347** | **-5.01989** | **0.08226** | **1.37473** | **-5.0035** | **0.07035** | **1.3938** | **-4.98774** | **-0.10037** | **1.58944** | **-4.65064** | **-0.27343** | **1.59927** | **-4.82162** |
| **H** | **0.45772** | **6.53069** | **-10.59221** | **0.44336** | **6.53315** | **-10.58668** | **0.43906** | **6.47953** | **-10.63672** | **0.55887** | **6.26959** | **-10.63642** | **0.14204** | **6.41995** | **-10.34894** |
| **H** | **-0.35753** | **5.41892** | **-9.47125** | **-0.37511** | **5.42657** | **-9.46215** | **-0.38137** | **5.3841** | **-9.50281** | **-0.33261** | **5.27482** | **-9.46486** | **-0.44685** | **5.20962** | **-9.1875** |
| **H** | **3.02112** | **3.91866** | **-11.34398** | **2.97271** | **3.90517** | **-11.37516** | **2.98603** | **3.85375** | **-11.37388** | **3.04899** | **3.5316** | **-11.11279** | **2.27531** | **4.12057** | **-12.11015** |
| **H** | **2.85378** | **5.6396** | **-11.20374** | **2.81087** | **5.62765** | **-11.24127** | **2.81489** | **5.57709** | **-11.26418** | **2.92761** | **5.26298** | **-11.12401** | **1.92688** | **5.80073** | **-11.83227** |
| **H** | **6.41855** | **0.31893** | **-9.52546** | **6.421** | **0.35138** | **-9.56053** | **6.43716** | **0.33764** | **-9.49447** | **5.14761** | **-0.95839** | **-9.1781** | **5.00518** | **-1.1118** | **-9.16119** |
| **H** | **4.61226** | **-1.25318** | **-11.19097** | **4.63241** | **-1.28538** | **-11.17859** | **4.6629** | **-1.32599** | **-11.10106** | **5.18759** | **0.51992** | **-11.66737** | **5.78766** | **0.64627** | **-11.30375** |
| **H** | **3.95952** | **-3.28596** | **-10.44222** | **3.5123** | **-3.24142** | **-8.68655** | **3.53874** | **-3.25523** | **-8.58741** | **4.92095** | **-2.35179** | **-13.4067** | **7.55145** | **-1.60241** | **-13.11344** |
| **H** | **3.4174** | **-3.21499** | **-8.74465** | **5.12657** | **-3.8294** | **-9.0826** | **5.1561** | **-3.84127** | **-8.9737** | **5.3566** | **-0.63772** | **-13.6273** | **6.4146** | **-0.24522** | **-13.31229** |
| **H** | **5.02107** | **-3.84181** | **-9.12458** | **4.03129** | **-3.31984** | **-10.39119** | **4.06139** | **-3.3511** | **-10.29009** | **6.61823** | **-1.89323** | **-13.54426** | **5.85546** | **-1.93107** | **-13.46782** |
| **H** | **6.96163** | **-0.07608** | **-11.91805** | **6.9342** | **-0.06525** | **-11.95594** | **6.96531** | **-0.10834** | **-11.88134** | **7.57904** | **-0.5501** | **-9.39632** | **7.439** | **-1.00554** | **-8.70239** |
| **H** | **6.74802** | **1.66344** | **-11.62376** | **6.68895** | **1.67318** | **-11.68156** | **6.71272** | **1.63267** | **-11.63092** | **6.92298** | **0.4082** | **-8.05481** | **6.54105** | **-0.15581** | **-7.43064** |
| **H** | **5.46545** | **0.76077** | **-12.42007** | **5.4131** | **0.7333** | **-12.44512** | **5.44436** | **0.67862** | **-12.3894** | **7.29671** | **1.19585** | **-9.60543** | **7.38461** | **0.77461** | **-8.69013** |

# Table S11. Lowest-energy Conformers Optimized at the M062X/6-311+G (d, p) Level of **1d** with Relative Energies < 3.0 kcal/mol

| **1d_C1** | G 2697.1339 Ha  Boltzman pop. 28.92% | **1d_C2** | G 2697.1331 Ha  Boltzman pop. 12.87% | **1d_C3** | G 2697.1333 Ha  Boltzman pop. 11.77% |
| --- | --- | --- | --- | --- | --- |
| 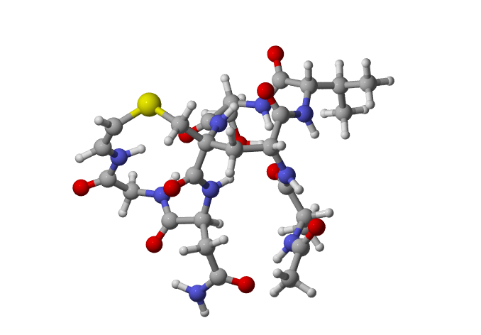 | | 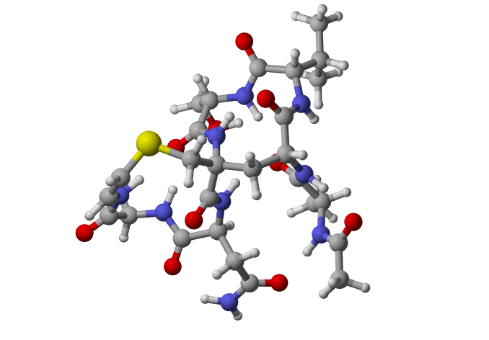 | | 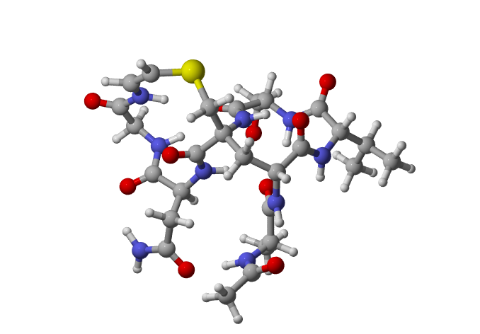 | |
| **1d_C4** | G 2697.1293 Ha  Boltzman pop. 1.72% | **1d_C5** | G 2697.1264 Ha  Boltzman pop. 4.52% | **1d_C6** | G 2697.1325 Ha  Boltzman pop. 5.35% |
| 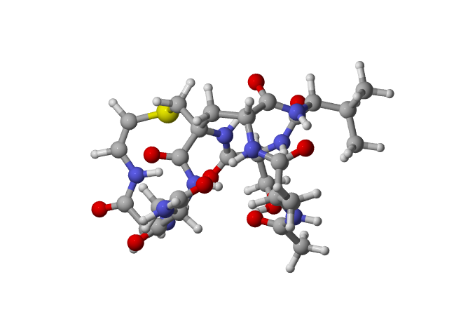 | | 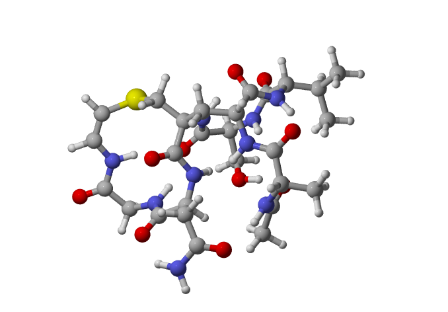 | | 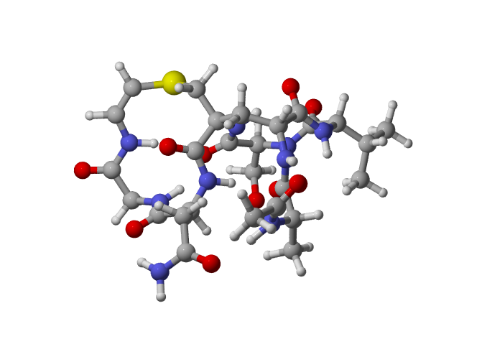 | |
| **1d_C7** | G 2697.1311 Ha  Boltzman pop. 4.02% | **1d_C8** | G 2697.1345 Ha  Boltzman pop. 30.83% |  | |
| 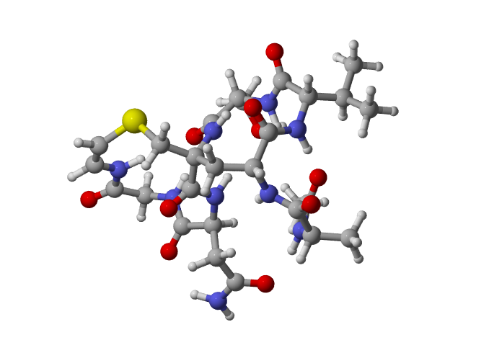 | | 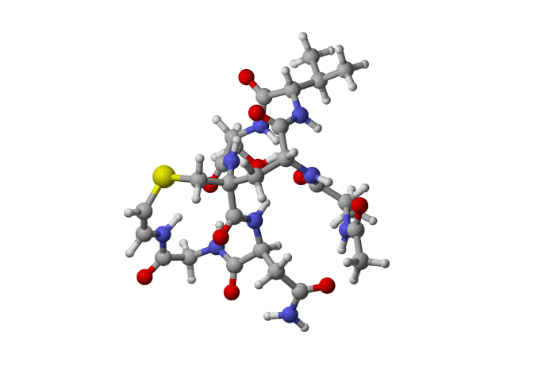 | |  |  |

# Table S12. Atomic Coordinates for the Lowest-energy Conformers of **1d** (**1d_C1**–**1d_C8**)

| **Atoms** | | **1d_C1** | | | | | **1d_C2** | | | | | **1d_C3** | | | | **1d_C4** | | | **1d_C5** | | |
| --- | --- | --- | --- | --- | --- | --- | --- | --- | --- | --- | --- | --- | --- | --- | --- | --- | --- | --- | --- | --- | --- |
|  |  | **x** | | **y** | **z** | | **x** | **y** | | **z** | | **x** | | **y** | **z** | **x** | **y** | **z** | **x** | **y** | **z** |
| **N** | | **-6.20301** | | **-9.97171** | **21.48197** | | **-6.23226** | **-9.92504** | | **21.38868** | | **-6.23594** | | **-9.90362** | **21.45054** | **-5.75294** | **-10.12601** | **20.99733** | **-5.69859** | **-9.91832** | **21.28002** |
| **C** | | **-5.44044** | | **-8.84332** | **22.02548** | | **-5.47046** | **-8.80104** | | **21.94236** | | **-5.4509** | | **-8.80106** | **22.01169** | **-5.42123** | **-8.82415** | **21.6032** | **-5.26404** | **-8.6602** | **21.8947** |
| **C** | | **-4.20463** | | **-8.3233** | **21.20576** | | **-4.21364** | **-8.2964** | | **21.14565** | | **-4.20038** | | **-8.2964** | **21.20302** | **-4.15828** | **-8.23098** | **20.91494** | **-3.99964** | **-8.063** | **21.2235** |
| **C** | | **-5.16574** | | **-8.86696** | **23.54341** | | **-5.22606** | **-8.82281** | | **23.46559** | | **-5.18458** | | **-8.84638** | **23.53078** | **-5.24364** | **-8.7638** | **23.1478** | **-5.09221** | **-8.68396** | **23.43931** |
| **N** | | **-5.74281** | | **-9.81348** | **24.32167** | | **-5.83065** | **-9.75975** | | **24.23292** | | **-5.77006** | | **-9.7965** | **24.29674** | **-5.8819** | **-9.64382** | **23.94726** | **-5.77724** | **-9.59025** | **24.17224** |
| **O** | | **-4.40911** | | **-8.01034** | **24.01364** | | **-4.46903** | **-7.97493** | | **23.95088** | | **-4.42921** | | **-7.99464** | **24.01297** | **-4.45622** | **-7.92279** | **23.60539** | **-4.26399** | **-7.92326** | **23.95899** |
| **C** | | **-5.2612** | | **-10.02334** | **25.69252** | | **-5.39385** | **-9.97459** | | **25.61937** | | **-5.30465** | | **-10.00979** | **25.67353** | **-5.48661** | **-9.75108** | **25.35652** | **-5.35568** | **-9.8692** | **25.5504** |
| **C** | | **-6.20768** | | **-10.9165** | **26.52845** | | **-6.37334** | **-10.91963** | | **26.36385** | | **-6.23163** | | **-10.95375** | **26.47511** | **-6.50003** | **-10.5573** | **26.20311** | **-6.39699** | **-10.69659** | **26.34045** |
| **C** | | **-3.78603** | | **-10.51333** | **25.7262** | | **-3.90464** | **-10.41634** | | **25.68718** | | **-3.81324** | | **-10.44315** | **25.72101** | **-4.03143** | **-10.2856** | **25.47709** | **-3.93822** | **-10.51188** | **25.56971** |
| **C** | | **-7.56701** | | **-10.23367** | **26.74668** | | **-6.31162** | **-12.3779** | | **25.87771** | | **-7.61312** | | **-10.3179** | **26.69743** | **-7.84529** | **-9.81898** | **26.29947** | **-7.70828** | **-9.91164** | **26.50894** |
| **C** | | **-6.37495** | | **-12.33844** | **25.96746** | | **-6.16892** | **-10.83994** | | **27.88454** | | **-6.35113** | | **-12.36487** | **25.87493** | **-6.69162** | **-12.01013** | **25.73531** | **-6.64677** | **-12.09896** | **25.76129** |
| **N** | | **-2.49755** | | **-9.59968** | **22.54472** | | **-2.54767** | **-9.57697** | | **22.52806** | | **-2.5102** | | **-9.59303** | **22.54817** | **-2.69314** | **-9.7359** | **22.30517** | **-2.46599** | **-9.56818** | **22.52205** |
| **C** | | **-1.64533** | | **-10.61048** | **22.85838** | | **-1.71239** | **-10.59161** | | **22.87126** | | **-1.66648** | | **-10.60527** | **22.87117** | **-1.96718** | **-10.83058** | **22.63578** | **-1.73783** | **-10.68496** | **22.7746** |
| **O** | | **-0.77193** | | **-11.0657** | **22.11724** | | **-0.83543** | **-11.07204** | | **22.15018** | | **-0.79815** | | **-11.08191** | **22.13555** | **-1.19976** | **-11.44764** | **21.88714** | **-0.84931** | **-11.13167** | **22.04194** |
| **C** | | **-1.90409** | | **-11.23637** | **24.25893** | | **-1.99443** | **-11.18122** | | **24.28245** | | **-1.92052** | | **-11.18934** | **24.29248** | **-2.23347** | **-11.34355** | **24.08332** | **-2.23083** | **-11.5152** | **24.00305** |
| **O** | | **-2.05203** | | **-9.33682** | **18.99261** | | **-2.0302** | **-9.35714** | | **18.98247** | | **-2.03562** | | **-9.33848** | **19.00255** | **-1.75788** | **-9.09215** | **18.89867** | **-2.04508** | **-9.09799** | **18.97133** |
| **C** | | **-1.80034** | | **-7.62481** | **21.18236** | | **-1.80146** | **-7.62632** | | **21.15808** | | **-1.789** | | **-7.62579** | **21.19091** | **-1.71137** | **-7.71396** | **21.27763** | **-1.54824** | **-7.57494** | **21.30028** |
| **C** | | **-2.505** | | **-9.80268** | **20.03386** | | **-2.50945** | **-9.80727** | | **20.01894** | | **-2.50994** | | **-9.79557** | **20.03859** | **-2.39996** | **-9.66103** | **19.78026** | **-2.43513** | **-9.61419** | **20.01033** |
| **C** | | **-2.71155** | | **-8.8901** | **21.27956** | | **-2.72952** | **-8.87982** | | **21.25119** | | **-2.71426** | | **-8.88155** | **21.28272** | **-2.71301** | **-8.89633** | **21.09836** | **-2.56217** | **-8.75889** | **21.30309** |
| **O** | | **-1.12854** | | **-12.55372** | **17.38415** | | **-1.11079** | **-12.60137** | | **17.42589** | | **-1.16187** | | **-12.58625** | **17.40391** | **-0.97139** | **-12.31762** | **16.92385** | **-1.18984** | **-12.12894** | **17.12292** |
| **C** | | **-1.39176** | | **-12.31934** | **18.5679** | | **-1.39615** | **-12.35173** | | **18.60136** | | **-1.43115** | | **-12.33156** | **18.58202** | **-1.36064** | **-12.1111** | **18.07029** | **-1.42301** | **-12.00587** | **18.32745** |
| **C** | | **-2.85108** | | **-12.03162** | **19.01538** | | **-2.86091** | **-12.04154** | | **19.01481** | | **-2.88824** | | **-12.01452** | **19.01482** | **-2.81687** | **-11.62856** | **18.33854** | **-2.86723** | **-11.74303** | **18.83242** |
| **N** | | **-2.93184** | | **-11.08958** | **20.1287** | | **-2.95309** | **-11.08808** | | **20.11757** | | **-2.9573** | | **-11.0753** | **20.13066** | **-2.95828** | **-10.88415** | **19.59089** | **-2.9364** | **-10.89644** | **20.02957** |
| **O** | | **2.48437** | | **-10.96192** | **18.15201** | | **2.50401** | **-11.04795** | | **18.25178** | | **2.45893** | | **-11.02091** | **18.17973** | **2.64422** | **-11.12342** | **18.36757** | **2.53243** | **-10.80844** | **17.94755** |
| **C** | | **1.60427** | | **-11.08319** | **18.9918** | | **1.60518** | **-11.14915** | | **19.07421** | | **1.5772** | | **-11.12657** | **19.02018** | **1.64821** | **-11.24867** | **19.06486** | **1.65274** | **-10.95695** | **18.78268** |
| **C** | | **0.94959** | | **-12.4459** | **19.28008** | | **0.92779** | **-12.50038** | | **19.36386** | | **0.90328** | | **-12.47932** | **19.31227** | **0.82852** | **-12.55169** | **19.08419** | **0.92752** | **-12.30189** | **18.95916** |
| **N** | | **-0.4766** | | **-12.36337** | **19.55923** | | **-0.50274** | **-12.39711** | | **19.61229** | | **-0.52391** | | **-12.37875** | **19.58179** | **-0.60718** | **-12.33657** | **19.17781** | **-0.48524** | **-12.1738** | **19.28982** |
| **N** | | **1.12561** | | **-10.03513** | **19.7476** | | **1.12367** | **-10.08695** | | **19.80813** | | **1.11382** | | **-10.07049** | **19.77313** | **1.1721** | **-10.24956** | **19.88904** | **1.2376** | **-9.96002** | **19.63661** |
| **S** | | **-0.02101** | | **-7.81542** | **21.64868** | | **-0.03407** | **-7.83341** | | **21.66158** | | **-0.01324** | | **-7.83488** | **21.66349** | **-0.02628** | **-8.1525** | **21.90724** | **0.20292** | **-7.947** | **21.76468** |
| **C** | | **0.89816** | | **-7.65835** | **20.13275** | | **0.91711** | **-7.70361** | | **20.16292** | | **0.91283** | | **-7.69076** | **20.15034** | **1.09427** | **-7.92073** | **20.54371** | **1.14936** | **-7.64029** | **20.28785** |
| **C** | | **1.39841** | | **-8.71085** | **19.45816** | | **1.41833** | **-8.76928** | | **19.51007** | | **1.40319** | | **-8.7502** | **19.47969** | **1.59121** | **-8.9346** | **19.80944** | **1.59538** | **-8.63129** | **19.49324** |
| **C** | | **-1.42077** | | **-12.69329** | **24.29786** | | **-1.49897** | **-12.63134** | | **24.37636** | | **-1.39556** | | **-12.6191** | **24.40239** | **-1.86578** | **-12.82873** | **24.20309** | **-2.23747** | **-12.98731** | **23.5547** |
| **O** | | **-2.16944** | | **-13.53745** | **23.42791** | | **-2.23633** | **-13.5136** | | **23.53515** | | **-2.20284** | | **-13.45206** | **23.56861** | **-2.72484** | **-13.65925** | **23.42961** | **-3.06324** | **-13.13731** | **22.39476** |
| **N** | | **-3.323** | | **-11.1233** | **24.59315** | | **-3.42054** | **-11.07259** | | **24.58777** | | **-3.33644** | | **-11.08353** | **24.61547** | **-3.61793** | **-11.08538** | **24.44664** | **-3.57078** | **-11.12171** | **24.40103** |
| **C** | | **-3.73067** | | **-11.61362** | **17.8059** | | **-3.709** | **-11.62409** | | **17.78278** | | **-3.74814** | | **-11.57475** | **17.79822** | **-3.31727** | **-10.87634** | **17.09299** | **-3.77239** | **-11.22126** | **17.68789** |
| **C** | | **-4.3739** | | **-12.80625** | **17.10759** | | **-4.35136** | **-12.81492** | | **17.08047** | | **-4.41065** | | **-12.74905** | **17.08632** | **-4.80611** | **-10.6085** | **17.0538** | **-4.44305** | **-12.35788** | **16.92273** |
| **O** | | **-5.56644** | | **-13.10691** | **17.30089** | | **-5.55154** | **-13.09933** | | **17.24965** | | **-5.61363** | | **-13.01831** | **17.25739** | **-5.51077** | **-10.44281** | **18.05855** | **-5.58682** | **-12.74829** | **17.21247** |
| **N** | | **-3.57516** | | **-13.50926** | **16.28503** | | **-3.54365** | **-13.53466** | | **16.28151** | | **-3.61701** | | **-13.47098** | **16.27462** | **-5.34527** | **-10.52147** | **15.80954** | **-3.7146** | **-12.90875** | **15.9323** |
| **O** | | **-3.09993** | | **-10.31658** | **26.72173** | | **-3.22433** | **-10.15613** | | **26.67234** | | **-3.1264** | | **-10.17139** | **26.69995** | **-3.31716** | **-9.97071** | **26.42013** | **-3.22192** | **-10.44005** | **26.55914** |
| **C** | | **-5.91845** | | **-11.29116** | **21.50872** | | **-5.96454** | **-11.24784** | | **21.43228** | | **-5.97015** | | **-11.2282** | **21.46** | **-6.82863** | **-10.90835** | **21.24325** | **-6.99046** | **-10.33552** | **21.35343** |
| **C** | | **-6.95286** | | **-12.1748** | **20.75965** | | **-6.99529** | **-12.12447** | | **20.66964** | | **-7.0215** | | **-12.08853** | **20.70798** | **-6.98042** | **-12.12441** | **20.30407** | **-7.46237** | **-11.43807** | **20.38585** |
| **N** | | **-7.0948** | | **-11.79568** | **19.35125** | | **-7.10602** | **-11.75434** | | **19.25607** | | **-7.15428** | | **-11.69905** | **19.30132** | **-6.66629** | **-13.34872** | **21.04346** | **-6.43679** | **-12.40756** | **19.99091** |
| **C** | | **-7.88576** | | **-10.77067** | **18.95159** | | **-7.87676** | **-10.72315** | | **18.83358** | | **-7.92563** | | **-10.65777** | **18.90535** | **-5.40162** | **-13.80672** | **21.24507** | **-5.83869** | **-13.26462** | **20.85673** |
| **C** | | **-8.11286** | | **-10.62834** | **17.46357** | | **-8.07306** | **-10.58943** | | **17.34037** | | **-8.13796** | | **-10.49891** | **17.41642** | **-5.27098** | **-15.10956** | **22.0031** | **-5.15683** | **-14.47171** | **20.24226** |
| **O** | | **-4.95057** | | **-11.78247** | **22.11542** | | **-5.01615** | **-11.74641** | | **22.06309** | | **-5.00443** | | **-11.73685** | **22.05192** | **-7.62918** | **-10.74272** | **22.17613** | **-7.79684** | **-9.79005** | **22.11281** |
| **C** | | **-6.62414** | | **-13.66108** | **20.87822** | | **-6.68691** | **-13.61369** | | **20.8049** | | **-6.71793** | | **-13.58054** | **20.81957** | **-8.40266** | **-12.19585** | **19.7479** | **-8.73687** | **-12.12182** | **20.89871** |
| **O** | | **-8.40514** | | **-9.98456** | **19.76684** | | **-8.40221** | **-9.92455** | | **19.63264** | | **-8.43971** | | **-9.87106** | **19.72281** | **-4.40658** | **-13.19982** | **20.82055** | **-5.85294** | **-13.08478** | **22.0877** |
| **H** | | **-7.06365** | | **-9.73641** | **20.9674** | | **-7.0797** | **-9.68384** | | **20.85535** | | **-7.09381** | | **-9.64606** | **20.94421** | **-5.3235** | **-10.28317** | **20.08482** | **-5.05203** | **-10.40939** | **20.67284** |
| **H** | | **-6.14325** | | **-8.00789** | **21.9189** | | **-6.16322** | **-7.95963** | | **21.8184** | | **-6.13387** | | **-7.9485** | **21.91349** | **-6.23733** | **-8.12025** | **21.37736** | **-6.0637** | **-7.92868** | **21.71169** |
| **H** | | **-4.13619** | | **-7.27277** | **21.4939** | | **-4.13938** | **-7.24585** | | **21.43248** | | **-4.1187** | | **-7.24816** | **21.4957** | **-4.08815** | **-7.20473** | **21.27648** | **-3.89347** | **-7.07385** | **21.67041** |
| **H** | | **-4.50569** | | **-8.32767** | **20.15392** | | **-4.49372** | **-8.3004** | | **20.088** | | **-4.4941** | | **-8.29235** | **20.14904** | **-4.36488** | **-8.16957** | **19.84189** | **-4.22112** | **-7.90353** | **20.164** |
| **H** | | **-6.29246** | | **-10.53449** | **23.87833** | | **-6.39379** | **-10.46622** | | **23.78292** | | **-6.29846** | | **-10.52527** | **23.84051** | **-6.65227** | **-10.18433** | **23.54382** | **-6.59254** | **-10.03338** | **23.76017** |
| **H** | | **-5.2086** | | **-9.04201** | **26.17038** | | **-5.39952** | **-9.00348** | | **26.12** | | **-5.29636** | | **-9.03599** | **26.16911** | **-5.41743** | **-8.74302** | **25.77461** | **-5.21257** | **-8.91564** | **26.06656** |
| **H** | | **-5.70899** | | **-10.99893** | **27.50158** | | **-7.37506** | **-10.52572** | | **26.14002** | | **-5.74086** | | **-11.04661** | **27.45162** | **-6.05553** | **-10.58218** | **27.2059** | **-5.94603** | **-10.81843** | **27.33287** |
| **H** | | **-7.44612** | | **-9.2403** | **27.19114** | | **-6.43756** | **-12.47593** | | **24.79281** | | **-7.52728** | | **-9.33563** | **27.17371** | **-7.71258** | **-8.79654** | **26.66897** | **-7.52841** | **-8.9247** | **26.94856** |
| **H** | | **-8.18936** | | **-10.83051** | **27.42131** | | **-7.10567** | **-12.96437** | | **26.35046** | | **-8.22578** | | **-10.95361** | **27.34504** | **-8.51657** | **-10.34126** | **26.98925** | **-8.39149** | **-10.45493** | **27.16987** |
| **H** | | **-8.11797** | | **-10.11486** | **25.80655** | | **-5.35483** | **-12.84075** | | **26.14348** | | **-8.15581** | | **-10.18624** | **25.75415** | **-8.34818** | **-9.76478** | **25.32737** | **-8.22343** | **-9.765** | **25.55281** |
| **H** | | **-6.97411** | | **-12.94364** | **26.65497** | | **-6.90678** | **-11.46779** | | **28.39468** | | **-6.92589** | | **-13.00841** | **26.54857** | **-5.74401** | **-12.55671** | **25.69555** | **-7.35424** | **-12.64184** | **26.39641** |
| **H** | | **-5.41224** | | **-12.84206** | **25.83507** | | **-6.28689** | **-9.81436** | | **28.2485** | | **-5.37419** | | **-12.83217** | **25.71848** | **-7.34965** | **-12.54006** | **26.43184** | **-5.7266** | **-12.68956** | **25.7133** |
| **H** | | **-6.89882** | | **-12.34779** | **25.00267** | | **-5.1695** | **-11.17908** | | **28.17166** | | **-6.88214** | | **-12.36428** | **24.914** | **-7.16221** | **-12.06193** | **24.7461** | **-7.07542** | **-12.06806** | **24.75309** |
| **H** | | **-2.97331** | | **-9.17164** | **23.33087** | | **-3.03192** | **-9.13228** | | **23.29994** | | **-3.00078** | | **-9.1736** | **23.32986** | **-3.10938** | **-9.25553** | **23.09648** | **-2.96117** | **-9.171** | **23.314** |
| **H** | | **-1.34517** | | **-10.66748** | **25.01351** | | **-1.45668** | **-10.58408** | | **25.03068** | | **-1.37702** | | **-10.57715** | **25.02363** | **-1.60439** | **-10.76951** | **24.77644** | **-1.56104** | **-11.3727** | **24.85795** |
| **H** | | **-2.19015** | | **-6.89339** | **21.8943** | | **-2.19589** | **-6.88238** | | **21.85432** | | **-2.17301** | | **-6.8919** | **21.90334** | **-2.12959** | **-7.04954** | **22.03738** | **-1.89332** | **-6.86043** | **22.05075** |
| **H** | | **-1.85856** | | **-7.20988** | **20.17506** | | **-1.83479** | **-7.22185** | | **20.14543** | | **-1.83909** | | **-7.20796** | **20.18438** | **-1.60873** | **-7.17099** | **20.33776** | **-1.55622** | **-7.09485** | **20.32072** |
| **H** | | **-3.2312** | | **-12.9766** | **19.42156** | | **-3.26177** | **-12.97777** | | **19.42133** | | **-3.29309** | | **-12.95223** | **19.41406** | **-3.41805** | **-12.53301** | **18.48031** | **-3.24294** | **-12.71619** | **19.16495** |
| **H** | | **-3.3624** | | **-11.41478** | **20.98777** | | **-3.40489** | **-11.3997** | | **20.9709** | | **-3.42138** | | **-11.38668** | **20.97888** | **-3.43312** | **-11.37269** | **20.34243** | **-2.98323** | **-11.40455** | **20.9082** |
| **H** | | **1.11585** | | **-13.06471** | **18.39857** | | **1.10459** | **-13.13114** | | **18.49294** | | **1.0674** | | **-13.10522** | **18.43541** | **1.0571** | **-13.07863** | **18.15795** | **1.03352** | **-12.84032** | **18.0176** |
| **H** | | **1.4479** | | **-12.91092** | **20.13712** | | **1.40228** | **-12.96169** | | **20.23629** | | **1.39135** | | **-12.9445** | **20.17528** | **1.15418** | **-13.16763** | **19.92967** | **1.42308** | **-12.87467** | **19.75014** |
| **H** | | **-0.76264** | | **-12.0932** | **20.49749** | | **-0.80448** | **-12.11187** | | **20.54119** | | **-0.8111** | | **-12.07738** | **20.51087** | **-0.99232** | **-12.08707** | **20.08406** | **-0.73482** | **-11.99399** | **20.25903** |
| **H** | | **0.45635** | | **-10.21575** | **20.493** | | **0.43681** | **-10.25129** | | **20.54117** | | **0.44136** | | **-10.23936** | **20.51861** | **0.38918** | **-10.44371** | **20.5067** | **0.56389** | **-10.16927** | **20.36913** |
| **H** | | **1.14887** | | **-6.65484** | **19.80631** | | **1.1864** | **-6.70674** | | **19.83098** | | **1.17502** | | **-6.69078** | **19.82202** | **1.47176** | **-6.91723** | **20.38018** | **1.45883** | **-6.61944** | **20.09199** |
| **H** | | **2.04612** | | **-8.5729** | **18.59828** | | **2.08509** | **-8.6485** | | **18.66226** | | **2.05427** | | **-8.6225** | **18.62082** | **2.36042** | **-8.7639** | **19.06299** | **2.25786** | **-8.43773** | **18.65585** |
| **H** | | **-1.47087** | | **-13.04723** | **25.33505** | | **-1.55066** | **-12.94812** | | **25.42548** | | **-1.46723** | | **-12.92863** | **25.45264** | **-2.00021** | **-13.13352** | **25.24338** | **-2.59348** | **-13.61603** | **24.37738** |
| **H** | | **-0.38617** | | **-12.74138** | **23.95543** | | **-0.46227** | **-12.68248** | | **24.04069** | | **-0.34641** | | **-12.63841** | **24.08582** | **-0.81604** | **-12.96909** | **23.92329** | **-1.23007** | **-13.29509** | **23.27205** |
| **H** | | **-3.06284** | | **-13.63519** | **23.78332** | | **-3.12661** | **-13.61377** | | **23.89753** | | **-1.883** | | **-14.36037** | **23.62631** | **-2.55713** | **-13.50433** | **22.48704** | **-4.00282** | **-13.26815** | **22.63227** |
| **H** | | **-3.97** | | **-11.26138** | **23.82059** | | **-4.05279** | **-11.24011** | | **23.80893** | | **-3.98168** | | **-11.35091** | **23.87773** | **-4.3107** | **-11.3323** | **23.75434** | **-4.28067** | **-11.17232** | **23.68193** |
| **H** | | **-3.1305** | | **-11.02991** | **17.10584** | | **-3.08671** | **-11.05379** | | **17.09103** | | **-3.13021** | | **-10.99978** | **17.10631** | **-3.00161** | **-11.44842** | **16.2173** | **-3.18906** | **-10.58568** | **17.02067** |
| **H** | | **-4.54268** | | **-10.98676** | **18.17436** | | **-4.52114** | **-10.98424** | | **18.12787** | | **-4.55053** | | **-10.93294** | **18.16208** | **-2.80295** | **-9.91043** | **17.03674** | **-4.57502** | **-10.62474** | **18.1227** |
| **H** | | **-3.93996** | | **-14.34268** | **15.84618** | | **-3.90924** | **-14.36718** | | **15.84161** | | **-3.99789** | | **-14.29102** | **15.8245** | **-6.31377** | **-10.2506** | **15.71605** | **-4.08826** | **-13.70983** | **15.44299** |
| **H** | | **-2.57719** | | **-13.30327** | **16.27715** | | **-2.54319** | **-13.3413** | | **16.29426** | | **-2.61404** | | **-13.29097** | **16.28241** | **-4.78725** | **-10.63131** | **14.97683** | **-2.72492** | **-12.68298** | **15.86988** |
| **H** | | **-7.92194** | | **-11.97255** | **21.23517** | | **-7.97069** | **-11.90712** | | **21.12521** | | **-7.98727** | | **-11.87119** | **21.18318** | **-6.26543** | **-12.04575** | **19.48558** | **-7.70981** | **-10.89699** | **19.46309** |
| **H** | | **-6.6008** | | **-12.35086** | **18.63924** | | **-6.60445** | **-12.32033** | | **18.55797** | | **-6.6615** | | **-12.25364** | **18.58905** | **-7.43057** | **-13.82968** | **21.49698** | **-6.31939** | **-12.58181** | **18.98828** |
| **H** | | **-7.51259** | | **-11.32439** | **16.87447** | | **-7.83636** | **-9.56537** | | **17.03943** | | **-9.19933** | | **-10.66194** | **17.20313** | **-6.2316** | **-15.52526** | **22.31836** | **-5.13227** | **-14.43844** | **19.15133** |
| **H** | | **-9.17377** | | **-10.80767** | **17.26138** | | **-7.47045** | **-11.29755** | | **16.76827** | | **-7.90352** | | **-9.47011** | **17.13025** | **-4.76475** | **-15.8355** | **21.35962** | **-4.14699** | **-14.57603** | **20.64645** |
| **H** | | **-7.89522** | | **-9.59908** | **17.166** | | **-9.13207** | **-10.75676** | | **17.11882** | | **-7.54198** | | **-11.19755** | **16.8259** | **-4.63398** | **-14.94285** | **22.87625** | **-5.7238** | **-15.3575** | **20.54895** |
| **H** | | **-7.41627** | | **-14.24885** | **20.40735** | | **-7.47725** | **-14.19538** | | **20.32358** | | **-7.52508** | | **-14.15373** | **20.35606** | **-8.50961** | **-13.07194** | **19.10148** | **-9.1202** | **-12.79664** | **20.12834** |
| **H** | | **-6.54295** | | **-13.95273** | **21.92726** | | **-6.62847** | **-13.89886** | | **21.85724** | | **-6.62697** | | **-13.87518** | **21.86689** | **-8.61644** | **-11.30263** | **19.1548** | **-9.49701** | **-11.3749** | **21.13215** |
| **H** | | **-5.67663** | | **-13.90319** | **20.39011** | | **-5.73352** | **-13.8707** | | **20.33628** | | **-5.77993** | | **-13.83654** | **20.32021** | **-9.13654** | **-12.24745** | **20.55806** | **-8.52798** | **-12.69661** | **21.80398** |
| Atoms | **1d_C6** | | | | | | **1d_C7** | | | | **1d_C8** | | | | |  |  |  |  |  |  |
|  | x | | y | | | z | x | y | z | | x | | y | | z |  |  |  |  |  |  |
| N | **-6.26702** | | **-9.86334** | | | **21.36034** | **-5.76681** | **-9.69105** | **21.06439** | | **-6.20458** | | **-9.94643** | | **21.42293** |  |  |  |  |  |  |
| C | **-5.48353** | | **-8.76437** | | | **21.93098** | **-5.23965** | **-8.58199** | **21.83871** | | **-5.4429** | | **-8.82975** | | **21.99268** |  |  |  |  |  |  |
| C | **-4.21319** | | **-8.27488** | | | **21.14455** | **-3.92299** | **-7.97053** | **21.29813** | | **-4.19389** | | **-8.30599** | | **21.19827** |  |  |  |  |  |  |
| C | **-5.24615** | | **-8.80501** | | | **23.45495** | **-5.13835** | **-8.83943** | **23.36209** | | **-5.19094** | | **-8.8766** | | **23.51481** |  |  |  |  |  |  |
| N | **-5.85477** | | **-9.74632** | | | **24.21232** | **-5.75142** | **-9.91772** | **23.89501** | | **-5.79096** | | **-9.83052** | | **24.26338** |  |  |  |  |  |  |
| O | **-4.49234** | | **-7.95884** | | | **23.94925** | **-4.46504** | **-8.06015** | **24.05194** | | **-4.43828** | | **-8.0336** | | **24.01412** |  |  |  |  |  |  |
| C | **-5.4294** | | **-9.96198** | | | **25.60299** | **-5.48532** | **-10.28153** | **25.29399** | | **-5.34758** | | **-10.09595** | | **25.63981** |  |  |  |  |  |  |
| C | **-6.38971** | | **-10.94188** | | | **26.3259** | **-6.44827** | **-11.40287** | **25.75799** | | **-6.2595** | | **-11.16746** | | **26.28943** |  |  |  |  |  |  |
| C | **-3.92804** | | **-10.35686** | | | **25.68164** | **-3.98305** | **-10.62837** | **25.4664** | | **-3.83619** | | **-10.45476** | | **25.67783** |  |  |  |  |  |  |
| C | **-6.31048** | | **-12.38594** | | | **25.80129** | **-6.08802** | **-11.9077** | **27.16383** | | **-5.7537** | | **-11.58573** | | **27.67865** |  |  |  |  |  |  |
| C | **-6.17408** | | **-10.89842** | | | **27.84664** | **-7.90433** | **-10.90854** | **25.71857** | | **-7.71143** | | **-10.66521** | | **26.37859** |  |  |  |  |  |  |
| N | **-2.56259** | | **-9.57359** | | | **22.53394** | **-2.44219** | **-9.56517** | **22.53778** | | **-2.52014** | | **-9.61192** | | **22.54288** |  |  |  |  |  |  |
| C | **-1.73251** | | **-10.58753** | | | **22.88402** | **-1.70529** | **-10.68335** | **22.77099** | | **-1.68114** | | **-10.63133** | | **22.86146** |  |  |  |  |  |  |
| O | **-0.85648** | | **-11.08124** | | | **22.16883** | **-0.90906** | **-11.1751** | **21.96299** | | **-0.81626** | | **-11.10394** | | **22.12066** |  |  |  |  |  |  |
| C | **-2.01219** | | **-11.14754** | | | **24.31004** | **-2.01983** | **-11.40406** | **24.11534** | | **-1.93866** | | **-11.23331** | | **24.27116** |  |  |  |  |  |  |
| O | **-2.0148** | | **-9.3585** | | | **18.9964** | **-2.13899** | **-9.05672** | **18.95615** | | **-2.03727** | | **-9.33604** | | **18.99516** |  |  |  |  |  |  |
| C | **-1.79633** | | **-7.62666** | | | **21.17224** | **-1.46308** | **-7.61077** | **21.2949** | | **-1.78078** | | **-7.64237** | | **21.19608** |  |  |  |  |  |  |
| C | **-2.51466** | | **-9.80085** | | | **20.02686** | **-2.48226** | **-9.58249** | **20.00527** | | **-2.50617** | | **-9.80264** | | **20.0291** |  |  |  |  |  |  |
| C | **-2.73495** | | **-8.873** | | | **21.25783** | **-2.53532** | **-8.74512** | **21.31682** | | **-2.71162** | | **-8.89475** | | **21.27845** |  |  |  |  |  |  |
| O | **-1.1412** | | **-12.63005** | | | **17.44681** | **-1.3499** | **-12.07741** | **17.06199** | | **-1.13319** | | **-12.55219** | | **17.37753** |  |  |  |  |  |  |
| C | **-1.43217** | | **-12.36122** | | | **18.61662** | **-1.53536** | **-11.98527** | **18.2763** | | **-1.40813** | | **-12.32385** | | **18.55977** |  |  |  |  |  |  |
| C | **-2.89454** | | **-12.02534** | | | **19.01618** | **-2.95854** | **-11.72646** | **18.84366** | | **-2.86898** | | **-12.02007** | | **18.99191** |  |  |  |  |  |  |
| N | **-2.97653** | | **-11.07517** | | | **20.12188** | **-2.98827** | **-10.86427** | **20.03292** | | **-2.95071** | | **-11.08438** | | **20.11075** |  |  |  |  |  |  |
| O | **2.47739** | | **-11.09297** | | | **18.27981** | **2.46197** | **-10.87228** | **17.79724** | | **2.49129** | | **-11.02402** | | **18.19533** |  |  |  |  |  |  |
| C | **1.57794** | | **-11.18142** | | | **19.10333** | **1.58837** | **-11.01219** | **18.63972** | | **1.59964** | | **-11.13664** | | **19.02404** |  |  |  |  |  |  |
| C | **0.8852** | | **-12.52456** | | | **19.39579** | **0.83194** | **-12.34094** | **18.80414** | | **0.92208** | | **-12.49097** | | **19.29831** |  |  |  |  |  |  |
| N | **-0.5461** | | **-12.40757** | | | **19.63522** | **-0.5624** | **-12.18861** | **19.19707** | | **-0.50589** | | **-12.38899** | | **19.56173** |  |  |  |  |  |  |
| N | **1.10978** | | **-10.11319** | | | **19.83589** | **1.21156** | **-10.02174** | **19.51833** | | **1.1267** | | **-10.08546** | | **19.77905** |  |  |  |  |  |  |
| S | **-0.03193** | | **-7.84694** | | | **21.68102** | **0.27916** | **-8.05817** | **21.7283** | | **-0.00927** | | **-7.86102** | | **21.67979** |  |  |  |  |  |  |
| C | **0.9246** | | **-7.7278** | | | **20.18473** | **1.21862** | **-7.72746** | **20.25166** | | **0.92816** | | **-7.70802** | | **20.17466** |  |  |  |  |  |  |
| C | **1.41762** | | **-8.79882** | | | **19.53474** | **1.61744** | **-8.70334** | **19.41436** | | **1.42127** | | **-8.76366** | | **19.49975** |  |  |  |  |  |  |
| C | **-1.48785** | | **-12.57447** | | | **24.45332** | **-1.56223** | **-12.86784** | **24.05052** | | **-1.41568** | | **-12.67382** | | **24.35647** |  |  |  |  |  |  |
| O | **-2.28301** | | **-13.42263** | | | **23.62321** | **-2.40124** | **-13.63026** | **23.17379** | | **-2.15511** | | **-13.57138** | | **23.53324** |  |  |  |  |  |  |
| N | **-3.43339** | | **-11.04116** | | | **24.60933** | **-3.44285** | **-11.32353** | **24.40933** | | **-3.3626** | | **-11.15384** | | **24.59807** |  |  |  |  |  |  |
| C | **-3.72427** | | **-11.58768** | | | **17.77801** | **-3.91427** | **-11.23515** | **17.73041** | | **-3.72843** | | **-11.58305** | | **17.7747** |  |  |  |  |  |  |
| C | **-4.38377** | | **-12.76105** | | | **17.06167** | **-4.57943** | **-12.40612** | **17.0126** | | **-4.37496** | | **-12.76279** | | **17.05736** |  |  |  |  |  |  |
| O | **-5.59326** | | **-13.01555** | | | **17.20778** | **-5.65866** | **-12.87752** | **17.40796** | | **-5.57266** | | **-13.05336** | | **17.23335** |  |  |  |  |  |  |
| N | **-3.58035** | | **-13.4989** | | | **16.27431** | **-3.92069** | **-12.89426** | **15.94327** | | **-3.57386** | | **-13.46575** | | **16.23709** |  |  |  |  |  |  |
| O | **-3.24695** | | **-10.0273** | | | **26.64692** | **-3.33372** | **-10.29583** | **26.44663** | | **-3.13527** | | **-10.11567** | | **26.62223** |  |  |  |  |  |  |
| C | **-6.01759** | | **-11.19117** | | | **21.38453** | **-7.11311** | **-9.88141** | **20.92805** | | **-5.9412** | | **-11.27061** | | **21.44934** |  |  |  |  |  |  |
| C | **-7.06496** | | **-12.04294** | | | **20.61696** | **-7.56339** | **-11.12235** | **20.13124** | | **-6.98041** | | **-12.13475** | | **20.6837** |  |  |  |  |  |  |
| N | **-7.16329** | | **-11.66202** | | | **19.20517** | **-6.57314** | **-12.20472** | **20.08066** | | **-7.10698** | | **-11.74387** | | **19.27717** |  |  |  |  |  |  |
| C | **-7.91296** | | **-10.6141** | | | **18.78573** | **-6.09153** | **-12.85735** | **21.16601** | | **-7.88178** | | **-10.70638** | | **18.87824** |  |  |  |  |  |  |
| C | **-8.09117** | | **-10.46302** | | | **17.2915** | **-5.30352** | **-14.1242** | **20.88826** | | **-8.09541** | | **-10.55155** | | **17.38944** |  |  |  |  |  |  |
| O | **-5.07098** | | **-11.70822** | | | **21.99951** | **-7.93784** | **-9.08823** | **21.37149** | | **-4.99132** | | **-11.78076** | | **22.06888** |  |  |  |  |  |  |
| C | **-6.78403** | | **-13.53805** | | | **20.74497** | **-8.93922** | **-11.61316** | **20.60257** | | **-6.67185** | | **-13.62605** | | **20.79379** |  |  |  |  |  |  |
| O | **-8.43451** | | **-9.81534** | | | **19.58658** | **-6.25822** | **-12.45363** | **22.33083** | | **-8.39739** | | **-9.91894** | | **19.69458** |  |  |  |  |  |  |
| H | **-7.1112** | | **-9.59979** | | | **20.83444** | **-5.13069** | **-10.38302** | **20.69158** | | **-7.05681** | | **-9.69718** | | **20.90103** |  |  |  |  |  |  |
| H | **-6.15726** | | **-7.90667** | | | **21.81518** | **-5.9838** | **-7.78046** | **21.7529** | | **-6.1398** | | **-7.98902** | | **21.8882** |  |  |  |  |  |  |
| H | **-4.12805** | | **-7.22577** | | | **21.43323** | **-3.78139** | **-7.06679** | **21.89292** | | **-4.11621** | | **-7.26313** | | **21.51131** |  |  |  |  |  |  |
| H | **-4.48561** | | **-8.27407** | | | **20.08486** | **-4.09171** | **-7.6505** | **20.2662** | | **-4.48192** | | **-8.28309** | | **20.14302** |  |  |  |  |  |  |
| H | **-6.39397** | | **-10.46415** | | | **23.75125** | **-6.19038** | **-10.60559** | **23.28523** | | **-6.32971** | | **-10.54184** | | **23.79113** |  |  |  |  |  |  |
| H | **-5.46904** | | **-8.99651** | | | **26.11285** | **-5.62948** | **-9.39389** | **25.91624** | | **-5.41548** | | **-9.1665** | | **26.21209** |  |  |  |  |  |  |
| H | **-7.39887** | | **-10.5567** | | | **26.12033** | **-6.35043** | **-12.23972** | **25.05246** | | **-6.237** | | **-12.05687** | | **25.63962** |  |  |  |  |  |  |
| H | **-6.46094** | | **-12.46086** | | | **24.71786** | **-5.0827** | **-12.33567** | **27.2102** | | **-4.75139** | | **-12.01892** | | **27.64513** |  |  |  |  |  |  |
| H | **-7.08285** | | **-12.99822** | | | **26.27764** | **-6.7989** | **-12.68107** | **27.47231** | | **-6.43265** | | **-12.3287** | | **28.10917** |  |  |  |  |  |  |
| H | **-5.33894** | | **-12.83817** | | | **26.02669** | **-6.13273** | **-11.09587** | **27.89837** | | **-5.71638** | | **-10.72672** | | **28.35714** |  |  |  |  |  |  |
| H | **-6.90182** | | **-11.54615** | | | **28.34636** | **-8.58441** | **-11.71795** | **26.00373** | | **-8.35609** | | **-11.44429** | | **26.79787** |  |  |  |  |  |  |
| H | **-6.29747** | | **-9.8837** | | | **28.23825** | **-8.19927** | **-10.55689** | **24.72605** | | **-8.1231** | | **-10.37968** | | **25.40555** |  |  |  |  |  |  |
| H | **-5.16966** | | **-11.23634** | | | **28.11801** | **-8.051** | **-10.08275** | **26.42511** | | **-7.77459** | | **-9.78971** | | **27.03529** |  |  |  |  |  |  |
| H | **-3.06296** | | **-9.14035** | | | **23.30193** | **-2.92813** | **-9.16657** | **23.3381** | | **-2.99448** | | **-9.17666** | | **23.32632** |  |  |  |  |  |  |
| H | **-1.4834** | | **-10.52115** | | | **25.03989** | **-1.49244** | **-10.8922** | **24.9305** | | **-1.40473** | | **-10.62751** | | **25.01514** |  |  |  |  |  |  |
| H | **-2.18707** | | **-6.8826** | | | **21.87032** | **-1.76132** | **-6.87385** | **22.0434** | | **-2.16673** | | **-6.9081** | | **21.90716** |  |  |  |  |  |  |
| H | **-1.8232** | | **-7.21788** | | | **20.16111** | **-1.46546** | **-7.13709** | **20.31242** | | **-1.82248** | | **-7.22236** | | **20.19011** |  |  |  |  |  |  |
| H | **-3.31756** | | **-12.95505** | | | **19.41527** | **-3.31454** | **-12.69821** | **19.20368** | | **-3.26635** | | **-12.96266** | | **19.38704** |  |  |  |  |  |  |
| H | **-3.46106** | | **-11.3738** | | | **20.96331** | **-2.95721** | **-11.35181** | **20.91631** | | **-3.39197** | | **-11.41028** | | **20.96428** |  |  |  |  |  |  |
| H | **1.06105** | | **-13.16135** | | | **18.52912** | **0.88377** | **-12.85208** | **17.843** | | **1.08952** | | **-13.10809** | | **18.41584** |  |  |  |  |  |  |
| H | **1.3511** | | **-12.98502** | | | **20.27346** | **1.34487** | **-12.95195** | **19.5544** | | **1.40363** | | **-12.96708** | | **20.15883** |  |  |  |  |  |  |
| H | **-0.84885** | | **-12.0934** | | | **20.5551** | **-0.77088** | **-12.03331** | **20.17955** | | **-0.79837** | | **-12.11859** | | **20.498** |  |  |  |  |  |  |
| H | **0.42085** | | **-10.26801** | | | **20.56931** | **0.53746** | **-10.22664** | **20.25077** | | **0.44602** | | **-10.26035** | | **20.51538** |  |  |  |  |  |  |
| H | **1.2027** | | **-6.73387** | | | **19.85115** | **1.56157** | **-6.71126** | **20.09136** | | **1.19612** | | **-6.70625** | | **19.85672** |  |  |  |  |  |  |
| H | **2.0865** | | **-8.68625** | | | **18.68751** | **2.27631** | **-8.50464** | **18.57534** | | **2.08047** | | **-8.63029** | | **18.64791** |  |  |  |  |  |  |
| H | **-1.57412** | | **-12.86566** | | | **25.50779** | **-1.57456** | **-13.28809** | **25.06186** | | **-1.43881** | | **-12.99** | | **25.40679** |  |  |  |  |  |  |
| H | **-0.43437** | | **-12.59888** | | | **24.152** | **-0.54999** | **-12.92652** | **23.64943** | | **-0.38563** | | **-12.70678** | | **23.99881** |  |  |  |  |  |  |
| H | **-1.96284** | | **-14.32941** | | | **23.6998** | **-3.17548** | **-13.91947** | **23.67428** | | **-3.02786** | | **-13.70218** | | **23.92684** |  |  |  |  |  |  |
| H | **-4.0663** | | **-11.338** | | | **23.87262** | **-4.07413** | **-11.55672** | **23.65295** | | **-4.00197** | | **-11.33966** | | **23.82868** |  |  |  |  |  |  |
| H | **-3.08611** | | **-11.02484** | | | **17.09452** | **-3.37034** | **-10.59547** | **17.03491** | | **-3.11313** | | **-10.99967** | | **17.08764** |  |  |  |  |  |  |
| H | **-4.52747** | | **-10.93464** | | | **18.11944** | **-4.71355** | **-10.65171** | **18.18842** | | **-4.5386** | | **-10.95081** | | **18.13801** |  |  |  |  |  |  |
| H | **-3.96041** | | **-14.31839** | | | **15.82249** | **-4.28501** | **-13.71984** | **15.4884** | | **-3.94219** | | **-14.29107** | | **15.78602** |  |  |  |  |  |  |
| H | **-2.57588** | | **-13.32953** | | | **16.3026** | **-2.9518** | **-12.62329** | **15.80285** | | **-2.57363** | | **-13.27107** | | **16.24419** |  |  |  |  |  |  |
| H | **-8.03734** | | **-11.8093** | | | **21.07042** | **-7.65762** | **-10.77714** | **19.09405** | | **-7.95053** | | **-11.92375** | | **21.1534** |  |  |  |  |  |  |
| H | **-6.66176** | | **-12.22736** | | | **18.50755** | **-6.34417** | **-12.56479** | **19.14856** | | **-6.61285** | | **-12.29914** | | **18.56532** |  |  |  |  |  |  |
| H | **-9.14996** | | **-10.61242** | | | **17.05657** | **-5.21759** | **-14.35209** | **19.82343** | | **-9.15651** | | **-10.71823** | | **17.17755** |  |  |  |  |  |  |
| H | **-7.83582** | | **-9.43977** | | | **17.00331** | **-4.30707** | **-14.0461** | **21.33461** | | **-7.86444** | | **-9.5227** | | **17.10064** |  |  |  |  |  |  |
| H | **-7.49273** | | **-11.17424** | | | **16.71872** | **-5.81572** | **-14.95506** | **21.38363** | | **-7.49789** | | **-11.25001** | | **16.80039** |  |  |  |  |  |  |
| H | **-7.58853** | | **-14.10356** | | | **20.2676** | **-9.29678** | **-12.39885** | **19.93126** | | **-7.46834** | | **-14.20012** | | **20.31345** |  |  |  |  |  |  |
| H | **-6.72033** | | **-13.82704** | | | **21.7959** | **-9.64921** | **-10.78467** | **20.59883** | | **-6.60108** | | **-13.92598** | | **21.84125** |  |  |  |  |  |  |
| H | **-5.83883** | | **-13.81002** | | | **20.26817** | **-8.8794** | **-12.01267** | **21.61782** | | **-5.72428** | | **-13.87703** | | **20.31027** |  |  |  |  |  |  |

# Fig. S27. The four possible diastereomers (**2a**, **2b**, **2c** and **2d**).

# Table S13. Experimental and Calculated ^13^C NMR Chemical Shifts of **2**a–**2**d

| No. | *δ*_exp_ ^a^ |  | *σ*^x^ (shielding constants)^b^ | | | |  | *δ*_s_ (scaled shifts)^d^ | | | |
| --- | --- | --- | --- | --- | --- | --- | --- | --- | --- | --- | --- |
|  |  |  | **2a** | **2b** | **2c** | **2d** |  | **2a** | **2b** | **2c** | **2d** |
| 1 | 165.5 |  | 6.56 | 4.97 | 4.74 | 3.86 |  | 169.29 | 170.57 | 170.92 | 171.79 |
| 2 | 48.7 |  | 133.77 | 129.75 | 129.59 | 131.29 |  | 47.88 | 51.03 | 51.73 | 49.73 |
| 3 | 172.3 |  | 5.57 | 5.91 | 6.55 | 8.02 |  | 170.23 | 169.67 | 169.20 | 167.81 |
| 4 | 49.7 |  | 130.82 | 131.81 | 129.58 | 131.75 |  | 50.70 | 49.06 | 51.74 | 49.29 |
| 5 | 34.3 |  | 152.45 | 146.00 | 143.75 | 147.91 |  | 30.05 | 35.46 | 38.20 | 33.81 |
| 6 | 17.3 |  | 167.22 | 170.49 | 171.58 | 169.63 |  | 15.96 | 12.00 | 11.64 | 13.00 |
| 7 | 171.6 |  | 6.21 | 2.89 | 3.88 | 8.53 |  | 169.62 | 172.57 | 171.75 | 167.31 |
| 8 | 57.6 |  | 125.56 | 114.85 | 122.88 | 121.44 |  | 55.72 | 65.30 | 58.13 | 59.16 |
| 9 | 29.8 |  | 151.51 | 154.61 | 155.55 | 151.74 |  | 30.95 | 27.22 | 26.94 | 30.14 |
| 10 | 18.3 |  | 166.88 | 167.28 | 167.09 | 167.43 |  | 16.28 | 15.08 | 15.92 | 15.11 |
| 11 | 17.3 |  | 169.04 | 167.23 | 168.23 | 167.20 |  | 14.22 | 15.12 | 14.83 | 15.34 |
| 12 | 166.4 |  | 4.24 | 3.46 | 2.30 | 0.62 |  | 171.50 | 172.02 | 173.26 | 174.90 |
| 13 | 55 |  | 122.94 | 125.71 | 130.68 | 123.25 |  | 58.21 | 54.90 | 50.68 | 57.43 |
| 14 | 41.6 |  | 135.99 | 135.45 | 129.23 | 138.25 |  | 45.76 | 45.57 | 52.07 | 43.06 |
| 15 | 169.8 |  | 5.42 | 5.49 | 2.09 | 3.56 |  | 170.37 | 170.07 | 173.45 | 172.08 |
| 16 | 62 |  | 117.30 | 115.82 | 117.78 | 119.62 |  | 63.60 | 64.38 | 63.00 | 60.91 |
| 17 | 171.4 |  | 8.10 | 9.21 | 9.83 | 6.71 |  | 167.81 | 166.51 | 166.07 | 169.06 |
| 18 | 49.7 |  | 123.47 | 127.84 | 130.42 | 128.68 |  | 57.71 | 52.86 | 50.93 | 52.23 |
| 19 | 167 |  | 13.33 | 11.50 | 14.16 | 12.90 |  | 162.82 | 164.32 | 161.93 | 163.13 |
| 20 | 43 |  | 141.28 | 139.15 | 140.58 | 140.25 |  | 40.71 | 42.02 | 41.24 | 41.14 |
| 21 | 99.3 |  | 80.49 | 75.56 | 80.73 | 76.19 |  | 98.72 | 102.95 | 98.37 | 102.50 |
| 22 | 132.8 |  | 46.13 | 45.82 | 45.25 | 53.05 |  | 131.52 | 131.44 | 132.25 | 124.68 |
| 23 | 59.8 |  | 120.48 | 121.80 | 118.64 | 116.09 |  | 60.56 | 58.65 | 62.19 | 64.29 |
| 24 | 35.8 |  | 147.91 | 148.65 | 148.72 | 143.50 |  | 34.39 | 32.93 | 33.46 | 38.04 |
| 25 | 171.54 |  | 4.93 | 6.10 | 4.42 | 3.26 |  | 170.85 | 169.49 | 171.23 | 172.37 |
| 26 | 172.68 |  | 0.89 | 6.58 | 6.67 | 3.77 |  | 174.69 | 169.04 | 169.08 | 171.88 |
| R^2^ |  |  |  |  |  |  |  | 0.9977 | 0.9971 | 0.9960 | 0.9966 |
| Slope (a) |  |  |  |  |  |  |  | 1.0478 | 1.0438 | 1.0474 | 1.0440 |
| Intercept (b) |  |  |  |  |  |  |  | 2.3160 | 3.2394 | 2.4852 | 3.0454 |
| CAME |  |  |  |  |  |  |  | 1.4284 | 1.6495 | 1.8486 | 1.6907 |

# Table S14. Experimental and Calculated ^1^H NMR Chemical Shifts of **2**a–**2**d

| No. | *δ*_exp_ ^a^ |  | *σ*^x^ (shielding constants)^b^ | | | |  | *δ*_s_ (scaled shifts)^d^ | | | |
| --- | --- | --- | --- | --- | --- | --- | --- | --- | --- | --- | --- |
|  |  |  | **2a** | **2b** | **2c** | **2d** |  | **2a** | **2b** | **2c** | **2d** |
| 1 | 4.24 |  | 26.786 | 27.600 | 28.000 | 27.554 |  | 4.5579 | 3.9122 | 3.3903 | 3.8391 |
| 2 | 1.20 |  | 30.520 | 30.280 | 30.165 | 30.248 |  | 0.8791 | 1.2007 | 1.1388 | 1.0938 |
| 3 | 1.20 |  | 30.520 | 30.280 | 30.165 | 30.248 |  | 0.8791 | 1.2007 | 1.1388 | 1.0938 |
| 4 | 1.20 |  | 30.520 | 30.280 | 30.165 | 30.248 |  | 0.8791 | 1.2007 | 1.1388 | 1.0938 |
| 5 | 4.27 |  | 27.437 | 27.316 | 26.868 | 26.540 |  | 3.9166 | 4.1995 | 4.5675 | 4.8724 |
| 6 | 2.50 |  | 28.552 | 29.022 | 26.931 | 28.705 |  | 2.8180 | 2.4735 | 4.5020 | 2.6662 |
| 7 | 2.27 |  | 29.690 | 29.599 | 29.016 | 29.285 |  | 1.6968 | 1.8897 | 2.3337 | 2.0751 |
| 8 | 4.06 |  | 26.776 | 28.298 | 27.697 | 27.194 |  | 4.5678 | 3.2060 | 3.7054 | 4.2060 |
| 9 | 2.06 |  | 29.123 | 29.066 | 29.172 | 29.593 |  | 2.2555 | 2.4290 | 2.1715 | 1.7612 |
| 10 | 0.84 |  | 30.686 | 30.833 | 30.750 | 30.747 |  | 0.7156 | 0.6412 | 0.5305 | 0.5852 |
| 11 | 0.84 |  | 30.686 | 30.833 | 30.750 | 30.747 |  | 0.7156 | 0.6412 | 0.5305 | 0.5852 |
| 12 | 0.84 |  | 30.686 | 30.833 | 30.750 | 30.747 |  | 0.7156 | 0.6412 | 0.5305 | 0.5852 |
| 13 | 0.78 |  | 30.662 | 30.843 | 30.766 | 30.766 |  | 0.7392 | 0.6311 | 0.5138 | 0.5659 |
| 14 | 0.78 |  | 30.662 | 30.843 | 30.766 | 30.766 |  | 0.7392 | 0.6311 | 0.5138 | 0.5659 |
| 15 | 0.78 |  | 30.662 | 30.843 | 30.766 | 30.766 |  | 0.7392 | 0.6311 | 0.5138 | 0.5659 |
| 16 | 3.86 |  | 28.405 | 28.775 | 28.644 | 28.159 |  | 2.9629 | 2.7234 | 2.7206 | 3.2226 |
| 17 | 3.69 |  | 28.084 | 28.251 | 28.274 | 27.803 |  | 3.2791 | 3.2535 | 3.1053 | 3.5853 |
| 18 | 3.82 |  | 27.276 | 27.426 | 27.939 | 27.665 |  | 4.0752 | 4.0882 | 3.4537 | 3.7260 |
| 19 | 2.07 |  | 27.472 | 28.957 | 28.127 | 28.645 |  | 3.8821 | 2.5393 | 3.2582 | 2.7273 |
| 20 | 1.96 |  | 29.579 | 28.459 | 28.203 | 27.666 |  | 1.8062 | 3.0431 | 3.1792 | 3.7250 |
| 21 | 4.71 |  | 27.119 | 26.711 | 26.514 | 27.713 |  | 4.2299 | 4.8116 | 4.9356 | 3.6771 |
| 22 | 2.76 |  | 28.038 | 28.443 | 28.855 | 27.837 |  | 3.3244 | 3.0593 | 2.5011 | 3.5507 |
| 23 | 2.25 |  | 28.944 | 28.732 | 29.255 | 29.303 |  | 2.4318 | 2.7669 | 2.0852 | 2.0568 |
| 24 | 3.53 |  | 28.156 | 26.715 | 28.175 | 28.066 |  | 3.2082 | 4.8076 | 3.2083 | 3.3173 |
| 25 | 3.94 |  | 26.759 | 28.355 | 26.916 | 26.652 |  | 4.5845 | 3.1483 | 4.5176 | 4.7583 |
| 26 | 5.40 |  | 26.096 | 25.747 | 26.126 | 26.072 |  | 5.2377 | 5.7869 | 5.3391 | 5.3493 |
| 27 | 7.19 |  | 24.103 | 24.071 | 24.036 | 24.264 |  | 7.2013 | 7.4826 | 7.5126 | 7.1918 |
| R^2^ |  |  |  |  |  |  |  | 0.9160 | 0.9122 | 0.8722 | 0.9081 |
| Slope (a) |  |  |  |  |  |  |  | 1.0150 | 0.9884 | 0.9616 | 0.9813 |
| Intercept (b) |  |  |  |  |  |  |  | 0.4397 | 0.3852 | 0.5919 | 0.5307 |
| CAME |  |  |  |  |  |  |  | 0.2186 | 0.2303 | 0.2732 | 0.2248 |

# Table S15. DP4+ Probabilities Computed for **2**a-**2**d

|  | **2a** | **2b** | **2c** | **2d** |
| --- | --- | --- | --- | --- |
| DP4+ (H data) | 83.93% | 2.54% | 0.00% | 13.53% |
| DP4+ (C data) | 100.00% | 0.00% | 0.00% | 0.00% |
| DP4+ (all data) | 100.00% | 0.00% | 0.00% | 0.00% |


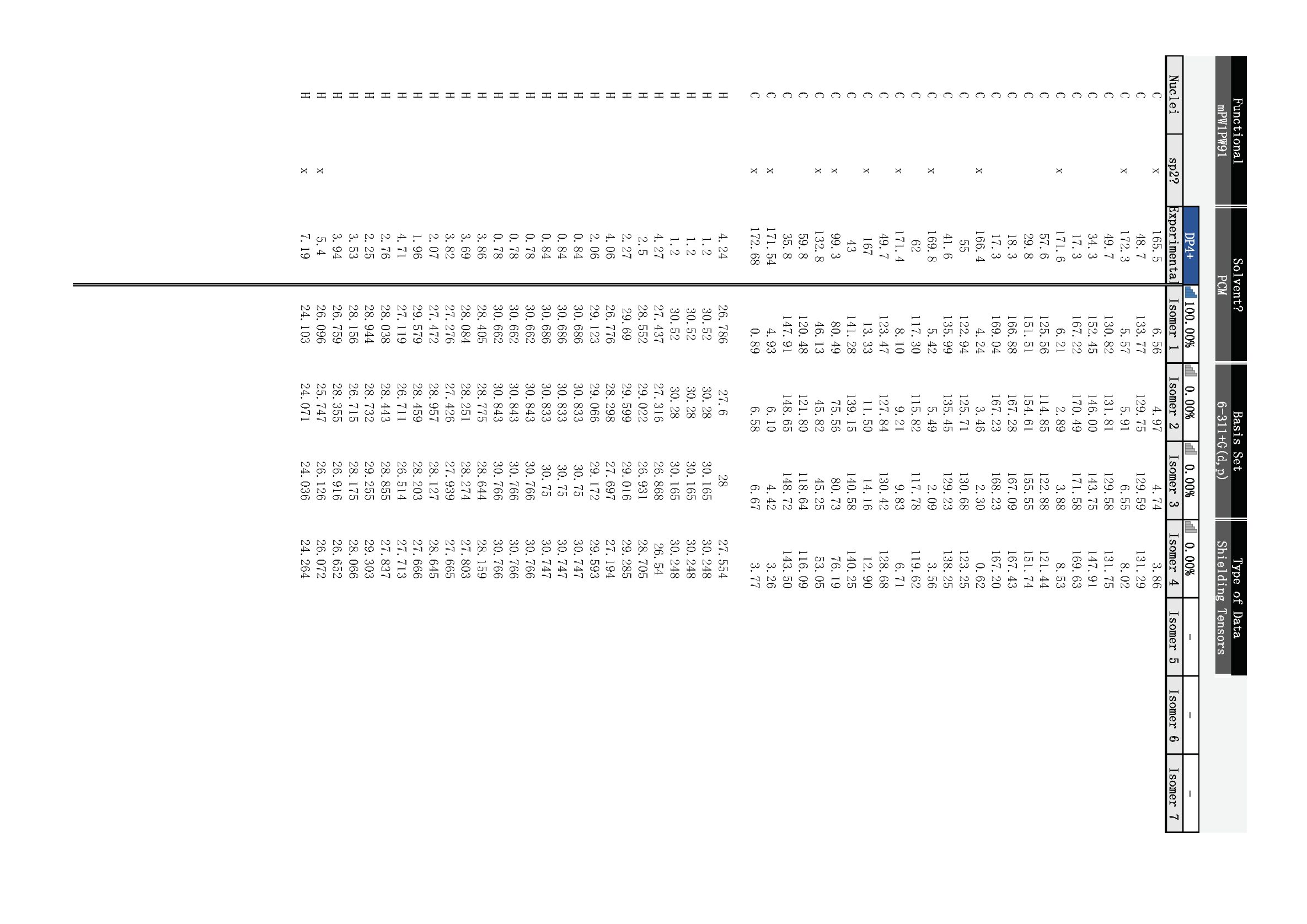


# Table S16. Lowest-energy Conformers Optimized at the M062X/6-311+G (d, p) Level of **2**a with Relative Energies < 3.0 kcal/mol

| **2a_C1** | G 2773.5926 Ha  Boltzman pop. 2.55% | **2a_C2** | G 2773.5942 Ha  Boltzman pop. 20.38% | **2a_C3** | G 2773.5933 Ha  Boltzman pop. 16.13% |
| --- | --- | --- | --- | --- | --- |
| 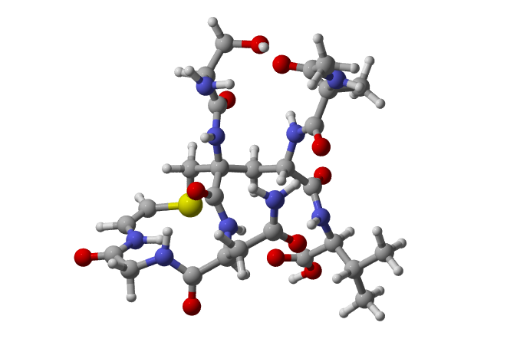 | | 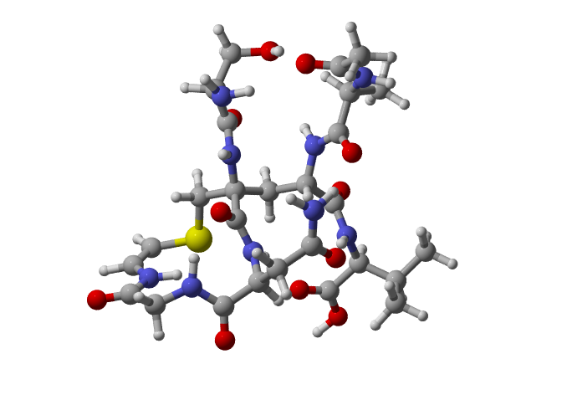 | | 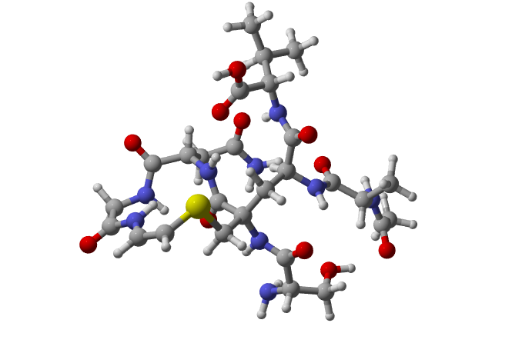 | |
| **1c_C4** | G 2773.5942 Ha  Boltzman pop. 20.33% | **1c_C5** | G 2773.5943 Ha  Boltzman pop. 20.34% | **1c_C6** | G 2773.5943 Ha  Boltzman pop. 20.27% |
| 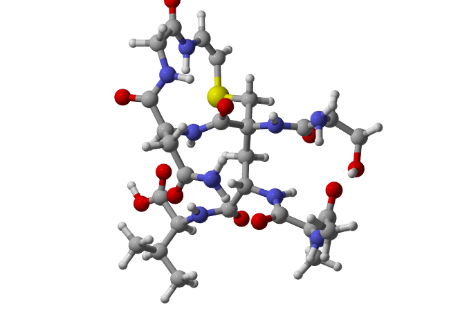 | | 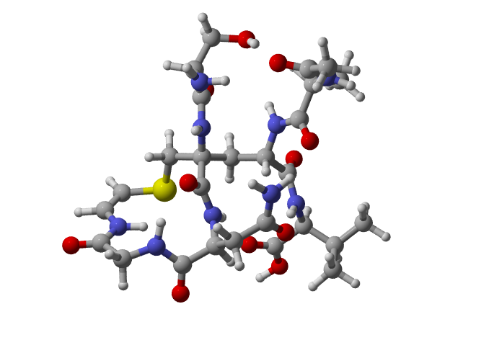 | | 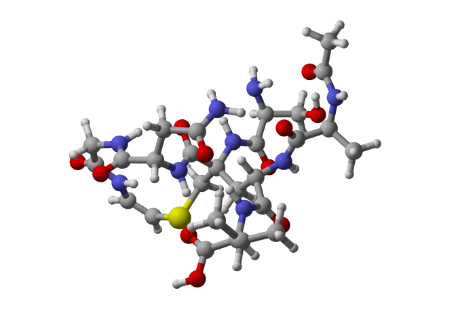 | |

# Table S17. Atomic Coordinates for the Lowest-energy Conformers of **2a** (**2a_C1**–**2a_C6**)

| **Atoms** | **2a_C1** | | | **2a_C2** | | | **2a_C3** | | |
| --- | --- | --- | --- | --- | --- | --- | --- | --- | --- |
|  | **x** | **y** | **z** | **x** | **y** | **z** | **x** | **y** | **z** |
| **C** | **-5.89246** | **-7.9527** | **3.45298** | **-5.95885** | **-7.93696** | **3.55348** | **-4.91497** | **-8.06092** | **3.02474** |
| **C** | **-6.0689** | **-9.36973** | **2.94736** | **-6.11051** | **-9.35694** | **3.04786** | **-5.1574** | **-9.45085** | **2.47123** |
| **N** | **-5.1586** | **-10.2797** | **3.37333** | **-5.20953** | **-10.25984** | **3.50709** | **-5.11318** | **-10.46746** | **3.36436** |
| **C** | **-5.27102** | **-11.7159** | **3.14752** | **-5.30383** | **-11.69739** | **3.28113** | **-5.33614** | **-11.86444** | **3.02151** |
| **C** | **-6.29228** | **-12.3268** | **4.12949** | **-6.3428** | **-12.31603** | **4.23916** | **-6.26955** | **-12.47084** | **4.07934** |
| **N** | **-6.82756** | **-13.5109** | **3.75433** | **-6.86778** | **-13.49843** | **3.84528** | **-6.86852** | **-13.62616** | **3.73001** |
| **C** | **-7.64735** | **-14.301** | **4.66514** | **-7.68588** | **-14.31146** | **4.73622** | **-7.69375** | **-14.37952** | **4.66226** |
| **C** | **-8.64606** | **-15.2284** | **3.92791** | **-8.66556** | **-15.23883** | **3.97174** | **-8.69961** | **-15.2992** | **3.92625** |
| **O** | **-6.9953** | **-9.6525** | **2.17215** | **-7.00925** | **-9.64768** | **2.24348** | **-5.36918** | **-9.62637** | **1.26088** |
| **C** | **-3.89441** | **-12.3741** | **3.33432** | **-3.92695** | **-12.34392** | **3.50369** | **-4.00825** | **-12.64407** | **2.94521** |
| **O** | **-6.55354** | **-11.7713** | **5.20424** | **-6.62568** | **-11.76643** | **5.31147** | **-6.40799** | **-11.93656** | **5.19151** |
| **C** | **-6.7842** | **-15.2268** | **5.54903** | **-6.81745** | **-15.24939** | **5.60056** | **-6.84842** | **-15.314** | **5.55652** |
| **N** | **-7.04558** | **-15.1405** | **6.87995** | **-7.15029** | **-15.27307** | **6.92012** | **-7.07124** | **-15.17557** | **6.8903** |
| **O** | **-6.01014** | **-16.0398** | **5.04761** | **-5.97743** | **-15.98325** | **5.08394** | **-6.12743** | **-16.17995** | **5.06607** |
| **C** | **-6.7904** | **-16.2513** | **7.77891** | **-6.88226** | **-16.43627** | **7.74178** | **-6.83819** | **-16.2736** | **7.81033** |
| **C** | **-6.64527** | **-15.7421** | **9.24366** | **-6.217** | **-16.12405** | **9.11198** | **-6.6966** | **-15.73898** | **9.26632** |
| **C** | **-6.52212** | **-16.8793** | **10.26886** | **-4.86519** | **-15.4295** | **8.89471** | **-6.59647** | **-16.85764** | **10.31416** |
| **C** | **-5.43742** | **-14.796** | **9.3416** | **-7.12378** | **-15.31439** | **10.05155** | **-5.47569** | **-14.80844** | **9.35489** |
| **N** | **-9.81117** | **-13.4668** | **2.59557** | **-9.80133** | **-13.48771** | **2.59774** | **-9.90985** | **-13.54171** | **2.569** |
| **C** | **-9.17219** | **-13.5254** | **1.39765** | **-9.12673** | **-13.5458** | **1.41977** | **-9.14736** | **-13.47186** | **1.45292** |
| **O** | **-8.53589** | **-14.5039** | **0.99784** | **-8.47089** | **-14.52061** | **1.04304** | **-8.21682** | **-14.23968** | **1.17375** |
| **C** | **-9.34824** | **-12.2721** | **0.51299** | **-9.28686** | **-12.29744** | **0.5251** | **-9.54077** | **-12.34184** | **0.46986** |
| **O** | **-11.5486** | **-13.0366** | **4.51889** | **-11.59325** | **-13.05753** | **4.46809** | **-11.67325** | **-13.17662** | **4.46531** |
| **C** | **-10.8874** | **-15.6396** | **2.75155** | **-10.86718** | **-15.66682** | **2.72995** | **-10.90456** | **-15.73846** | **2.72133** |
| **C** | **-10.8112** | **-14.0202** | **4.70606** | **-10.86049** | **-14.03975** | **4.67877** | **-10.88326** | **-14.11217** | **4.68064** |
| **C** | **-9.99483** | **-14.5998** | **3.50876** | **-10.00441** | **-14.61781** | **3.50976** | **-10.0441** | **-14.67934** | **3.48955** |
| **O** | **-13.3137** | **-15.1653** | **8.18922** | **-13.46469** | **-15.16788** | **8.0956** | **-13.27927** | **-15.14699** | **8.27014** |
| **C** | **-12.8728** | **-14.5404** | **7.23098** | **-12.99435** | **-14.55017** | **7.14651** | **-12.87323** | **-14.55741** | **7.27466** |
| **C** | **-11.3707** | **-14.1809** | **7.15623** | **-11.48955** | **-14.19751** | **7.1116** | **-11.37768** | **-14.18369** | **7.14717** |
| **N** | **-10.7195** | **-14.6287** | **5.9068** | **-10.80812** | **-14.65031** | **5.8805** | **-10.74725** | **-14.67016** | **5.9011** |
| **O** | **-16.329** | **-15.8189** | **4.41473** | **-16.35502** | **-15.85774** | **4.23349** | **-16.37151** | **-15.93954** | **4.53396** |
| **C** | **-15.2208** | **-15.5914** | **4.87755** | **-15.26246** | **-15.62111** | **4.72777** | **-15.25775** | **-15.69767** | **4.97485** |
| **C** | **-15.0127** | **-14.5521** | **5.99044** | **-15.09388** | **-14.57308** | **5.83929** | **-15.04001** | **-14.65883** | **6.08566** |
| **N** | **-13.6341** | **-14.1354** | **6.17134** | **-13.72319** | **-14.1512** | **6.06214** | **-13.66648** | **-14.21001** | **6.21893** |
| **N** | **-14.0724** | **-16.222** | **4.45662** | **-14.09877** | **-16.24852** | **4.34514** | **-14.10966** | **-16.31231** | **4.52589** |
| **S** | **-11.251** | **-17.2176** | **3.67213** | **-11.25054** | **-17.24306** | **3.64548** | **-11.29645** | **-17.29792** | **3.6606** |
| **C** | **-12.8372** | **-17.6561** | **2.9758** | **-12.81663** | **-17.68921** | **2.90975** | **-12.899** | **-17.71664** | **2.99128** |
| **C** | **-13.9996** | **-17.153** | **3.43551** | **-13.99288** | **-17.18631** | **3.33309** | **-14.05128** | **-17.22558** | **3.48887** |
| **C** | **-8.11936** | **-12.0635** | **-0.39051** | **-8.03365** | **-12.08128** | **-0.34243** | **-8.29841** | **-11.56859** | **0.01114** |
| **O** | **-6.93381** | **-11.7615** | **0.32829** | **-6.87311** | **-11.76364** | **0.40971** | **-7.91189** | **-10.73556** | **1.09602** |
| **N** | **-9.76927** | **-11.1136** | **1.30836** | **-9.74153** | **-11.13963** | **1.30268** | **-10.5884** | **-11.47629** | **1.02057** |
| **C** | **-11.1065** | **-12.6936** | **7.49015** | **-11.22205** | **-12.71199** | **7.44782** | **-11.10864** | **-12.68411** | **7.41457** |
| **C** | **-9.60802** | **-12.4255** | **7.40942** | **-9.71981** | **-12.45588** | **7.39264** | **-9.6043** | **-12.43688** | **7.35289** |
| **O** | **-8.81068** | **-13.1073** | **8.06625** | **-8.93662** | **-13.17198** | **8.02907** | **-8.82859** | **-13.11123** | **8.0412** |
| **N** | **-9.20669** | **-11.4533** | **6.56148** | **-9.29879** | **-11.4527** | **6.59216** | **-9.1696** | **-11.4943** | **6.48687** |
| **C** | **-7.93632** | **-17.259** | **7.60183** | **-8.18121** | **-17.23929** | **7.87271** | **-7.99501** | **-17.27013** | **7.64077** |
| **O** | **-9.05529** | **-16.9815** | **7.2054** | **-9.24673** | **-16.95033** | **7.3579** | **-9.10458** | **-16.98809** | **7.22046** |
| **O** | **-7.58059** | **-18.5176** | **7.91573** | **-8.02404** | **-18.35236** | **8.61532** | **-7.66212** | **-18.52487** | **7.99071** |
| **H** | **-6.76339** | **-7.68928** | **4.06069** | **-6.85342** | **-7.67403** | **4.12599** | **-4.03449** | **-7.63918** | **2.53139** |
| **H** | **-4.98752** | **-7.81119** | **4.04914** | **-5.07788** | **-7.7892** | **4.18305** | **-5.76868** | **-7.42719** | **2.769** |
| **H** | **-5.87054** | **-7.27435** | **2.59618** | **-5.90661** | **-7.2624** | **2.69495** | **-4.76086** | **-8.04296** | **4.10677** |
| **H** | **-4.54971** | **-9.99544** | **4.12861** | **-4.62746** | **-9.96961** | **4.28102** | **-5.0874** | **-10.26408** | **4.35636** |
| **H** | **-5.61766** | **-11.8795** | **2.12449** | **-5.62328** | **-11.86543** | **2.25008** | **-5.83143** | **-11.89058** | **2.04915** |
| **H** | **-6.59577** | **-13.9029** | **2.84914** | **-6.6035** | **-13.88821** | **2.94803** | **-6.82407** | **-13.95279** | **2.769** |
| **H** | **-8.17745** | **-13.5901** | **5.29806** | **-8.22703** | **-13.62088** | **5.38201** | **-8.2165** | **-13.6576** | **5.28966** |
| **H** | **-8.16789** | **-15.6389** | **3.03642** | **-8.16009** | **-15.64879** | **3.09502** | **-8.21036** | **-15.73054** | **3.05171** |
| **H** | **-8.85128** | **-16.0897** | **4.57099** | **-8.88715** | **-16.09892** | **4.61129** | **-8.92431** | **-16.14882** | **4.57798** |
| **H** | **-3.95672** | **-13.4525** | **3.17003** | **-3.97726** | **-13.42359** | **3.34352** | **-4.18205** | **-13.68974** | **2.67609** |
| **H** | **-3.18077** | **-11.9551** | **2.61951** | **-3.19982** | **-11.92241** | **2.80407** | **-3.36446** | **-12.18967** | **2.18732** |
| **H** | **-3.51645** | **-12.2128** | **4.35041** | **-3.57477** | **-12.17477** | **4.52772** | **-3.48878** | **-12.61933** | **3.90844** |
| **H** | **-7.67906** | **-14.4156** | **7.21866** | **-7.80957** | **-14.57957** | **7.27327** | **-7.67513** | **-14.42284** | **7.22094** |
| **H** | **-5.86697** | **-16.7505** | **7.47004** | **-6.19028** | **-17.0658** | **7.16962** | **-5.91959** | **-16.7921** | **7.51905** |
| **H** | **-7.55174** | **-15.1657** | **9.47148** | **-6.0303** | **-17.10071** | **9.57422** | **-7.59635** | **-15.14574** | **9.47691** |
| **H** | **-6.36933** | **-16.4547** | **11.26623** | **-4.35741** | **-15.28577** | **9.85423** | **-6.44338** | **-16.41554** | **11.30385** |
| **H** | **-7.41479** | **-17.5088** | **10.31113** | **-4.20813** | **-16.02091** | **8.24786** | **-7.49857** | **-17.47298** | **10.36321** |
| **H** | **-5.66673** | **-17.5274** | **10.04581** | **-4.99768** | **-14.44618** | **8.43198** | **-5.74936** | **-17.52232** | **10.10932** |
| **H** | **-5.51727** | **-13.96** | **8.64262** | **-6.62115** | **-15.16197** | **11.0125** | **-5.40719** | **-14.37817** | **10.35932** |
| **H** | **-4.50441** | **-15.3329** | **9.13036** | **-8.06792** | **-15.83211** | **10.25594** | **-5.53633** | **-13.98584** | **8.63842** |
| **H** | **-5.36699** | **-14.3849** | **10.35391** | **-7.365** | **-14.3301** | **9.63918** | **-4.54922** | **-15.36335** | **9.16228** |
| **H** | **-10.3763** | **-12.6362** | **2.76231** | **-10.37523** | **-12.65959** | **2.7462** | **-10.67129** | **-12.86116** | **2.59896** |
| **H** | **-10.1861** | **-12.5256** | **-0.1539** | **-10.10222** | **-12.56105** | **-0.16536** | **-9.94299** | **-12.8775** | **-0.39963** |
| **H** | **-11.8427** | **-15.1655** | **2.51727** | **-11.81732** | **-15.19911** | **2.46416** | **-11.85351** | **-15.2768** | **2.44185** |
| **H** | **-10.3734** | **-15.8909** | **1.8231** | **-10.32335** | **-15.91896** | **1.81883** | **-10.35701** | **-16.00332** | **1.81375** |
| **H** | **-10.9053** | **-14.7805** | **7.93798** | **-11.04758** | **-14.79851** | **7.90557** | **-10.88851** | **-14.74842** | **7.94037** |
| **H** | **-10.1433** | **-15.458** | **6.01687** | **-10.23266** | **-15.47701** | **6.00911** | **-10.14097** | **-15.4746** | **6.03467** |
| **H** | **-15.3646** | **-14.9832** | **6.9323** | **-15.4756** | **-14.9984** | **6.77209** | **-15.34698** | **-15.10099** | **7.03818** |
| **H** | **-15.6527** | **-13.6983** | **5.75061** | **-15.72803** | **-13.72309** | **5.57225** | **-15.71116** | **-13.82217** | **5.8715** |
| **H** | **-13.183** | **-13.5639** | **5.45849** | **-13.25027** | **-13.58375** | **5.3601** | **-13.23991** | **-13.66235** | **5.4723** |
| **H** | **-13.1997** | **-15.9868** | **4.9163** | **-13.24115** | **-16.00599** | **4.82875** | **-13.23016** | **-16.06675** | **4.96668** |
| **H** | **-12.8446** | **-18.389** | **2.1771** | **-12.80032** | **-18.42752** | **2.1162** | **-12.92683** | **-18.43631** | **2.18103** |
| **H** | **-14.9583** | **-17.4363** | **3.01303** | **-14.93813** | **-17.47568** | **2.88516** | **-15.01786** | **-17.508** | **3.08379** |
| **H** | **-7.92017** | **-12.9852** | **-0.94124** | **-7.80898** | **-13.00367** | **-0.88196** | **-7.50731** | **-12.27653** | **-0.263** |
| **H** | **-8.35268** | **-11.2661** | **-1.11279** | **-8.2531** | **-11.28991** | **-1.07566** | **-8.55774** | **-10.96638** | **-0.87251** |
| **H** | **-7.02696** | **-10.9149** | **0.80419** | **-6.98876** | **-10.91561** | **0.87801** | **-7.04365** | **-10.31783** | **0.92744** |
| **H** | **-8.97921** | **-10.6583** | **1.76384** | **-8.9696** | **-10.67566** | **1.77995** | **-10.15935** | **-10.64722** | **1.42702** |
| **H** | **-10.227** | **-10.4193** | **0.72585** | **-10.18782** | **-10.45163** | **0.70407** | **-11.23823** | **-11.1745** | **0.3024** |
| **H** | **-11.6715** | **-12.0289** | **6.83539** | **-11.76977** | **-12.04369** | **6.78193** | **-11.65432** | **-12.04726** | **6.71665** |
| **H** | **-11.4327** | **-12.5276** | **8.52214** | **-11.56602** | **-12.54059** | **8.47334** | **-11.45236** | **-12.46394** | **8.43074** |
| **H** | **-8.21681** | **-11.3848** | **6.32509** | **-8.30471** | **-11.38915** | **6.37077** | **-8.17209** | **-11.43401** | **6.28608** |
| **H** | **-9.86984** | **-11.0257** | **5.93162** | **-9.9491** | **-10.99324** | **5.97164** | **-9.80993** | **-11.05183** | **5.84427** |
| **H** | **-8.35627** | **-19.0881** | **7.77255** | **-8.87868** | **-18.81744** | **8.63139** | **-8.44335** | **-19.08818** | **7.84956** |
| **Atoms** | **2a_C4** | | | **2a_C5** | | | **2a_C6** | | |
|  | **x** | **y** | **z** | **x** | **y** | **z** | **x** | **y** | **z** |
| **C** | **-5.98233** | **-7.92582** | **3.59128** | **-5.96429** | **-7.91321** | **3.58634** | **-5.89265** | **-7.91846** | **3.55357** |
| **C** | **-6.12113** | **-9.34837** | **3.08924** | **-6.12311** | **-9.32405** | **3.05783** | **-6.07822** | **-9.32061** | **3.01086** |
| **N** | **-5.22679** | **-10.24705** | **3.56923** | **-5.20964** | **-10.23239** | **3.48015** | **-5.16514** | **-10.2437** | **3.40081** |
| **C** | **-5.31378** | **-11.68584** | **3.34821** | **-5.30474** | **-11.66572** | **3.22914** | **-5.28108** | **-11.67258** | **3.13369** |
| **C** | **-6.3698** | **-12.30221** | **4.28891** | **-6.31987** | **-12.30588** | **4.19847** | **-6.2803** | **-12.31491** | **4.11799** |
| **N** | **-6.8841** | **-13.48797** | **3.89097** | **-6.85108** | **-13.48179** | **3.79373** | **-6.83353** | **-13.47959** | **3.71036** |
| **C** | **-7.71808** | **-14.29814** | **4.76973** | **-7.64709** | **-14.31376** | **4.68715** | **-7.61861** | **-14.31429** | **4.61085** |
| **C** | **-8.68097** | **-15.23114** | **3.99084** | **-8.6411** | **-15.2297** | **3.92743** | **-8.6393** | **-15.20972** | **3.86212** |
| **O** | **-7.00397** | **-9.64488** | **2.26951** | **-7.03853** | **-9.60268** | **2.26812** | **-7.01415** | **-9.5796** | **2.23869** |
| **C** | **-3.94009** | **-12.3283** | **3.60026** | **-3.92129** | **-12.31174** | **3.40815** | **-3.90079** | **-12.33507** | **3.27208** |
| **O** | **-6.67505** | **-11.74781** | **5.35258** | **-6.57925** | **-11.77814** | **5.28758** | **-6.50861** | **-11.79914** | **5.21972** |
| **C** | **-6.86516** | **-15.23012** | **5.65561** | **-6.75708** | **-15.26494** | **5.51436** | **-6.7211** | **-15.28558** | **5.40602** |
| **N** | **-7.22445** | **-15.24835** | **6.96831** | **-7.05976** | **-15.3138** | **6.8405** | **-6.99471** | **-15.34824** | **6.7379** |
| **O** | **-6.0135** | **-15.96465** | **5.15941** | **-5.92744** | **-15.98704** | **4.96543** | **-5.91206** | **-16.00964** | **4.82961** |
| **C** | **-6.97038** | **-16.4069** | **7.80087** | **-6.77091** | **-16.49153** | **7.63396** | **-6.70207** | **-16.53951** | **7.50946** |
| **C** | **-6.33221** | **-16.08662** | **9.18207** | **-6.07561** | **-16.2035** | **8.99447** | **-5.97319** | **-16.2775** | **8.8576** |
| **C** | **-4.97801** | **-15.39001** | **8.98746** | **-4.7298** | **-15.50292** | **8.7599** | **-4.62445** | **-15.59008** | **8.60184** |
| **C** | **-7.2586** | **-15.2745** | **10.10015** | **-6.96224** | **-15.41275** | **9.96873** | **-6.8281** | **-15.489** | **9.86155** |
| **N** | **-9.79305** | **-13.48903** | **2.58626** | **-9.81209** | **-13.45658** | **2.61246** | **-9.81966** | **-13.40745** | **2.59592** |
| **C** | **-9.095** | **-13.55122** | **1.42227** | **-9.16428** | **-13.49085** | **1.41863** | **-9.19893** | **-13.43357** | **1.38758** |
| **O** | **-8.42935** | **-14.52617** | **1.06353** | **-8.51475** | **-14.45666** | **1.00916** | **-8.56887** | **-14.40085** | **0.95195** |
| **C** | **-9.24045** | **-12.3077** | **0.5184** | **-9.34746** | **-12.22659** | **0.55108** | **-9.38782** | **-12.15683** | **0.53976** |
| **O** | **-11.62195** | **-13.05336** | **4.41904** | **-11.5635** | **-13.06774** | **4.5299** | **-11.52334** | **-13.02396** | **4.55679** |
| **C** | **-10.85675** | **-15.6698** | **2.70818** | **-10.86914** | **-15.64097** | **2.7276** | **-10.89788** | **-15.58151** | **2.70736** |
| **C** | **-10.89166** | **-14.03316** | **4.64879** | **-10.82287** | **-14.05087** | **4.70597** | **-10.78998** | **-14.01724** | **4.70408** |
| **C** | **-10.01167** | **-14.61511** | **3.49963** | **-9.99169** | **-14.60426** | **3.50744** | **-9.9919** | **-14.56444** | **3.48035** |
| **O** | **-13.5611** | **-15.14984** | **8.01898** | **-13.3447** | **-15.25044** | **8.16002** | **-13.24745** | **-15.23247** | **8.19842** |
| **C** | **-13.0731** | **-14.53622** | **7.07618** | **-12.89822** | **-14.61368** | **7.21199** | **-12.81501** | **-14.58833** | **7.24887** |
| **C** | **-11.56856** | **-14.18076** | **7.06937** | **-11.39572** | **-14.25516** | **7.14916** | **-11.31032** | **-14.24573** | **7.15684** |
| **N** | **-10.86199** | **-14.63798** | **5.85418** | **-10.7412** | **-14.68306** | **5.8948** | **-10.68875** | **-14.66503** | **5.88295** |
| **O** | **-16.37274** | **-15.86537** | **4.10363** | **-16.32094** | **-15.87801** | **4.35231** | **-16.31501** | **-15.77695** | **4.45108** |
| **C** | **-15.29068** | **-15.62403** | **4.6183** | **-15.21809** | **-15.64727** | **4.82613** | **-15.19932** | **-15.56474** | **4.90295** |
| **C** | **-15.14617** | **-14.57003** | **5.72758** | **-15.0272** | **-14.61986** | **5.95316** | **-14.97152** | **-14.55419** | **6.03833** |
| **N** | **-13.78094** | **-14.14438** | **5.97542** | **-13.65305** | **-14.19734** | **6.15222** | **-13.5885** | **-14.14971** | **6.2118** |
| **N** | **-14.11839** | **-16.25097** | **4.26204** | **-14.06167** | **-16.26368** | **4.40545** | **-14.05971** | **-16.18866** | **4.44875** |
| **S** | **-11.25476** | **-17.24231** | **3.62384** | **-11.22727** | **-17.23544** | **3.62146** | **-11.25491** | **-17.18291** | **3.58916** |
| **C** | **-12.80496** | **-17.69576** | **2.85948** | **-12.80868** | **-17.67227** | **2.91344** | **-12.85678** | **-17.59234** | **2.91147** |
| **C** | **-13.99047** | **-17.19352** | **3.25697** | **-13.97629** | **-17.18143** | **3.37327** | **-14.00799** | **-17.09407** | **3.40353** |
| **C** | **-7.97098** | **-12.09252** | **-0.32539** | **-8.11417** | **-11.99085** | **-0.33958** | **-8.17201** | **-11.92281** | **-0.37502** |
| **O** | **-6.82622** | **-11.76805** | **0.44771** | **-6.93823** | **-11.68397** | **0.39285** | **-6.97709** | **-11.6371** | **0.33491** |
| **N** | **-9.71332** | **-11.1473** | **1.28112** | **-9.78773** | **-11.08472** | **1.35986** | **-9.79816** | **-11.02061** | **1.37189** |
| **C** | **-11.31062** | **-12.69313** | **7.40392** | **-11.12497** | **-12.77513** | **7.5063** | **-11.01499** | **-12.77342** | **7.52616** |
| **C** | **-9.80812** | **-12.43415** | **7.37655** | **-9.62498** | **-12.51351** | **7.4227** | **-9.5143** | **-12.52747** | **7.41292** |
| **O** | **-9.03602** | **-13.14526** | **8.03187** | **-8.82603** | **-13.23924** | **8.02786** | **-8.71069** | **-13.26996** | **7.99094** |
| **N** | **-9.3736** | **-11.43444** | **6.57897** | **-9.22436** | **-11.49402** | **6.63239** | **-9.11939** | **-11.50228** | **6.62713** |
| **C** | **-8.27028** | **-17.21146** | **7.91088** | **-8.06487** | **-17.30024** | **7.77875** | **-8.00188** | **-17.33522** | **7.67267** |
| **O** | **-9.32618** | **-16.92687** | **7.3743** | **-9.14212** | **-17.00478** | **7.29284** | **-9.08604** | **-17.0213** | **7.21448** |
| **O** | **-8.12564** | **-18.32051** | **8.66202** | **-7.8889** | **-18.42631** | **8.49712** | **-7.82333** | **-18.4724** | **8.37267** |
| **H** | **-6.88776** | **-7.6629** | **4.14648** | **-6.84699** | **-7.66358** | **4.18273** | **-6.75868** | **-7.66782** | **4.17353** |
| **H** | **-5.11323** | **-7.77294** | **4.23596** | **-5.07033** | **-7.77447** | **4.19944** | **-4.98337** | **-7.79671** | **4.14747** |
| **H** | **-5.91645** | **-7.25481** | **2.73091** | **-5.93231** | **-7.22274** | **2.73954** | **-5.87314** | **-7.21733** | **2.71521** |
| **H** | **-4.6599** | **-9.95197** | **4.35253** | **-4.6115** | **-9.95483** | **4.24642** | **-4.54689** | **-9.98224** | **4.15671** |
| **H** | **-5.61294** | **-11.85932** | **2.31197** | **-5.647** | **-11.81522** | **2.20257** | **-5.64857** | **-11.80502** | **2.11352** |
| **H** | **-6.60148** | **-13.88167** | **3.00106** | **-6.60708** | **-13.85309** | **2.883** | **-6.61492** | **-13.84125** | **2.78936** |
| **H** | **-8.27287** | **-13.60527** | **5.40132** | **-8.1757** | **-13.63698** | **5.35748** | **-8.12484** | **-13.64048** | **5.30114** |
| **H** | **-8.91346** | **-16.08847** | **4.63022** | **-8.15444** | **-15.62149** | **3.03197** | **-8.17699** | **-15.59529** | **2.95118** |
| **H** | **-8.15764** | **-15.64449** | **3.12628** | **-8.84599** | **-16.10229** | **4.55553** | **-8.8399** | **-16.08798** | **4.48367** |
| **H** | **-3.98497** | **-13.40881** | **3.44416** | **-3.97211** | **-13.38834** | **3.22858** | **-3.96716** | **-13.40864** | **3.07989** |
| **H** | **-3.20051** | **-11.90841** | **2.91281** | **-3.21143** | **-11.87461** | **2.70042** | **-3.20295** | **-11.89602** | **2.55366** |
| **H** | **-3.60804** | **-12.15366** | **4.63008** | **-3.54645** | **-12.16093** | **4.42698** | **-3.50082** | **-12.20152** | **4.28374** |
| **H** | **-7.89205** | **-14.55458** | **7.3049** | **-7.71239** | **-14.62866** | **7.22127** | **-7.63061** | **-14.66074** | **7.14201** |
| **H** | **-6.26643** | **-17.03809** | **7.24537** | **-6.09067** | **-17.10849** | **7.03465** | **-6.04265** | **-17.15631** | **6.88716** |
| **H** | **-6.15228** | **-17.06062** | **9.6525** | **-5.87743** | **-17.18817** | **9.43436** | **-5.77729** | **-17.27011** | **9.28034** |
| **H** | **-4.48894** | **-15.24093** | **9.95584** | **-4.20107** | **-15.37561** | **9.71038** | **-4.07312** | **-15.48158** | **9.54176** |
| **H** | **-4.30746** | **-15.98278** | **8.35589** | **-4.08652** | **-16.08159** | **8.08816** | **-4.00348** | **-16.16756** | **7.90844** |
| **H** | **-5.10377** | **-14.40901** | **8.51796** | **-4.87368** | **-14.51168** | **8.31794** | **-4.76591** | **-14.59148** | **8.17596** |
| **H** | **-6.77459** | **-15.11587** | **11.06962** | **-6.43864** | **-15.27739** | **10.921** | **-6.28155** | **-15.37189** | **10.80329** |
| **H** | **-8.20517** | **-15.79355** | **10.28935** | **-7.90109** | **-15.93554** | **10.18423** | **-7.76799** | **-16.00353** | **10.09184** |
| **H** | **-7.49438** | **-14.29295** | **9.67822** | **-7.21358** | **-14.42136** | **9.58009** | **-7.07628** | **-14.48987** | **9.49114** |
| **H** | **-10.37171** | **-12.66158** | **2.71923** | **-10.38491** | **-12.63325** | **2.78908** | **-10.37987** | **-12.58063** | **2.79523** |
| **H** | **-10.04137** | **-12.57681** | **-0.1867** | **-10.17721** | **-12.47979** | **-0.126** | **-10.23487** | **-12.39312** | **-0.12184** |
| **H** | **-11.80241** | **-15.20548** | **2.42133** | **-11.82638** | **-15.17131** | **2.49252** | **-11.85452** | **-15.09822** | **2.49902** |
| **H** | **-10.29455** | **-15.92526** | **1.80922** | **-10.34557** | **-15.87408** | **1.79971** | **-10.3973** | **-15.80883** | **1.76546** |
| **H** | **-11.14124** | **-14.77714** | **7.87477** | **-10.93389** | **-14.86897** | **7.92173** | **-10.83836** | **-14.8743** | **7.91121** |
| **H** | **-10.28745** | **-15.46275** | **5.9982** | **-10.16014** | **-15.50979** | **5.99519** | **-10.11479** | **-15.49918** | **5.96025** |
| **H** | **-15.54556** | **-14.99133** | **6.65478** | **-15.38577** | **-15.06409** | **6.88633** | **-15.31422** | **-15.00645** | **6.9736** |
| **H** | **-15.77645** | **-13.72264** | **5.44364** | **-15.67009** | **-13.7673** | **5.71702** | **-15.6097** | **-13.6915** | **5.82755** |
| **H** | **-13.29528** | **-13.57943** | **5.28009** | **-13.19807** | **-13.61556** | **5.45004** | **-13.14255** | **-13.56426** | **5.50688** |
| **H** | **-13.27102** | **-16.00404** | **4.76123** | **-13.19398** | **-16.02765** | **4.87406** | **-13.17923** | **-15.96843** | **4.90092** |
| **H** | **-12.77126** | **-18.43799** | **2.07016** | **-12.80863** | **-18.39507** | **2.10557** | **-12.88332** | **-18.30471** | **2.09483** |
| **H** | **-14.92605** | **-17.48732** | **2.79194** | **-14.93074** | **-17.46513** | **2.94153** | **-14.97508** | **-17.36129** | **2.98976** |
| **H** | **-7.73339** | **-13.01696** | **-0.85581** | **-7.89919** | **-12.90248** | **-0.90089** | **-7.97902** | **-12.82962** | **-0.95191** |
| **H** | **-8.17805** | **-11.30539** | **-1.06675** | **-8.35152** | **-11.18667** | **-1.05304** | **-8.41644** | **-11.1075** | **-1.0733** |
| **H** | **-6.95355** | **-10.91843** | **0.91006** | **-7.04555** | **-10.84489** | **0.87892** | **-7.06507** | **-10.80341** | **0.83397** |
| **H** | **-8.95228** | **-10.67934** | **1.77179** | **-9.00658** | **-10.62706** | **1.82811** | **-9.00215** | **-10.57686** | **1.82836** |
| **H** | **-10.14893** | **-10.46313** | **0.67038** | **-10.24943** | **-10.38733** | **0.78425** | **-10.26503** | **-10.31142** | **0.81518** |
| **H** | **-11.84679** | **-12.02895** | **6.72466** | **-11.68903** | **-12.09661** | **6.86482** | **-11.58514** | **-12.0806** | **6.90569** |
| **H** | **-11.67459** | **-12.5179** | **8.42186** | **-11.44671** | **-12.62332** | **8.54208** | **-11.31229** | **-12.6311** | **8.57056** |
| **H** | **-8.37549** | **-11.36975** | **6.37686** | **-8.2356** | **-11.42354** | **6.39017** | **-8.13541** | **-11.43971** | **6.3642** |
| **H** | **-10.01261** | **-10.97949** | **5.94357** | **-9.88949** | **-11.02467** | **6.03543** | **-9.79192** | **-11.01754** | **6.05114** |
| **H** | **-8.97971** | **-18.78693** | **8.66407** | **-8.74191** | **-18.89393** | **8.52334** | **-8.68096** | **-18.93058** | **8.41179** |

# Table S18. Lowest-energy Conformers Optimized at the M062X/6-311+G (d, p) Level of **2**b with Relative Energies < 3.0 kcal/mol

| **2b_C1** | G 2773.5874 Ha  Boltzman pop. 55.52% | **2b_C2** | G 2773.5841 Ha  Boltzman pop. 1.62% | **2b_C3** | G 2773.5860 Ha  Boltzman pop. 42.86% |
| --- | --- | --- | --- | --- | --- |
| 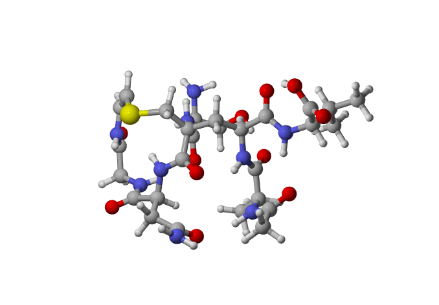 | | 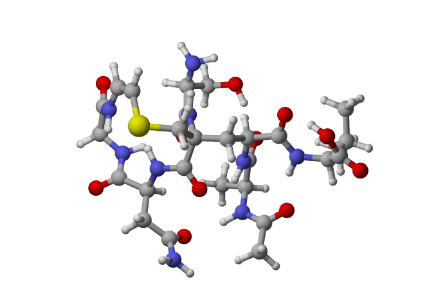 | | 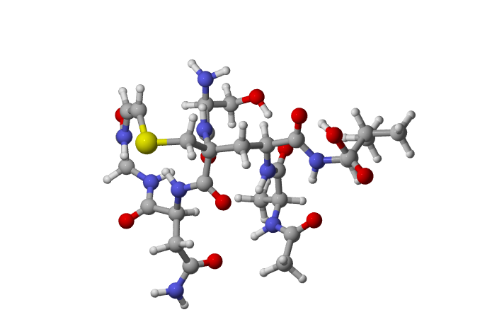 | |

# Table S19. Atomic Coordinates for the Lowest-energy Conformers of **2b** (**2b_C1**–**2b_C3**)

| **Atoms** | **2b_C1** | | | **2b_C2** | | | **2b_C3** | | |
| --- | --- | --- | --- | --- | --- | --- | --- | --- | --- |
|  | **x** | **y** | **z** | **x** | **y** | **z** | **x** | **y** | **z** |
| **C** | **2.38497** | **1.6961** | **5.34935** | **2.33601** | **1.6686** | **5.35619** | **1.99912** | **1.79029** | **5.30183** |
| **C** | **3.03047** | **2.54953** | **4.27757** | **2.99597** | **2.54221** | **4.30947** | **2.76607** | **2.62839** | **4.30163** |
| **N** | **2.60958** | **2.349** | **2.99995** | **2.59887** | **2.35782** | **3.02188** | **2.44576** | **2.45664** | **2.98926** |
| **C** | **3.25945** | **3.01196** | **1.87269** | **3.26258** | **3.03992** | **1.91421** | **3.21539** | **3.09258** | **1.9231** |
| **C** | **2.99352** | **4.52608** | **1.79464** | **2.9859** | **4.55291** | **1.84906** | **3.00219** | **4.6144** | **1.80595** |
| **N** | **2.03897** | **5.01974** | **2.61225** | **2.02258** | **5.03075** | **2.66631** | **2.06533** | **5.15921** | **2.60764** |
| **C** | **1.74821** | **6.43301** | **2.75034** | **1.71355** | **6.43887** | **2.81584** | **1.82018** | **6.58239** | **2.72649** |
| **C** | **0.27666** | **6.7036** | **3.13142** | **0.2339** | **6.68659** | **3.18078** | **0.35795** | **6.91295** | **3.1152** |
| **O** | **3.89529** | **3.39255** | **4.55417** | **3.84996** | **3.38628** | **4.61487** | **3.6354** | **3.43701** | **4.65681** |
| **C** | **2.89402** | **2.32054** | **0.55444** | **2.92631** | **2.36122** | **0.58167** | **2.95654** | **2.39818** | **0.5816** |
| **O** | **3.67202** | **5.22381** | **1.02937** | **3.66577** | **5.26328** | **1.09716** | **3.7107** | **5.26932** | **1.02813** |
| **C** | **2.64072** | **7.14187** | **3.79478** | **2.58492** | **7.15245** | **3.87798** | **2.73714** | **7.26883** | **3.76382** |
| **N** | **3.46846** | **6.38599** | **4.53093** | **3.43312** | **6.40054** | **4.59185** | **3.49256** | **6.48346** | **4.54622** |
| **O** | **2.52392** | **8.37711** | **3.91883** | **2.41989** | **8.38009** | **4.02315** | **2.69976** | **8.51243** | **3.84523** |
| **C** | **4.47406** | **6.8873** | **5.49064** | **4.45103** | **6.80945** | **5.5873** | **4.50284** | **6.94705** | **5.51953** |
| **C** | **5.56739** | **7.7703** | **4.82122** | **5.78234** | **7.33685** | **4.96537** | **5.67142** | **7.73287** | **4.8558** |
| **C** | **6.57717** | **8.26839** | **5.86787** | **6.38736** | **6.2945** | **4.01229** | **6.68655** | **8.19164** | **5.91521** |
| **C** | **6.27918** | **6.99229** | **3.70356** | **5.6791** | **8.7133** | **4.29119** | **6.35614** | **6.87595** | **3.77966** |
| **N** | **-0.46846** | **6.6107** | **0.76418** | **-0.47911** | **6.61857** | **0.80296** | **-0.42878** | **6.68594** | **0.77375** |
| **C** | **0.17421** | **5.95172** | **-0.23019** | **0.1829** | **5.97884** | **-0.1911** | **0.16343** | **5.94078** | **-0.18598** |
| **O** | **0.38832** | **4.73099** | **-0.27066** | **0.41141** | **4.76106** | **-0.2425** | **0.33939** | **4.71269** | **-0.14295** |
| **C** | **0.60546** | **6.85495** | **-1.40794** | **0.61797** | **6.90076** | **-1.35279** | **0.59159** | **6.74385** | **-1.43464** |
| **O** | **-0.54706** | **4.06438** | **3.26743** | **-0.55929** | **4.03476** | **3.26502** | **-0.43963** | **4.30033** | **3.39355** |
| **C** | **-2.1239** | **6.98572** | **2.53092** | **-2.16146** | **6.95176** | **2.55242** | **-2.05288** | **7.21248** | **2.52491** |
| **C** | **-1.02871** | **4.67478** | **2.30982** | **-1.03907** | **4.65547** | **2.31302** | **-0.99728** | **4.86686** | **2.45124** |
| **C** | **-0.81708** | **6.21441** | **2.1356** | **-0.84132** | **6.19963** | **2.16393** | **-0.76778** | **6.38916** | **2.17242** |
| **O** | **-3.71792** | **2.02179** | **-0.31444** | **-3.67031** | **2.02066** | **-0.3879** | **-3.661** | **1.98155** | **0.05974** |
| **C** | **-2.62163** | **2.56699** | **-0.258** | **-2.57929** | **2.57336** | **-0.30637** | **-2.58751** | **2.57603** | **0.05869** |
| **C** | **-1.78935** | **2.65643** | **1.04305** | **-1.76837** | **2.64954** | **1.00901** | **-1.73978** | **2.78686** | **1.33786** |
| **N** | **-1.81918** | **4.08918** | **1.38896** | **-1.81604** | **4.07628** | **1.37678** | **-1.86385** | **4.22488** | **1.63687** |
| **O** | **-2.69695** | **5.85586** | **-3.49722** | **-2.62625** | **5.90949** | **-3.49519** | **-2.70298** | **5.64013** | **-3.40573** |
| **C** | **-2.99657** | **5.18155** | **-2.52275** | **-2.94013** | **5.21729** | **-2.53788** | **-3.01582** | **5.04851** | **-2.38302** |
| **C** | **-2.79883** | **3.65539** | **-2.48234** | **-2.73028** | **3.69236** | **-2.51727** | **-2.84234** | **3.52691** | **-2.22296** |
| **N** | **-2.05524** | **3.24957** | **-1.29809** | **-2.00214** | **3.27543** | **-1.32718** | **-2.06874** | **3.21542** | **-1.02927** |
| **N** | **-3.47891** | **5.73649** | **-1.35098** | **-3.45052** | **5.74965** | **-1.36753** | **-3.48715** | **5.70185** | **-1.25814** |
| **S** | **-3.70994** | **6.68949** | **1.62358** | **-3.73167** | **6.65413** | **1.6181** | **-3.64992** | **6.88376** | **1.64315** |
| **C** | **-3.54734** | **7.65521** | **0.13343** | **-3.55741** | **7.64438** | **0.14546** | **-3.47886** | **7.72793** | **0.08189** |
| **C** | **-3.49503** | **7.10691** | **-1.09452** | **-3.48227** | **7.1158** | **-1.0899** | **-3.46112** | **7.08789** | **-1.10198** |
| **C** | **2.08631** | **6.58371** | **-1.75993** | **2.10563** | **6.65012** | **-1.69035** | **2.06135** | **6.42012** | **-1.7922** |
| **O** | **2.94281** | **7.19794** | **-0.81107** | **2.94398** | **7.2598** | **-0.72242** | **2.94637** | **7.10465** | **-0.92168** |
| **N** | **0.35433** | **8.2724** | **-1.11679** | **0.3476** | **8.31169** | **-1.0474** | **0.37368** | **8.184** | **-1.24621** |
| **C** | **-2.32729** | **1.73495** | **2.13822** | **-2.31576** | **1.70619** | **2.08071** | **-2.17143** | **1.87076** | **2.48865** |
| **C** | **-1.23189** | **1.18769** | **3.04863** | **-1.23048** | **1.15109** | **2.99837** | **-1.34678** | **0.58693** | **2.49587** |
| **O** | **-0.09487** | **0.93536** | **2.64251** | **-0.08437** | **0.9171** | **2.60721** | **-0.11269** | **0.60401** | **2.45363** |
| **N** | **-1.62394** | **0.90066** | **4.31615** | **-1.6408** | **0.83674** | **4.25359** | **-2.04424** | **-0.57253** | **2.59343** |
| **C** | **3.83562** | **7.56713** | **6.72442** | **3.98176** | **7.72188** | **6.74481** | **3.88104** | **7.71008** | **6.71266** |
| **O** | **4.10156** | **7.20144** | **7.84954** | **4.48166** | **7.57284** | **7.84165** | **4.09821** | **7.36977** | **7.85591** |
| **O** | **3.01738** | **8.60344** | **6.49753** | **3.08168** | **8.67643** | **6.5092** | **3.13685** | **8.78763** | **6.42951** |
| **H** | **3.17385** | **1.1542** | **5.87881** | **1.81734** | **2.3074** | **6.07721** | **2.71355** | **1.16717** | **5.84836** |
| **H** | **1.89302** | **2.34947** | **6.07591** | **1.63365** | **0.9524** | **4.92466** | **1.52283** | **2.45492** | **6.02796** |
| **H** | **1.66363** | **0.98406** | **4.94284** | **3.11881** | **1.13106** | **5.89886** | **1.24398** | **1.15457** | **4.83479** |
| **H** | **1.82118** | **1.72848** | **2.82499** | **1.8181** | **1.73416** | **2.82511** | **1.68246** | **1.82934** | **2.74403** |
| **H** | **4.34232** | **2.94804** | **2.02711** | **4.34311** | **2.98258** | **2.08684** | **4.27758** | **3.00039** | **2.1786** |
| **H** | **1.44716** | **4.36704** | **3.11621** | **1.42802** | **4.36682** | **3.15226** | **1.45593** | **4.53837** | **3.13049** |
| **H** | **1.97818** | **6.93054** | **1.80364** | **1.94914** | **6.9488** | **1.877** | **2.0624** | **7.06087** | **1.77324** |
| **H** | **0.0621** | **6.26684** | **4.11104** | **0.01204** | **6.23301** | **4.15114** | **0.15255** | **6.54484** | **4.12427** |
| **H** | **0.19369** | **7.78879** | **3.2328** | **0.13686** | **7.76892** | **3.29716** | **0.30372** | **8.00436** | **3.14979** |
| **H** | **3.4094** | **2.81643** | **-0.27019** | **3.45151** | **2.87149** | **-0.22782** | **3.56809** | **2.86609** | **-0.19206** |
| **H** | **3.20207** | **1.27151** | **0.58417** | **3.24306** | **1.31463** | **0.60442** | **3.22235** | **1.33953** | **0.65015** |
| **H** | **1.81824** | **2.36988** | **0.36613** | **1.85358** | **2.40364** | **0.37515** | **1.90677** | **2.48434** | **0.28737** |
| **H** | **3.49425** | **5.37963** | **4.36554** | **3.45519** | **5.4014** | **4.38823** | **3.44537** | **5.47217** | **4.41773** |
| **H** | **4.95298** | **5.9897** | **5.88749** | **4.70236** | **5.8741** | **6.091** | **4.90895** | **6.03319** | **5.95832** |
| **H** | **5.07269** | **8.64124** | **4.37585** | **6.45005** | **7.43309** | **5.83143** | **5.24937** | **8.62199** | **4.37304** |
| **H** | **7.35234** | **8.86744** | **5.37896** | **7.38772** | **6.61149** | **3.69905** | **7.51282** | **8.72237** | **5.43105** |
| **H** | **6.10968** | **8.89066** | **6.63651** | **6.47771** | **5.31313** | **4.49052** | **6.24375** | **8.86596** | **6.65389** |
| **H** | **7.06937** | **7.42912** | **6.37329** | **5.78101** | **6.17602** | **3.10784** | **7.10729** | **7.33596** | **6.4564** |
| **H** | **7.03507** | **7.62676** | **3.22886** | **6.6726** | **9.02989** | **3.95538** | **7.16806** | **7.44219** | **3.3112** |
| **H** | **5.58997** | **6.65835** | **2.92305** | **5.30089** | **9.48043** | **4.97372** | **5.66644** | **6.57065** | **2.98792** |
| **H** | **6.78961** | **6.10855** | **4.10627** | **5.02496** | **8.68865** | **3.41465** | **6.79143** | **5.96996** | **4.21964** |
| **H** | **-0.53703** | **7.60866** | **0.54268** | **-0.55623** | **7.61843** | **0.59306** | **-0.47534** | **7.66812** | **0.48568** |
| **H** | **-0.01309** | **6.54381** | **-2.25967** | **0.01293** | **6.59348** | **-2.21556** | **-0.04771** | **6.38169** | **-2.25001** |
| **H** | **-2.36522** | **6.75065** | **3.57146** | **-2.41508** | **6.69842** | **3.58572** | **-2.2959** | **7.05281** | **3.57924** |
| **H** | **-1.91645** | **8.05913** | **2.48186** | **-1.96419** | **8.02778** | **2.52259** | **-1.82554** | **8.27595** | **2.40236** |
| **H** | **-0.75155** | **2.36872** | **0.83092** | **-0.72485** | **2.37418** | **0.8092** | **-0.685** | **2.59012** | **1.11417** |
| **H** | **-2.10057** | **4.70481** | **0.64114** | **-2.10095** | **4.70119** | **0.63799** | **-2.24157** | **4.79901** | **0.89815** |
| **H** | **-2.26975** | **3.38166** | **-3.39742** | **-2.18407** | **3.43677** | **-3.4275** | **-2.34494** | **3.17031** | **-3.12749** |
| **H** | **-3.76278** | **3.13716** | **-2.46389** | **-3.68982** | **3.16576** | **-2.52197** | **-3.8116** | **3.02627** | **-2.133** |
| **H** | **-1.12311** | **3.64114** | **-1.15901** | **-1.07595** | **3.67316** | **-1.16709** | **-1.14943** | **3.64852** | **-0.92399** |
| **H** | **-3.75287** | **5.12043** | **-0.59554** | **-3.7333** | **5.11929** | **-0.62734** | **-3.76892** | **5.14933** | **-0.45834** |
| **H** | **-3.57262** | **8.73639** | **0.22894** | **-3.59404** | **8.72365** | **0.25736** | **-3.47219** | **8.81351** | **0.09511** |
| **H** | **-3.4282** | **7.72264** | **-1.98526** | **-3.40761** | **7.7461** | **-1.96975** | **-3.39047** | **7.63442** | **-2.03646** |
| **H** | **2.26083** | **5.50547** | **-1.82971** | **2.29268** | **5.5749** | **-1.77311** | **2.22075** | **5.3375** | **-1.76878** |
| **H** | **2.29163** | **7.03744** | **-2.73674** | **2.31789** | **7.11983** | **-2.65805** | **2.24909** | **6.78095** | **-2.81048** |
| **H** | **3.25455** | **6.5323** | **-0.16401** | **3.25442** | **6.58804** | **-0.08124** | **3.27399** | **6.49239** | **-0.23033** |
| **H** | **1.22662** | **8.7092** | **-0.81972** | **1.21121** | **8.75455** | **-0.7344** | **1.26262** | **8.62452** | **-1.01011** |
| **H** | **0.0138** | **8.76532** | **-1.93558** | **0.01154** | **8.81084** | **-1.86427** | **0.01558** | **8.61949** | **-2.08963** |
| **H** | **-3.11182** | **2.23848** | **2.70926** | **-3.11213** | **2.19482** | **2.64834** | **-1.94838** | **2.37827** | **3.43034** |
| **H** | **-2.80114** | **0.87216** | **1.65461** | **-2.77655** | **0.84841** | **1.57598** | **-3.24458** | **1.67445** | **2.43154** |
| **H** | **-0.94543** | **0.52483** | **4.96232** | **-0.97168** | **0.44905** | **4.90248** | **-1.53496** | **-1.44527** | **2.59032** |
| **H** | **-2.51308** | **1.20573** | **4.67989** | **-2.54186** | **1.11923** | **4.60598** | **-3.04859** | **-0.59448** | **2.50587** |
| **H** | **2.85838** | **8.72016** | **5.52221** | **2.82147** | **8.70428** | **5.54349** | **3.01075** | **8.88153** | **5.44693** |

# Table S20. Lowest-energy Conformers Optimized at the M062X/6-311+G (d, p) Level of **2**c with Relative Energies < 3.0 kcal/mol

| **2c_C1** | G 2773.5748 Ha  Boltzman pop. 33.29% | **2c_C2** | G 2773.5748 Ha  Boltzman pop. 33.37% | **2c_C3** | G 2773.5748 Ha  Boltzman pop. 33.34% |
| --- | --- | --- | --- | --- | --- |
| 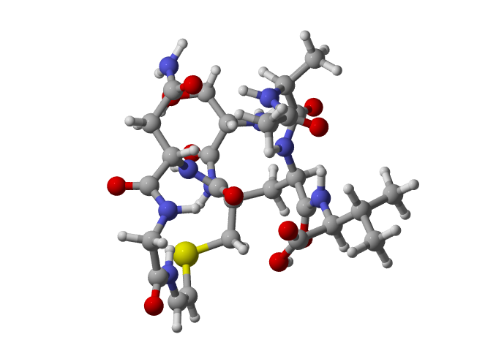 | | 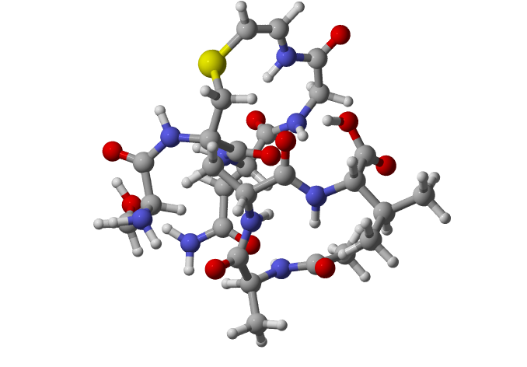 | | 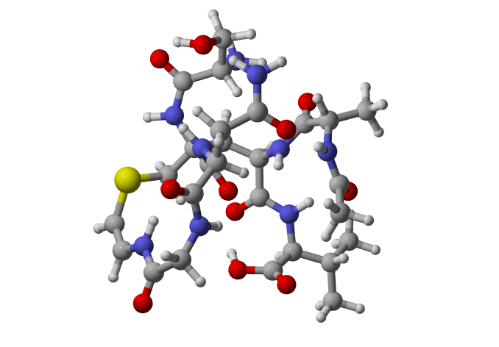 | |

# Table S21. Atomic Coordinates for the Lowest-energy Conformers of **2c** (**2c_C1**–**2c_C3**)

| **Atoms** | **2c_C1** | | | **2c_C2** | | | **2c_C3** | | |
| --- | --- | --- | --- | --- | --- | --- | --- | --- | --- |
|  | **x** | **y** | **z** | **x** | **y** | **z** | **x** | **y** | **z** |
| **C** | **-4.05555** | **7.9873** | **2.12711** | **-4.03705** | **7.94083** | **2.10506** | **-4.04387** | **7.93156** | **2.09257** |
| **C** | **-4.2355** | **7.68657** | **0.65421** | **-4.23734** | **7.64921** | **0.63296** | **-4.23765** | **7.64715** | **0.61819** |
| **N** | **-4.15129** | **8.74509** | **-0.19762** | **-4.14171** | **8.70931** | **-0.21572** | **-4.14669** | **8.71271** | **-0.22412** |
| **C** | **-4.13135** | **8.62365** | **-1.65838** | **-4.13852** | **8.59315** | **-1.67706** | **-4.13854** | **8.60497** | **-1.68608** |
| **C** | **-5.45147** | **8.11005** | **-2.27813** | **-5.4739** | **8.10609** | **-2.28545** | **-5.46897** | **8.11271** | **-2.3011** |
| **N** | **-6.54002** | **8.11044** | **-1.46705** | **-6.55417** | **8.12448** | **-1.46359** | **-6.55164** | **8.11909** | **-1.4822** |
| **C** | **-7.8173** | **7.50075** | **-1.819** | **-7.84668** | **7.54119** | **-1.80448** | **-7.83917** | **7.52897** | **-1.83007** |
| **C** | **-8.88838** | **8.46993** | **-2.36217** | **-8.90395** | **8.53339** | **-2.33302** | **-8.90178** | **8.51691** | **-2.35585** |
| **O** | **-4.47237** | **6.53883** | **0.24953** | **-4.49991** | **6.50775** | **0.22638** | **-4.49122** | **6.50629** | **0.20428** |
| **C** | **-2.93162** | **7.81761** | **-2.18072** | **-2.95886** | **7.76763** | **-2.21442** | **-2.95202** | **7.79015** | **-2.2247** |
| **O** | **-5.49635** | **7.78734** | **-3.46694** | **-5.53651** | **7.78806** | **-3.4747** | **-5.52613** | **7.80119** | **-3.49234** |
| **C** | **-8.32115** | **6.58274** | **-0.68852** | **-8.3568** | **6.62934** | **-0.67182** | **-8.34617** | **6.60707** | **-0.70415** |
| **N** | **-7.38591** | **5.93688** | **0.036** | **-7.42692** | **5.96304** | **0.04098** | **-7.41375** | **5.94302** | **0.00743** |
| **O** | **-9.53947** | **6.4117** | **-0.50546** | **-9.57628** | **6.48166** | **-0.47655** | **-9.56515** | **6.44986** | **-0.51328** |
| **C** | **-7.75428** | **5.02758** | **1.13266** | **-7.80138** | **5.05825** | **1.13929** | **-7.78517** | **5.02925** | **1.0993** |
| **C** | **-6.638** | **4.01401** | **1.44417** | **-6.70172** | **4.02251** | **1.43666** | **-6.67936** | **3.99927** | **1.39392** |
| **C** | **-7.09711** | **3.04304** | **2.5447** | **-7.16781** | **3.05796** | **2.5399** | **-7.14221** | **3.0251** | **2.49005** |
| **C** | **-6.22521** | **3.23762** | **0.18285** | **-6.31736** | **3.24137** | **0.16932** | **-6.28589** | **3.2282** | **0.12321** |
| **N** | **-9.60811** | **10.87909** | **-2.44382** | **-9.57631** | **10.95664** | **-2.39808** | **-9.59069** | **10.93576** | **-2.40925** |
| **C** | **-8.83943** | **11.50658** | **-3.39344** | **-8.80449** | **11.57262** | **-3.35268** | **-8.82069** | **11.56212** | **-3.35854** |
| **O** | **-9.32332** | **12.44703** | **-4.03151** | **-9.27558** | **12.52536** | **-3.98205** | **-9.29686** | **12.51462** | **-3.98442** |
| **C** | **-7.38201** | **11.0886** | **-3.66011** | **-7.35846** | **11.12659** | **-3.63548** | **-7.3707** | **11.12792** | **-3.63953** |
| **O** | **-8.20874** | **9.16933** | **0.48917** | **-8.18176** | **9.2074** | **0.5138** | **-8.192** | **9.17993** | **0.49671** |
| **C** | **-10.80511** | **9.35527** | **-0.97615** | **-10.789** | **9.45098** | **-0.92456** | **-10.79683** | **9.41354** | **-0.94735** |
| **C** | **-8.49883** | **10.05804** | **-0.32329** | **-8.46274** | **10.10509** | **-0.29198** | **-8.47686** | **10.08015** | **-0.30487** |
| **C** | **-9.4022** | **9.71625** | **-1.54555** | **-9.3848** | **9.7863** | **-1.50641** | **-9.39348** | **9.76181** | **-1.52358** |
| **O** | **-8.2568** | **13.16536** | **2.60027** | **-8.13467** | **13.19468** | **2.64113** | **-8.17655** | **13.15535** | **2.64668** |
| **C** | **-8.00566** | **12.01937** | **2.24185** | **-7.90821** | **12.04556** | **2.27629** | **-7.94191** | **12.00991** | **2.27555** |
| **C** | **-7.18964** | **11.71975** | **0.95487** | **-7.10991** | **11.73579** | **0.98062** | **-7.13828** | **11.71289** | **0.98017** |
| **N** | **-8.06048** | **11.32697** | **-0.16438** | **-7.99835** | **11.3646** | **-0.13216** | **-8.02141** | **11.34185** | **-0.13688** |
| **O** | **-11.28312** | **10.15333** | **4.99847** | **-11.19469** | **10.23218** | **5.05646** | **-11.22361** | **10.15897** | **5.03676** |
| **C** | **-10.62174** | **10.48068** | **4.02631** | **-10.53617** | **10.55107** | **4.07955** | **-10.56471** | **10.48741** | **4.06328** |
| **C** | **-9.16164** | **10.93878** | **4.15708** | **-9.06619** | **10.97971** | **4.19846** | **-9.0979** | **10.92511** | **4.18834** |
| **N** | **-8.39477** | **10.90199** | **2.92053** | **-8.31171** | **10.9331** | **2.95463** | **-8.34004** | **10.89086** | **2.94618** |
| **N** | **-11.09695** | **10.48721** | **2.73665** | **-11.02335** | **10.57306** | **2.7945** | **-11.04874** | **10.5131** | **2.77713** |
| **S** | **-11.68588** | **10.81122** | **-0.21778** | **-11.63394** | **10.92125** | **-0.15321** | **-11.65386** | **10.87364** | **-0.17001** |
| **C** | **-12.71621** | **10.05958** | **1.02711** | **-12.66712** | **10.18536** | **1.09875** | **-12.68541** | **10.12378** | **1.07498** |
| **C** | **-12.33256** | **10.0385** | **2.32007** | **-12.27158** | **10.15098** | **2.38785** | **-12.29306** | **10.08492** | **2.36492** |
| **C** | **-6.57323** | **12.34508** | **-4.0427** | **-6.52832** | **12.36821** | **-4.02084** | **-6.5483** | **12.37758** | **-4.01542** |
| **O** | **-6.77711** | **13.44549** | **-3.14749** | **-6.70173** | **13.46889** | **-3.11958** | **-6.73224** | **13.47188** | **-3.10849** |
| **N** | **-7.37446** | **10.06647** | **-4.70405** | **-7.38173** | **10.10904** | **-4.68367** | **-7.38367** | **10.11614** | **-4.69344** |
| **C** | **-6.31522** | **12.91338** | **0.55623** | **-6.21648** | **12.9142** | **0.57888** | **-6.25195** | **12.89957** | **0.58723** |
| **C** | **-5.00375** | **12.47902** | **-0.09048** | **-4.91958** | **12.45772** | **-0.08185** | **-4.95044** | **12.45566** | **-0.07299** |
| **O** | **-4.30117** | **11.59673** | **0.42003** | **-4.2293** | **11.56001** | **0.41849** | **-4.25547** | **11.55948** | **0.42355** |
| **N** | **-4.62513** | **13.15936** | **-1.1956** | **-4.53826** | **13.13541** | **-1.18768** | **-4.57075** | **13.14279** | **-1.17358** |
| **C** | **-8.1404** | **5.91691** | **2.34161** | **-8.15731** | **5.95184** | **2.35435** | **-8.15082** | **5.91317** | **2.31853** |
| **O** | **-7.41816** | **6.131** | **3.29071** | **-7.42098** | **6.14953** | **3.29615** | **-7.41857** | **6.11053** | **3.26358** |
| **O** | **-9.35502** | **6.48901** | **2.2461** | **-9.36157** | **6.5476** | **2.27321** | **-9.35904** | **6.50085** | **2.2374** |
| **H** | **-4.89986** | **7.56716** | **2.68166** | **-3.13953** | **7.41161** | **2.441** | **-3.14515** | **7.40458** | **2.42878** |
| **H** | **-3.15162** | **7.47637** | **2.47402** | **-3.9224** | **9.00563** | **2.3219** | **-3.93501** | **8.99565** | **2.31576** |
| **H** | **-3.9632** | **9.05485** | **2.34108** | **-4.8836** | **7.53464** | **2.66657** | **-4.89049** | **7.51828** | **2.6488** |
| **H** | **-4.00456** | **9.67811** | **0.18165** | **-3.9735** | **9.63788** | **0.16562** | **-3.9857** | **9.64018** | **0.16292** |
| **H** | **-4.03264** | **9.65314** | **-2.02381** | **-4.02487** | **9.62214** | **-2.03961** | **-4.03046** | **9.63672** | **-2.04241** |
| **H** | **-6.42816** | **8.43135** | **-0.51543** | **-6.4267** | **8.44019** | **-0.51221** | **-6.42889** | **8.43002** | **-0.52862** |
| **H** | **-7.59859** | **6.84028** | **-2.66901** | **-7.64978** | **6.87967** | **-2.65899** | **-7.63536** | **6.87378** | **-2.68782** |
| **H** | **-8.51112** | **8.83149** | **-3.31874** | **-8.52918** | **8.89141** | **-3.29192** | **-8.52697** | **8.8828** | **-3.31175** |
| **H** | **-9.76264** | **7.85598** | **-2.5928** | **-9.7924** | **7.93769** | **-2.55718** | **-9.78552** | **7.9164** | **-2.58575** |
| **H** | **-2.95374** | **7.79048** | **-3.27154** | **-2.99242** | **7.74501** | **-3.30506** | **-2.98222** | **7.77369** | **-3.31554** |
| **H** | **-2.00278** | **8.2912** | **-1.85019** | **-2.01832** | **8.22312** | **-1.89162** | **-2.01539** | **8.24977** | **-1.89643** |
| **H** | **-2.96271** | **6.79403** | **-1.80305** | **-3.00465** | **6.74334** | **-1.84018** | **-2.99232** | **6.76342** | **-1.85657** |
| **H** | **-6.39407** | **6.15005** | **-0.07741** | **-6.43236** | **6.15726** | **-0.08221** | **-6.42018** | **6.14463** | **-0.11187** |
| **H** | **-8.65005** | **4.48416** | **0.80782** | **-8.7109** | **4.53311** | **0.8227** | **-8.69015** | **4.49977** | **0.77695** |
| **H** | **-5.77123** | **4.57448** | **1.81221** | **-5.82043** | **4.56526** | **1.79668** | **-5.80286** | **4.54584** | **1.75981** |
| **H** | **-6.29905** | **2.32797** | **2.76905** | **-6.38146** | **2.32697** | **2.75404** | **-6.35146** | **2.29834** | **2.70243** |
| **H** | **-7.3468** | **3.56522** | **3.47125** | **-7.39731** | **3.58264** | **3.47026** | **-7.37827** | **3.5427** | **3.42273** |
| **H** | **-7.97332** | **2.46667** | **2.22045** | **-8.05852** | **2.49954** | **2.22387** | **-8.028** | **2.46236** | **2.16793** |
| **H** | **-5.45229** | **2.50449** | **0.43523** | **-5.55595** | **2.49303** | **0.41183** | **-5.52044** | **2.48335** | **0.36373** |
| **H** | **-5.81727** | **3.88857** | **-0.59472** | **-5.90537** | **3.88625** | **-0.61116** | **-5.87557** | **3.88037** | **-0.65208** |
| **H** | **-7.07697** | **2.69054** | **-0.24093** | **-7.1839** | **2.71165** | **-0.24648** | **-7.14765** | **2.69541** | **-0.29855** |
| **H** | **-10.5488** | **11.26404** | **-2.45304** | **-10.50912** | **11.36041** | **-2.39643** | **-10.52637** | **11.33283** | **-2.40815** |
| **H** | **-6.95807** | **10.64667** | **-2.75437** | **-6.93467** | **10.67226** | **-2.73584** | **-6.94633** | **10.67156** | **-2.7412** |
| **H** | **-11.41784** | **8.96719** | **-1.79327** | **-11.41702** | **9.07769** | **-1.73696** | **-11.42016** | **9.0405** | **-1.76346** |
| **H** | **-10.6858** | **8.57386** | **-0.22803** | **-10.67805** | **8.66495** | **-0.18001** | **-10.68233** | **8.62412** | **-0.20695** |
| **H** | **-6.54106** | **10.86485** | **1.16204** | **-6.47598** | **10.86775** | **1.17835** | **-6.49891** | **10.84811** | **1.17464** |
| **H** | **-8.44847** | **12.04935** | **-0.75732** | **-8.37842** | **12.09691** | **-0.71802** | **-8.40467** | **12.07477** | **-0.71989** |
| **H** | **-8.69622** | **10.29872** | **4.91122** | **-8.60646** | **10.32742** | **4.94557** | **-8.6357** | **10.27143** | **4.93272** |
| **H** | **-9.15561** | **11.96768** | **4.52817** | **-9.03643** | **12.00672** | **4.57363** | **-9.07589** | **11.95002** | **4.56971** |
| **H** | **-8.17207** | **9.99903** | **2.51017** | **-8.11017** | **10.02766** | **2.53884** | **-8.13155** | **9.98925** | **2.52552** |
| **H** | **-10.4783** | **10.82059** | **2.00635** | **-10.40528** | **10.89788** | **2.05989** | **-10.43092** | **10.84589** | **2.04588** |
| **H** | **-13.6934** | **9.69567** | **0.73316** | **-13.6542** | **9.84241** | **0.8128** | **-13.66939** | **9.77579** | **0.78447** |
| **H** | **-12.97073** | **9.65283** | **3.10865** | **-12.90979** | **9.77469** | **3.18091** | **-12.93078** | **9.69995** | **3.15421** |
| **H** | **-5.50633** | **12.10525** | **-4.01655** | **-5.46622** | **12.10703** | **-4.00573** | **-5.48441** | **12.1239** | **-3.99859** |
| **H** | **-6.83391** | **12.64363** | **-5.0641** | **-6.79255** | **12.67599** | **-5.03859** | **-6.81171** | **12.68922** | **-5.03221** |
| **H** | **-7.64679** | **13.81765** | **-3.36311** | **-7.56592** | **13.85898** | **-3.32538** | **-7.59848** | **13.8571** | **-3.3148** |
| **H** | **-7.49862** | **10.45758** | **-5.63239** | **-7.50698** | **10.50657** | **-5.60913** | **-7.50914** | **10.5179** | **-5.61703** |
| **H** | **-6.55698** | **9.46238** | **-4.67097** | **-6.57633** | **9.48845** | **-4.66112** | **-6.57399** | **9.50112** | **-4.67198** |
| **H** | **-6.86604** | **13.61798** | **-0.07319** | **-6.75956** | **13.63201** | **-0.04231** | **-6.79839** | **13.61718** | **-0.03123** |
| **H** | **-6.04704** | **13.46477** | **1.46315** | **-5.92938** | **13.45633** | **1.4856** | **-5.9707** | **13.43855** | **1.49765** |
| **H** | **-3.73843** | **12.91334** | **-1.61432** | **-3.66048** | **12.87396** | **-1.61575** | **-3.69029** | **12.88961** | **-1.60112** |
| **H** | **-5.30354** | **13.66085** | **-1.76198** | **-5.21242** | **13.6513** | **-1.74619** | **-5.24698** | **13.65746** | **-1.73069** |
| **H** | **-9.71244** | **6.36044** | **1.33665** | **-9.73093** | **6.4286** | **1.36724** | **-9.72497** | **6.3845** | **1.3297** |

# Table S22. Lowest-energy Conformers Optimized at the M062X/6-311+G (d, p) Level of **2d** with Relative Energies < 3.0 kcal/mol

| **2d_C1** | G 2773.5940 Ha  Boltzman pop. 18.65% | **2d_C2** | G 2773.5934 Ha  Boltzman pop. 11.53% | **2d_C3** | G 2773.5924 Ha  Boltzman pop. 2.90% |
| --- | --- | --- | --- | --- | --- |
| 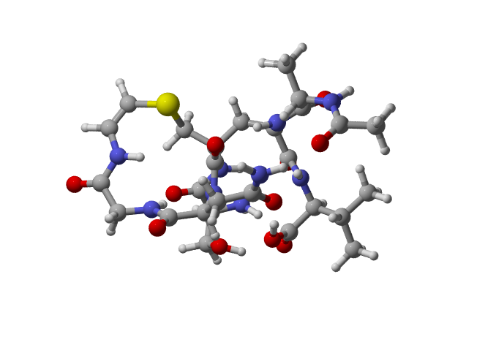 | | 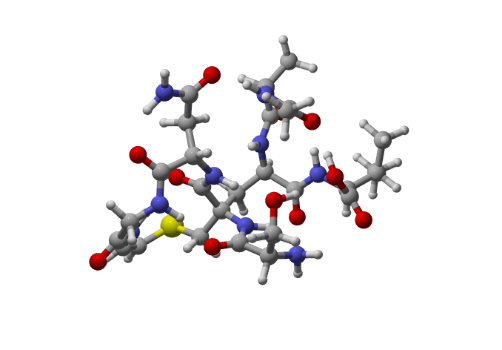 | | 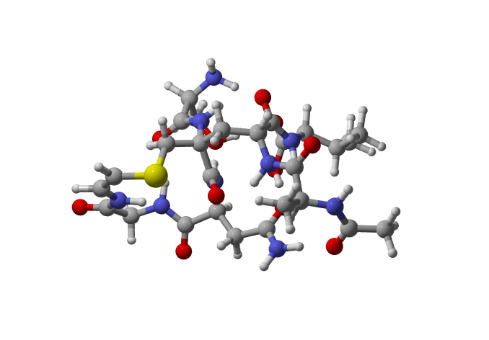 | |
| **1d_C4** | G 2773.5947 Ha  Boltzman pop. 62.06% | **1d_C5** | G 2773.5923 Ha  Boltzman pop. 4.85% |  |  |
| 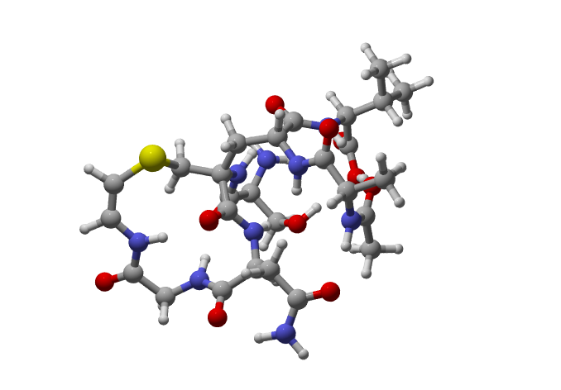 | | 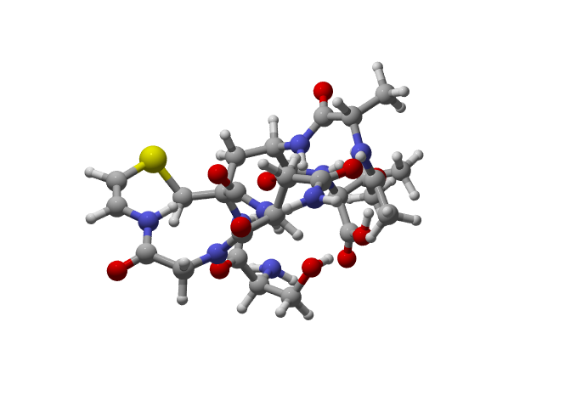 | |  | |

# Table S23. Atomic Coordinates for the Lowest-energy Conformers of **2d** (**2d_C1**–**2d_C5**)

| **Atoms** | **2d_C1** | | | **2d_C2** | | | **2d_C3** | | | **2d_C4** | | | **2d_C5** | | |
| --- | --- | --- | --- | --- | --- | --- | --- | --- | --- | --- | --- | --- | --- | --- | --- |
|  | **x** | **y** | **z** | **x** | **y** | **z** | **x** | **y** | **z** | **x** | **y** | **z** | **x** | **y** | **z** |
| **N** | **6.68853** | **7.99924** | **-1.35954** | **2.5726** | **5.65636** | **-4.35585** | **6.6968** | **7.97399** | **-1.39339** | **2.65743** | **5.689** | **-4.31361** | **2.6986** | **5.6447** | **-4.23489** |
| **C** | **5.82488** | **6.79495** | **-1.04378** | **2.78334** | **6.16824** | **-2.94867** | **5.82674** | **6.77942** | **-1.05939** | **2.84018** | **6.19032** | **-2.89844** | **2.8668** | **6.14946** | **-2.81946** |
| **C** | **5.25747** | **6.77253** | **0.18984** | **3.55172** | **5.42391** | **-2.13661** | **5.26169** | **6.77682** | **0.17593** | **3.56804** | **5.42304** | **-2.07043** | **3.60073** | **5.39507** | **-1.98525** |
| **C** | **4.32901** | **5.74379** | **0.64297** | **4.00948** | **5.83999** | **-0.80342** | **4.3372** | **5.75292** | **0.64751** | **4.00272** | **5.81345** | **-0.72131** | **4.01678** | **5.79922** | **-0.6347** |
| **N** | **2.9595** | **6.40318** | **0.87955** | **2.87787** | **6.20388** | **0.18436** | **2.96553** | **6.40917** | **0.87721** | **2.85599** | **6.16475** | **0.25426** | **2.85676** | **6.16019** | **0.32083** |
| **O** | **1.87627** | **5.65069** | **0.54747** | **1.66796** | **5.58686** | **0.00873** | **1.88642** | **5.65996** | **0.52114** | **1.64447** | **5.55988** | **0.04632** | **1.64615** | **5.56088** | **0.09489** |
| **C** | **0.52185** | **6.0732** | **0.89268** | **0.5272** | **5.90872** | **0.85663** | **0.52775** | **6.06553** | **0.86728** | **0.48446** | **5.89546** | **0.86501** | **0.48035** | **5.885** | **0.90708** |
| **C** | **-0.34262** | **4.90331** | **1.42862** | **-0.27977** | **4.69616** | **1.37687** | **-0.31009** | **4.88723** | **1.429** | **-0.34812** | **4.6964** | **1.37347** | **-0.34938** | **4.67502** | **1.39675** |
| **C** | **5.67969** | **5.8845** | **-1.87095** | **2.23869** | **7.23021** | **-2.56009** | **5.67319** | **5.85881** | **-1.87333** | **2.30723** | **7.26315** | **-2.522** | **2.31861** | **7.21484** | **-2.44539** |
| **C** | **4.82736** | **5.07385** | **1.93514** | **5.06052** | **6.9552** | **-0.87801** | **4.83954** | **5.10511** | **1.94921** | **5.06079** | **6.92405** | **-0.7555** | **5.07496** | **6.90987** | **-0.6664** |
| **C** | **2.8836** | **7.53678** | **1.35432** | **3.09373** | **6.97427** | **1.11387** | **2.88212** | **7.53612** | **1.36552** | **3.06013** | **6.91898** | **1.19948** | **3.0509** | **6.91261** | **1.26942** |
| **N** | **-0.19948** | **6.89782** | **-0.18932** | **-0.39198** | **7.02328** | **0.29358** | **-0.20952** | **6.8588** | **-0.2277** | **-0.3985** | **7.02642** | **0.27219** | **-0.4135** | **7.0072** | **0.31868** |
| **C** | **0.45304** | **7.11066** | **-1.35608** | **0.14355** | **7.85842** | **-0.62935** | **0.46618** | **7.13927** | **-1.36011** | **0.2016** | **7.84161** | **-0.62627** | **0.15802** | **7.83942** | **-0.58542** |
| **O** | **-1.32432** | **7.34131** | **0.05442** | **-1.54464** | **7.13186** | **0.72361** | **-1.37387** | **7.22667** | **-0.0183** | **-1.55943** | **7.17075** | **0.66328** | **-1.57766** | **7.12488** | **0.71328** |
| **C** | **-0.13956** | **7.88434** | **-2.4457** | **-0.65344** | **8.89732** | **-1.27401** | **-0.12474** | **7.91034** | **-2.45477** | **-0.4798** | **8.9448** | **-1.29645** | **-0.60517** | **8.89677** | **-1.24304** |
| **O** | **0.94338** | **8.65141** | **-3.24553** | **-0.02738** | **10.31981** | **-1.1999** | **0.96473** | **8.6825** | **-3.24037** | **0.43249** | **10.20098** | **-1.38112** | **0.06776** | **10.29675** | **-1.11973** |
| **C** | **0.33551** | **9.3405** | **-4.47762** | **0.12663** | **10.75615** | **0.26465** | **0.36889** | **9.36942** | **-4.47947** | **-0.23389** | **11.32866** | **-2.18471** | **1.36323** | **10.44323** | **-1.93259** |
| **C** | **1.6523** | **9.67212** | **-2.34122** | **1.29484** | **10.46261** | **-1.96944** | **1.65941** | **9.7062** | **-2.32805** | **0.80801** | **10.68051** | **0.02971** | **-0.92755** | **11.41773** | **-1.45759** |
| **C** | **-2.26955** | **4.47137** | **-0.06515** | **-1.82771** | **4.4403** | **-0.57088** | **-2.25659** | **4.45452** | **-0.02817** | **-1.86125** | **4.42688** | **-0.59463** | **-1.83164** | **4.43034** | **-0.60205** |
| **O** | **-3.25822** | **3.81899** | **-0.72543** | **-2.57353** | **3.85488** | **-1.53741** | **-3.26996** | **3.81308** | **-0.66716** | **-2.5879** | **3.83666** | **-1.5726** | **-2.54566** | **3.85171** | **-1.59633** |
| **C** | **-3.26929** | **2.59943** | **-0.95451** | **-2.6433** | **2.6304** | **-1.7206** | **-3.29902** | **2.58923** | **-0.86763** | **-2.64488** | **2.61222** | **-1.76229** | **-2.61169** | **2.62857** | **-1.78976** |
| **C** | **-4.37073** | **4.70916** | **-1.30816** | **-3.25058** | **4.81302** | **-2.52841** | **-4.39635** | **4.69857** | **-1.24811** | **-3.25854** | **4.79954** | **-2.56404** | **-3.18722** | **4.81898** | **-2.6021** |
| **N** | **1.08884** | **3.06069** | **-0.32701** | **0.53027** | **1.85689** | **0.33956** | **1.10413** | **3.06129** | **-0.35115** | **0.49232** | **1.85394** | **0.35488** | **0.48385** | **1.83409** | **0.37772** |
| **O** | **-1.46747** | **2.65945** | **1.37162** | **-2.03117** | **2.87234** | **1.28566** | **-1.42465** | **2.63616** | **1.38703** | **-2.08612** | **2.86208** | **1.25998** | **-2.10274** | **2.85702** | **1.24066** |
| **C** | **-0.05464** | **3.41649** | **-0.64927** | **0.00146** | **2.78072** | **-0.27902** | **-0.04667** | **3.41095** | **-0.654** | **-0.0287** | **2.77657** | **-0.27202** | **-0.01904** | **2.76308** | **-0.25459** |
| **C** | **-1.03359** | **3.87419** | **0.47699** | **-1.02492** | **3.71064** | **0.42221** | **-1.01059** | **3.85377** | **0.48962** | **-1.07014** | **3.70363** | **0.41071** | **-1.06501** | **3.6947** | **0.41473** |
| **N** | **0.31139** | **0.48036** | **-3.92048** | **1.35672** | **-0.35979** | **-2.49031** | **0.25599** | **0.50605** | **-3.95867** | **1.36728** | **-0.36145** | **-2.46293** | **1.39696** | **-0.37552** | **-2.42926** |
| **N** | **-0.31264** | **1.24527** | **-3.18887** | **0.59036** | **0.59223** | **-2.29409** | **-0.35188** | **1.26673** | **-3.20976** | **0.59549** | **0.58876** | **-2.27828** | **0.62823** | **0.57919** | **-2.25679** |
| **S** | **0.14462** | **2.73216** | **-3.05922** | **1.16123** | **2.03716** | **-2.29767** | **0.11777** | **2.74871** | **-3.06977** | **1.16323** | **2.0351** | **-2.27329** | **1.20489** | **2.02175** | **-2.23604** |
| **C** | **-0.48914** | **3.41636** | **-1.92825** | **0.32605** | **3.00884** | **-1.58656** | **-0.5023** | **3.42154** | **-1.92479** | **0.31652** | **3.00559** | **-1.57428** | **0.35057** | **2.99444** | **-1.54949** |
| **C** | **-2.88548** | **-2.04049** | **-0.91954** | **-2.81916** | **-2.03649** | **-0.48491** | **-2.87915** | **-2.04216** | **-0.92685** | **-2.83079** | **-2.05063** | **-0.51956** | **-2.84916** | **-2.04305** | **-0.57453** |
| **C** | **-2.14409** | **-1.08286** | **-1.08683** | **-2.0058** | **-1.12732** | **-0.59623** | **-2.14573** | **-1.07959** | **-1.09886** | **-2.01966** | **-1.13808** | **-0.61949** | **-2.02973** | **-1.1361** | **-0.65548** |
| **O** | **-1.92222** | **-0.47987** | **-2.47939** | **-1.36746** | **-0.85062** | **-1.96452** | **-1.95656** | **-0.45978** | **-2.48906** | **-1.36329** | **-0.85871** | **-1.9787** | **-1.34599** | **-0.85615** | **-2.001** |
| **N** | **-1.39649** | **0.87596** | **-2.45556** | **-0.73691** | **0.45107** | **-2.10555** | **-1.42622** | **0.89439** | **-2.46254** | **-0.73375** | **0.44448** | **-2.10977** | **-0.70539** | **0.44324** | **-2.11549** |
| **C** | **-1.46321** | **-0.46034** | **-0.06479** | **-1.63543** | **-0.31432** | **0.45026** | **-1.44565** | **-0.46581** | **-0.08404** | **-1.66728** | **-0.32361** | **0.43195** | **-1.69151** | **-0.32809** | **0.40561** |
| **C** | **-0.13328** | **1.48558** | **1.93109** | **-1.3894** | **2.03402** | **2.80785** | **-0.07764** | **1.45961** | **1.90782** | **-1.46563** | **2.02703** | **2.7926** | **-1.51606** | **2.01057** | **2.78038** |
| **O** | **-1.13239** | **0.04152** | **2.26467** | **-2.14013** | **0.42342** | **2.73209** | **-1.06676** | **0.00927** | **2.244** | **-2.20752** | **0.41287** | **2.70612** | **-2.26841** | **0.40255** | **2.67273** |
| **N** | **-1.63477** | **-0.73345** | **1.28243** | **-2.19452** | **-0.46264** | **1.7197** | **-1.58777** | **-0.75501** | **1.26301** | **-2.24356** | **-0.47383** | **1.69345** | **-2.29226** | **-0.47937** | **1.65554** |
| **O** | **-4.39724** | **4.40161** | **-2.82421** | **-2.43514** | **4.72269** | **-3.83797** | **-4.37243** | **4.42705** | **-2.76803** | **-2.41647** | **4.74101** | **-3.85897** | **-2.32511** | **4.7435** | **-3.88256** |
| **C** | **-3.09147** | **4.49456** | **-3.3924** | **-1.03594** | **4.94543** | **-3.64336** | **-3.04813** | **4.54136** | **-3.29568** | **-1.02073** | **4.96558** | **-3.63634** | **-0.93326** | **4.96361** | **-3.63703** |
| **C** | **-4.19084** | **6.11078** | **-0.94577** | **-3.28335** | **6.17921** | **-2.02161** | **-4.37652** | **6.12363** | **-0.9759** | **-3.32562** | **6.14996** | **-2.02631** | **-3.24198** | **6.17822** | **-2.08014** |
| **N** | **1.6848** | **2.72332** | **-3.11874** | **2.62773** | **2.02175** | **-1.7727** | **1.6585** | **2.72795** | **-3.14291** | **2.6221** | **2.02138** | **-1.72737** | **2.65297** | **1.99797** | **-1.66263** |
| **C** | **2.425** | **4.03518** | **-2.94033** | **3.67376** | **1.94284** | **-2.87834** | **2.4162** | **4.02586** | **-2.9349** | **3.68386** | **1.9425** | **-2.81773** | **3.73446** | **1.9172** | **-2.73346** |
| **C** | **1.91819** | **5.1684** | **-3.09499** | **4.39471** | **2.91777** | **-3.16203** | **1.92617** | **5.16915** | **-3.06774** | **4.40799** | **2.91807** | **-3.09176** | **4.46677** | **2.89055** | **-2.99248** |
| **O** | **3.72266** | **3.90748** | **-2.62473** | **3.76014** | **0.76634** | **-3.52156** | **3.71166** | **3.87516** | **-2.61983** | **3.7806** | **0.76596** | **-3.45899** | **3.83905** | **0.74106** | **-3.3746** |
| **C** | **-1.00154** | **6.96727** | **-3.33102** | **-1.05434** | **8.51816** | **-2.71069** | **-0.97519** | **6.99321** | **-3.35262** | **-0.99298** | **8.49743** | **-2.67715** | **-0.97357** | **8.51756** | **-2.68902** |
| **O** | **-2.1982** | **7.1736** | **-3.49886** | **-2.10736** | **8.88027** | **-3.1909** | **-2.16388** | **7.21183** | **-3.54444** | **-2.10468** | **8.75762** | **-3.08441** | **-2.0267** | **8.85065** | **-3.19054** |
| **H** | **-0.42775** | **5.92048** | **-3.91013** | **-0.22441** | **7.73317** | **-3.43768** | **-0.39603** | **5.93974** | **-3.91502** | **-0.1574** | **7.75721** | **-3.44562** | **-0.11073** | **7.75691** | **-3.40375** |
| **H** | **6.29947** | **8.48278** | **-2.2599** | **2.82255** | **6.45936** | **-5.05511** | **6.7436** | **8.71749** | **-0.59379** | **1.59991** | **5.46469** | **-4.48882** | **1.64559** | **5.40435** | **-4.41574** |
| **H** | **6.73301** | **8.72956** | **-0.54776** | **3.18076** | **4.77757** | **-4.57676** | **7.7077** | **7.61814** | **-1.61156** | **2.93948** | **6.4904** | **-5.00238** | **2.97198** | **6.45003** | **-4.92254** |
| **H** | **7.70073** | **7.65215** | **-1.58556** | **1.51528** | **5.41256** | **-4.50362** | **6.31207** | **8.44432** | **-2.30252** | **3.25445** | **4.79994** | **-4.52353** | **3.3107** | **4.76497** | **-4.44079** |
| **H** | **5.27934** | **7.61485** | **0.75124** | **3.96122** | **4.56022** | **-2.52312** | **5.29945** | **7.62174** | **0.7322** | **3.97119** | **4.55531** | **-2.45522** | **4.01687** | **4.53033** | **-2.36245** |
| **H** | **4.26098** | **5.00237** | **-0.15467** | **4.48385** | **4.94691** | **-0.37724** | **4.27119** | **4.99919** | **-0.13869** | **4.46528** | **4.90999** | **-0.30417** | **4.47358** | **4.90074** | **-0.20073** |
| **H** | **1.99429** | **4.68754** | **0.23732** | **1.53128** | **5.00497** | **-0.80674** | **2.01067** | **4.70061** | **0.20129** | **1.51988** | **4.99019** | **-0.77983** | **1.53165** | **4.98984** | **-0.7315** |
| **H** | **0.62756** | **6.78195** | **1.7216** | **0.96882** | **6.36181** | **1.75275** | **0.62913** | **6.79198** | **1.68209** | **0.90973** | **6.34733** | **1.76955** | **0.8957** | **6.33234** | **1.81854** |
| **H** | **0.29312** | **4.33922** | **2.11557** | **0.39323** | **4.08213** | **1.98089** | **0.35084** | **4.33044** | **2.09763** | **0.30071** | **4.08626** | **2.00723** | **0.29984** | **4.05718** | **2.02253** |
| **H** | **-1.14524** | **5.33982** | **2.0292** | **-1.0321** | **5.11643** | **2.04897** | **-1.10265** | **5.31072** | **2.05175** | **-1.1184** | **5.13168** | **2.0157** | **-1.12276** | **5.0974** | **2.04326** |
| **H** | **4.13212** | **4.29342** | **2.25962** | **5.40839** | **7.20539** | **0.12523** | **4.14713** | **4.32777** | **2.28701** | **5.39273** | **7.15143** | **0.25848** | **5.39068** | **7.15056** | **0.34969** |
| **H** | **5.80592** | **4.61754** | **1.76243** | **5.90799** | **6.61781** | **-1.48106** | **5.8189** | **4.6487** | **1.78167** | **5.91655** | **6.59354** | **-1.3506** | **5.94021** | **6.57291** | **-1.24389** |
| **H** | **4.91903** | **5.80966** | **2.7402** | **4.63708** | **7.8551** | **-1.33024** | **4.93018** | **5.8538** | **2.74235** | **4.65031** | **7.83553** | **-1.19615** | **4.67101** | **7.81536** | **-1.12521** |
| **H** | **1.32675** | **6.6229** | **-1.52291** | **1.08901** | **7.68263** | **-0.95396** | **1.36419** | **6.69065** | **-1.50863** | **1.14768** | **7.62603** | **-0.92466** | **1.11262** | **7.65778** | **-0.87839** |
| **H** | **-0.83922** | **8.5911** | **-1.99368** | **-1.59967** | **8.92334** | **-0.73069** | **-0.8322** | **8.61407** | **-2.01019** | **-1.37547** | **9.17732** | **-0.71624** | **-1.5623** | **8.93801** | **-0.72046** |
| **H** | **1.68481** | **7.91831** | **-3.59269** | **-0.77** | **10.97303** | **-1.67617** | **1.71234** | **7.95158** | **-3.57936** | **1.35526** | **9.90627** | **-1.90131** | **0.32208** | **10.37607** | **-0.05464** |
| **H** | **1.11032** | **9.89512** | **-5.01621** | **0.47073** | **11.79439** | **0.31441** | **1.14803** | **9.92847** | **-5.00701** | **0.42288** | **12.20393** | **-2.20968** | **1.8273** | **11.41108** | **-1.71701** |
| **H** | **-0.10667** | **8.62614** | **-5.17914** | **-0.82295** | **10.68813** | **0.80584** | **-0.0604** | **8.6533** | **-5.18686** | **-0.44456** | **11.04048** | **-3.21883** | **2.09796** | **9.6674** | **-1.6996** |
| **H** | **-0.44511** | **10.0526** | **-4.18569** | **0.86074** | **10.13558** | **0.78936** | **-0.41938** | **10.07679** | **-4.19723** | **-1.18153** | **11.63227** | **-1.72535** | **1.16545** | **10.40933** | **-3.01084** |
| **H** | **2.4574** | **10.16871** | **-2.89301** | **1.65175** | **11.49546** | **-1.90222** | **2.467** | **10.20753** | **-2.8717** | **1.48176** | **11.54119** | **-0.03356** | **-0.46443** | **12.39355** | **-1.27808** |
| **H** | **2.08645** | **9.2065** | **-1.4519** | **1.18422** | **10.22365** | **-3.03249** | **2.08827** | **9.24374** | **-1.43435** | **1.312** | **9.90191** | **0.60856** | **-1.8296** | **11.35294** | **-0.84029** |
| **H** | **0.95123** | **10.44561** | **-2.00546** | **2.07883** | **9.81832** | **-1.55772** | **0.95089** | **10.47587** | **-1.99929** | **-0.08503** | **10.99379** | **0.58363** | **-1.23895** | **11.37949** | **-2.50633** |
| **H** | **-2.43571** | **5.47179** | **0.07201** | **-1.95973** | **5.44629** | **-0.46486** | **-2.31636** | **5.47062** | **0.02309** | **-2.00862** | **5.43307** | **-0.49597** | **-1.9682** | **5.43604** | **-0.49609** |
| **H** | **-5.31108** | **4.35234** | **-0.87065** | **-4.24112** | **4.38414** | **-2.74412** | **-5.33324** | **4.28396** | **-0.85429** | **-4.23705** | **4.35896** | **-2.80849** | **-4.16821** | **4.39077** | **-2.85869** |
| **H** | **-1.94611** | **3.08611** | **2.25795** | **-2.79479** | **3.58269** | **1.61323** | **-1.88294** | **3.05748** | **2.28669** | **-2.85755** | **3.56965** | **1.57504** | **-2.56824** | **2.11547** | **0.59431** |
| **H** | **-2.20384** | **2.0698** | **0.83378** | **-2.51547** | **2.12658** | **0.6583** | **-2.17355** | **2.05002** | **0.86299** | **-2.558** | **2.1143** | **0.62551** | **-2.87447** | **3.56894** | **1.54498** |
| **H** | **-0.24012** | **3.24311** | **-3.9479** | **1.15517** | **2.36399** | **-3.34383** | **-0.27119** | **3.27286** | **-3.94875** | **1.17201** | **2.36207** | **-3.31941** | **1.23507** | **2.3522** | **-3.28065** |
| **H** | **-1.38802** | **3.83519** | **-2.15582** | **-0.18879** | **3.68745** | **-2.14592** | **-1.40323** | **3.8473** | **-2.13974** | **-0.19047** | **3.68483** | **-2.13983** | **-0.14093** | **3.67917** | **-2.1219** |
| **H** | **-2.88797** | **-0.52638** | **-2.99277** | **-2.16762** | **-0.97364** | **-2.69993** | **-2.93481** | **-0.49722** | **-2.97884** | **-2.15322** | **-0.98228** | **-2.725** | **-2.12242** | **-0.97187** | **-2.76257** |
| **H** | **-1.21919** | **-1.10693** | **-3.03572** | **-0.61275** | **-1.61733** | **-2.15794** | **-1.26933** | **-1.08193** | **-3.06998** | **-0.60467** | **-1.6239** | **-2.16258** | **-0.58901** | **-1.62562** | **-2.17341** |
| **H** | **-1.96518** | **1.5846** | **-1.99065** | **-1.33024** | **1.27472** | **-1.99489** | **-1.98369** | **1.59904** | **-1.97976** | **-1.33117** | **1.2668** | **-2.00851** | **-1.29874** | **1.26909** | **-2.02213** |
| **H** | **-0.84513** | **0.30739** | **-0.30362** | **-0.88582** | **0.36227** | **0.3244** | **-0.83506** | **0.30632** | **-0.32737** | **-0.91882** | **0.35605** | **0.31638** | **-0.93571** | **0.34613** | **0.30698** |
| **H** | **-1.29968** | **-0.22779** | **3.3014** | **-2.60639** | **0.13571** | **3.66864** | **-1.21179** | **-0.27279** | **3.28064** | **-2.68576** | **0.12323** | **3.63601** | **-2.76602** | **0.11244** | **3.59226** |
| **H** | **-2.24186** | **-1.60847** | **1.49014** | **-2.75636** | **-1.38286** | **1.83858** | **-2.18851** | **-1.63384** | **1.47324** | **-2.80284** | **-1.39654** | **1.80491** | **-2.86054** | **-1.39823** | **1.75235** |
| **H** | **-4.73631** | **3.37497** | **-2.97993** | **-2.538** | **3.71317** | **-4.24088** | **-4.69851** | **3.40228** | **-2.95433** | **-2.5062** | **3.73876** | **-4.28307** | **-2.4134** | **3.73833** | **-4.29974** |
| **H** | **-5.09501** | **5.08638** | **-3.32209** | **-2.83954** | **5.43351** | **-4.56991** | **-5.05735** | **5.1231** | **-3.26281** | **-2.81138** | **5.46377** | **-4.58385** | **-2.70374** | **5.46217** | **-4.62043** |
| **H** | **-2.89791** | **5.41567** | **-3.65517** | **-0.86401** | **5.90444** | **-3.6017** | **-2.87816** | **5.45514** | **-3.59684** | **-0.84156** | **5.92318** | **-3.61118** | **-0.75898** | **5.92176** | **-3.59116** |
| **H** | **-3.61552** | **6.62938** | **-1.60702** | **-3.37035** | **6.87804** | **-2.75524** | **-3.6009** | **6.62655** | **-1.39792** | **-3.32292** | **6.88057** | **-2.73277** | **-3.28604** | **6.88914** | **-2.80581** |
| **H** | **-5.07475** | **6.59526** | **-0.84318** | **-4.0383** | **6.33037** | **-1.35909** | **-4.44112** | **6.36094** | **0.00734** | **-4.12863** | **6.29457** | **-1.4227** | **-4.02817** | **6.32365** | **-1.45389** |
| **H** | **2.0762** | **2.01101** | **-2.39135** | **2.80908** | **2.94426** | **-1.2248** | **2.04918** | **1.9913** | **-2.43982** | **2.79477** | **2.94417** | **-1.17719** | **2.82012** | **2.91817** | **-1.10636** |
| **H** | **1.94387** | **2.32733** | **-4.10698** | **2.7556** | **1.1902** | **-1.07781** | **1.90533** | **2.35719** | **-4.14404** | **2.74054** | **1.1904** | **-1.03013** | **2.75367** | **1.16393** | **-0.96629** |
| **H** | **4.33348** | **4.71877** | **-2.49875** | **4.42484** | **0.66509** | **-4.27515** | **4.33178** | **4.67806** | **-2.48595** | **4.45595** | **0.66507** | **-4.20308** | **4.52728** | **0.63919** | **-4.10668** |
| **H** | **4.12444** | **2.99266** | **-2.48407** | **3.09767** | **0.02664** | **-3.28999** | **4.10195** | **2.95317** | **-2.49491** | **3.11476** | **0.02624** | **-3.23684** | **3.16624** | **0.00359** | **-3.16697** |
| **H** | **0.49675** | **5.68358** | **-3.59287** | **0.69971** | **7.62095** | **-3.0726** | **0.52066** | **5.69696** | **-3.57958** | **0.77285** | **7.6629** | **-3.09297** | **0.80077** | **7.64022** | **-3.01116** |
